# Incorporating individual historical controls and aggregate treatment effect estimates into a Bayesian survival trial: a simulation study

**DOI:** 10.1186/s12874-019-0714-z

**Published:** 2019-04-24

**Authors:** Caroline Brard, Lisa V. Hampson, Nathalie Gaspar, Marie-Cécile Le Deley, Gwénaël Le Teuff

**Affiliations:** 1 Université Paris-Saclay, Université Paris-Sud, UVSQ, CESP, INSERM, F-94085 Villejuif, France; 2 Service de biostatistique et d’épidémiologie, Gustave Roussy, Université Paris-Saclay, F-94805 Villejuif, France; 3 Statistical Methodology, Novartis Pharma AG, Basel, Switzerland; 4 Gustave Roussy, Département de cancérologie de l’enfant et de l’adolescent, F-94805 Villejuif, France; 5 Centre Oscar Lambret, Unité de Méthodologie et de Biostatistique, F-59000 Lille, France

**Keywords:** Aggregate treatment effect, Bayesian randomised survival trial, Individual control data, Mixture prior, Power prior, Rare disease, Simulation study

## Abstract

**Background:**

Performing well-powered randomised controlled trials (RCTs) of new treatments for rare diseases is often infeasible. However, with the increasing availability of historical data, incorporating existing information into trials with small sample sizes is appealing in order to increase the power. Bayesian approaches enable one to incorporate historical data into a trial’s analysis through a prior distribution.

**Methods:**

Motivated by a RCT intended to evaluate the impact on event-free survival of mifamurtide in patients with osteosarcoma, we performed a simulation study to evaluate the impact on trial operating characteristics of incorporating historical individual control data and aggregate treatment effect estimates. We used power priors derived from historical individual control data for baseline parameters of Weibull and piecewise exponential models, while we used a mixture prior to summarise aggregate information obtained on the relative treatment effect. The impact of prior-data conflicts, both with respect to the parameters and survival models, was evaluated for a set of pre-specified weights assigned to the historical information in the prior distributions.

**Results:**

The operating characteristics varied according to the weights assigned to each source of historical information, the variance of the informative and vague component of the mixture prior and the level of commensurability between the historical and new data. When historical and new controls follow different survival distributions, we did not observe any advantage of choosing a piecewise exponential model compared to a Weibull model for the new trial analysis. However, we think that it remains appealing given the uncertainty that will often surround the shape of the survival distribution of the new data.

**Conclusion:**

In the setting of Sarcome-13 trial, and other similar studies in rare diseases, the gains in power and accuracy made possible by incorporating different types of historical information commensurate with the new trial data have to be balanced against the risk of biased estimates and a possible loss in power if data are not commensurate. The weights allocated to the historical data have to be carefully chosen based on this trade-off. Further simulation studies investigating methods for incorporating historical data are required to generalise the findings.

**Electronic supplementary material:**

The online version of this article (10.1186/s12874-019-0714-z) contains supplementary material, which is available to authorized users.

## Background

Evaluating new treatments for rare diseases in a timely manner can be challenging, even if patients can be recruited across a national or international network of centres [1–7]. Around 5000 to 8000 rare diseases affect in total 30 million people in the European Union [8]. Furthermore, in an era of personalised medicine, efficacy trials of targeted therapies will need to be conducted in increasingly restricted subgroups of patients [9, 10]. Therefore investigators are frequently confronted with the problem of how to design and analyse a randomised clinical trial when the available sample size is small [11].

In the meantime, more and more data are being generated: this may be real world evidence or evidence generated from clinical trials conducted by pharmaceutical companies or academic clinical trials units; evidence may be in the form of individual patient data (IPD) or aggregate information; and data may be accessed through repositories or registries [12]. Evidence on treatment effects may be extracted from a systematic review of the literature. The key question is how can we take advantage of such external information when designing and interpreting a contemporary randomised clinical trial. Assuming that, in the rare disease setting, the standard of care often remains relatively stable over time as treatment options are slow to advance, we can expect some commensurability between the performance of the control therapy in historical studies and the new trial. The term of commensurability, in our context, means that the historical data and new data of the control therapy are consistent with being generated by statistical processes underpinned by similar parameters. The Bayesian approach can be seen as a promising alternative, or complement, to the conventional frequentist approach which enables one to explicitly integrate external data into inferences [7]. In 2006, the Food and Drug Administration published a guideline for the use of Bayesian statistics in medical device clinical trials [13] which highlighted the advantages of using historical data to formulate a prior distribution for a parameter of interest, while insisting on the importance of down-weighting or discounting this information. In 2017, they published a guideline for the use of antibacterial therapies for patients with an unmet medical need for the treatment of serious bacterial diseases which encourages the use of historical information as a control for the trial in some particular situations [14].

First proposed several decades ago [15], the idea of incorporating historical data into new trials has attracted attention as one approach for improving the feasibility and power of trials when only small sample sizes are available. Following the seminal article of Pocock [15] in 1976 which proposed six criteria for selecting historical controls, several methods have been developed [16–24]. A recent review identified different Bayesian and frequentist methods for incorporating historical data into a contemporary trial [25] which may be relevant to paediatric studies, where small sample sizes are a common challenge. For Bayesian methods, the authors distinguished between dynamic methods, where external data are adaptively down-weighted according to their commensurability with the new data, and non-adaptive methods, where a weight for the external data is pre-specified.

Among the different methods available for down-weighting historical data which are also applicable to censored endpoints, we consider two approaches as particularly promising. The first approach is based on a prior which is weighted mixture of an informative prior and a vague component. This approach has been considered by authors in several contexts, for example, to incorporate data from an original geographic region into a bridging trial [26, 27] or historical controls into a new trial [21, 22]. In the latter case, the informative component may be a prior predictive distribution derived from a meta-analysis of historical trials [21]. More generally, a class of ε-contaminated priors may be used to evaluate the sensitivity of an analysis to plausible deviations about an informative prior [28, 29]. The second approach we will consider, called the power prior, was developed by Ibrahim et al. [16]. It consists in raising the likelihood of the historical data to an exponent *α*_0_ representing the degree of commensurability between the historical and new trial data; thus *α*_0_ controls the weight given to the historical data in the posterior distribution. Two versions exist depending on whether *α*_0_ is considered as a fixed value or a random variable. When considered as fixed, the power *α*_0_ can be specified from expert opinion on the plausibility of the commensurability of the historical and new patient data. We have chosen to focus on this latter version since it is easier to communicate to clinicians and does not react to observed differences between the historical and new data, knowing that disentangling true between-trial heterogeneity from random variation may be impossible in the setting of rare diseases.

While a rich array of Bayesian methods for incorporating historical data exist, these approaches are still rarely implemented in practice. A systematic review of clinical trials published before September 2015 identified only 28 trials using Bayesian methods to analyse a censored endpoint, and of these, only four made use of historical data to estimate the treatment effect [30]. In these trials, historical data were incorporated without being down-weighted and without survival regression models being used. One possible explanation for this slow uptake of methods is that the process of selecting a survival model, a method to incorporate the historical data, and a weight reflecting their perceived relevance is complex and open to criticism. This complexity increases when we wish to incorporate both IPD on historical controls and aggregate level data in the form of an estimate of the relative treatment effect obtained from a systematic literature review. Additional methodological concerns are encountered when designing rare disease trials when there will often be scant prior evidence to guide investigators on what weight to assign the historical data and uncertainty about the correct model for the baseline survival hazards.

The current work aims to evaluate the advantages of a Bayesian approach in the setting of a small size randomised phase 2 trial, the Sarcome-13 trial, which is currently in set-up. This trial will evaluate the benefit in terms of event-free survival (EFS) of mifamurtide in combination with post-operative chemotherapy compared with chemotherapy alone. Due to the rare disease setting, we relaxed the alpha level of the one-sided log-rank test to a significance level of 10% for the standard frequentist approach [7, 31], and a pragmatic recruitment target has been set of accruing 105 patients over 3 years (with 2 years of follow-up for the last patient). If this target is met, the power is 80% if the true hazard ratio (HR) is 0.55 (based on 43 events), whereas it decreases to 33% and 20% for a 0.786 (48 events) and 0.886 (50 events), respectively. It is acknowledged that a HR of 0.55 is a very optimistic treatment effect which does not reflect what is anticipated. A HR of 0.786 is considered more realistic and would still be a clinically meaningful effect, although it is clear that the trial will be underpowered to detect HRs of this magnitude. Despite this, the Sarcome-13 investigators believe that in this particular setting, evidence from a small RCT is better than no randomised evidence at all. We seek to augment data from the Sarcome-13 study with relevant historical information to increase the trial’s power to reliably detect smaller, but more plausible, effects.

Due to the form of the available historical data, we propose an approach using power (with fixed weight) [16] and mixture priors [22] to incorporate information derived from historical IPD and aggregate effect estimates, respectively. The operating characteristics of a Bayesian analysis of the Sarcome-13 trial based on these priors are evaluated through a simulation study considering a set of scenarios representing different degrees of commensurability between the historical and new data.

## Methods

### Incorporating two sources of historical information: individual and aggregate level data

In the setting of the Sarcome-13 trial, two sources of historical data were immediately available. The first source of historical information is IPD on patients with high-risk osteosarcoma from the OS2006 trial (NCT00470223) [32]. This trial included 318 patients and used the same backbone chemotherapy as will be used in the Sarcome-13 trial. Selecting from OS2006 all those patients who fulfilled the planned Sarcome-13 eligibility criteria, referred to as the SARC-OS subgroup thereafter, we identified EFS data on 165 patients (73 events) with a median follow-up of 4.1 years [range: 0.2; 5]. We truncated these historical data to match with the duration of the new trial. The second source of historical information is the two relative treatment effects on EFS of post-operative chemotherapy plus mifamurtide versus post-operative chemotherapy alone which was reported by the INT-0133 trial [33, 34]. From the two estimated HRs of this trial (localised [33] and metastatic [34] osteosarcoma), we obtain an overall estimate of the treatment effect (HR = 0.786; 95%CI, 0.63–0.98) after checking for potential heterogeneity. The corresponding estimates of the log hazard ratio ($\widehat{\beta_H}$), which is the parameter that we will consider thereafter, and its variance (*s*^2^) are − 0.241 and 0.012, respectively. This variance is approximately equivalent to what would be obtained if the estimate was based on 329 events (Schoenfeld formula [35]). Details of the Sarcome-13 design and available historical data are described in Additional file 1.

#### Prior distribution for control arm parameters based on IPD

The power prior is a prior formed by raising the likelihood function of the individual historical data ${D}_C^H$ to a power *α*_0_ to control the impact of the historical data on the posterior distribution. Let ***θ*** be the vector of parameters of the survival model chosen to represent EFS on the control arm, and let *π*_0_(***θ***) denote the initial prior, that is, the prior distribution for ***θ*** before the historical data ${D}_C^H$ are observed (where *π*_0_(***θ***) can for example, be taken to be a product of non-informative independent priors for each element of ***θ***). In our case, ${D}_C^H$ represents the IPD on the SARC-OS patients (See Generation of IPD on historical controls sub-section). Thus we define the power prior distribution of ***θ*** as

$$ \pi \left(\boldsymbol{\theta} |{D}_C^H,{\alpha}_0\right)\propto L{\left(\boldsymbol{\theta} |{D}_C^H\right)}^{\alpha_0}\ {\pi}_0\left(\boldsymbol{\theta} \right) $$

where *α*_0_ is a fixed constant with 0 ≤ *α*_0_ ≤ 1. When *α*_0_ = 0, $\pi \left(\boldsymbol{\theta} |{D}_C^H,{\alpha}_0\right)\equiv {\pi}_0\left(\boldsymbol{\theta} \right)$, which means that the historical data are not incorporated into the prior distribution. When *α*_0_ = 1, equal weight is given to the likelihood of the historical data $L\left(\boldsymbol{\theta} |{D}_C^H\right)$ and the likelihood of the new trial data, $L\left(\boldsymbol{\theta} |{D}_C^N\right)$ in the posterior distribution given by

$$ \pi \left(\boldsymbol{\theta} |{D}_C^N,{D}_C^H,{\alpha}_0\right)\propto L\left(\boldsymbol{\theta} |{D}_C^N\right)\ \pi \left(\boldsymbol{\theta} |{D}_C^H,{\alpha}_0\right) $$

where ${D}_C^N$ represents the IPD for control patients from the new trial. Note that the dimension of ***θ*** will depend of the specification of the survival model: for a Weibull model, ***θ = θ***^***w***^ = (*β*_0_, *γ*) with intercept (*β*_0_) and scale parameter (*γ*); and for a piecewise exponential model, ***θ = θ***^***p***^ = (*λ*_1_, *λ*_2_, *λ*_3_) with *λ*_*i*_ the hazard rate for the i^th^ time interval. The likelihood function of the historical data under Weibull or piecewise exponential regression models is detailed in Additional file 2.

#### Prior distribution for the treatment effect based on aggregate data

Prior beliefs about the treatment effect, *β*, defined as the log-HR, are represented by a two-component mixture prior [22] given by

$$ \pi \left(\beta |{D}_{TE}^H,\omega \right)=\omega \times {\pi}_H\left(\beta |{D}_{TE}^H\right)+\left(1-\omega \right)\times {\pi}_0\left(\beta \right) $$

where ${\pi}_H\left(\beta |{D}_{TE}^H\right)$ is an informative component summarising the existing information (${D}_{TE}^H\Big)$ about the parameter *β*. We assume that the prior distribution for the log-HR is normal [36]: ${\pi}_H\left(\beta |{D}_{TE}^H\right)\sim N\left({\mu}_H,{\sigma}_H^2\right)$ with *μ*_*H*_ = log(0.786) and ${\sigma}_H^2=0.012$ based on the INT-0133 published data. Meanwhile, *π*_0_(*β*) is a vague component, which ensures we have some robustness to deal with a prior-data conflict. Based on preliminary simulations, we have set *π*_0_(*β*)~*N*(0, 10). Lastly, *ω* ∈ [0, 1] reflects the prior plausibility of the commensurability of the historical treatment effect estimate and the treatment effect in the new trial: *ω* = 0 indicates a vague prior and *ω* = 1 indicates an informative prior based on the historical data only (See Supplementary Fig. A1, Additional file 3, illustrating how the prior mixture distribution based on the INT-0133 data changes with *ω*).

#### Joint prior distribution for control arm parameters and the treatment effect

Assuming prior opinion on the control arm parameters (***θ***) and the treatment effect (*β*) are independent, the joint prior distribution can be written as

$$ \pi \left(\boldsymbol{\theta}, \beta |{D}_C^H,{D}_{TE}^H,{\alpha}_0,\omega \right)\propto L{\left(\boldsymbol{\theta} |{D}_C^H\right)}^{\alpha_0}\times {\pi}_0\left(\boldsymbol{\theta} \right)\times \left[\omega \times {\pi}_H\left(\beta |{D}_{TE}^H\right)+\left(1-\omega \right)\times {\pi}_0\left(\beta \right)\right] $$

The R code used to compute the joint posterior distribution in the context of a Weibull model is available in Additional file 4.

### Simulation study

The main objective of the simulation study is to evaluate how the operating characteristics of a Bayesian survival trial might vary according to the weights (*α*_0_, *ω*) allocated to the historical data, and to find optimal values of *α*_0_ and *ω* under various scenarios representing different levels of commensurability between the historical and new trial data.

#### Generation of IPD on historical controls

Although observed historical data were available from the OS2006 trial, we decided to work with two hypothetical historical datasets which mimic SARC-OS survival data but generating from two different survival distributions. Working with a simulated dataset gave us explicit control over the distribution of the historical control data, that is, the underlying survival model and model parameters. This meant we could evaluate operating characteristics of the Sarcome-13 design in: a) the ideal situation where the new data are perfectly commensurate with the historical data; and b) when there are conflicts of varying degrees between the distribution of the historical and new control data. We thus generated two hypothetical historical datasets which are similar to SARC-OS data but differ in that one is sampled from a Weibull model and another from a 3–parameter exponential model. Due to random variation, different sets of individual survival times drawn from the same model can lead to very different estimated survival curves, especially when the sample size is small. With this in mind, for each model type, we simulated 10,000 datasets, setting the simulation model parameters equal to the maximum likelihood estimates obtained from fitting the observed SARC-OS data (See Supplementary Fig. A2, Additional file 5) and the sample size identical to the number of SARC-OS patients (*n* = 165). We then empirically selected a replication with estimated survival function close to the underlying model (graphical similarity) and with estimated parameters close to that of observed historical data. This process resulted in two hypothetical historical datasets, depicted in Supplementary Fig. A3, Additional file 6, which served as individual historical control data for the simulation study. We will refer to these datasets as HCW (historical controls sampled from a Weibull distribution) and HCP (historical controls sampled from a piecewise exponential model).

#### Generation of new trial data

We simulated balanced trials with individual survival times sampled from either a Weibull or a piecewise exponential distribution. Parametric survival models were used because they allow modelling the baseline hazard function as a function of parameters estimated from both historical and new individual data on the control arm.

Regarding the generation of survival data on the control arm, we considered various levels of commensurability between the historical and new control data, both in terms of the form of the underlying survival model (Weibull versus 3-parameter exponential) and the values of the model parameters.

**Fig. 1:**
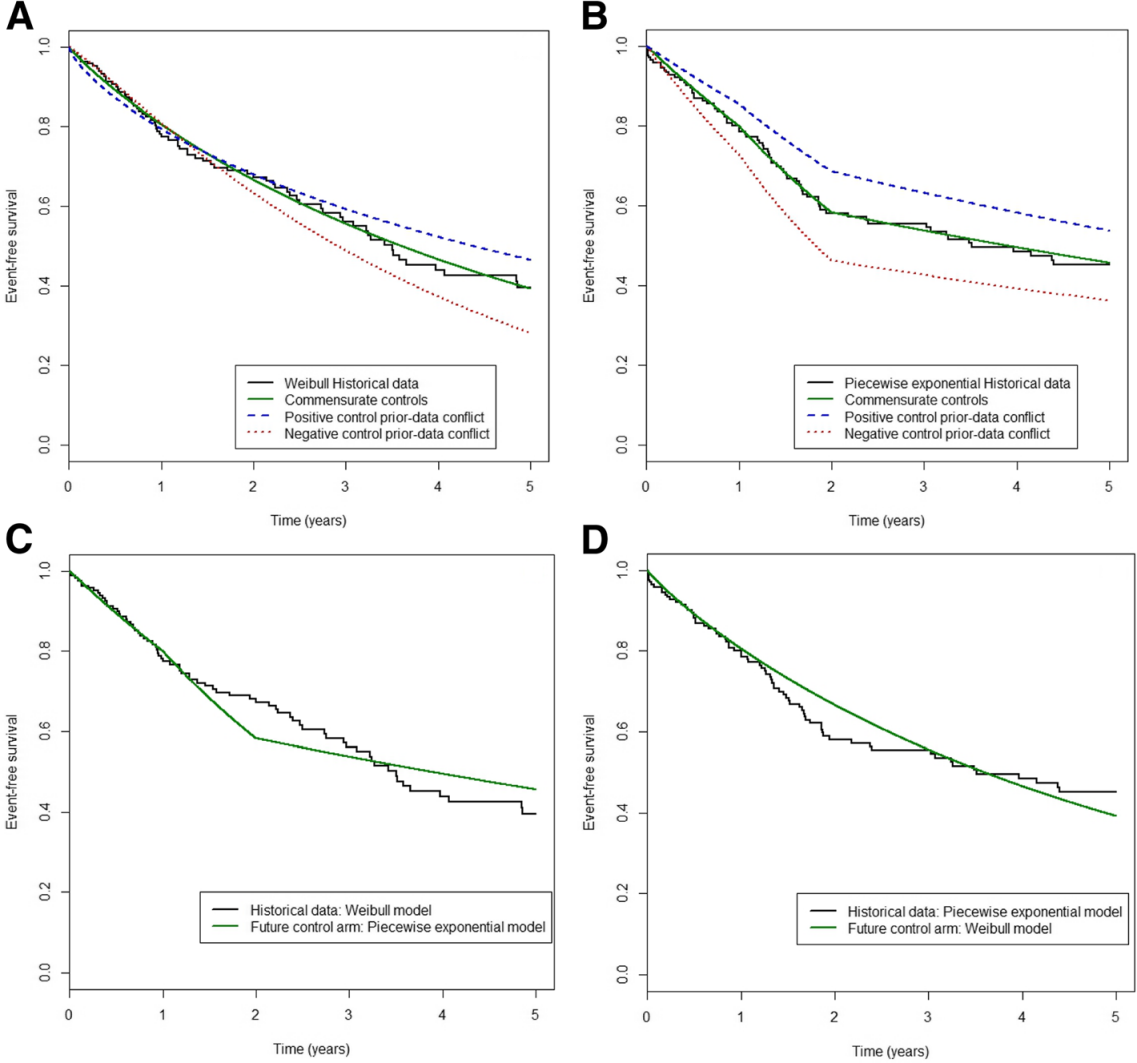
Event-free survival distribution of the historical and new control arm depending of their commensurability. On each panel, the black curve represents the hypothetical historical control survival data (Kaplan-Meier estimates) simulated from a Weibull (left panel) and a piecewise exponential (right panel) distribution. Panels **a** and **b** represent no conflict in terms of underlying survival distribution but possible non-commensurability in terms of parameters (green, red and blue curves for commensurate, negative prior-data conflict and positive prior-data conflict, respectively). Panels **c** and **d** represent non-commensurability in terms of survival distribution (green curve)

In the first set of scenarios (S1-S24), we assumed no conflict between the distributions of the historical and new control data. In scenarios S1-S12 (S13-S24), historical data were taken to be dataset HCW (HCP), and new control data were samples from a Weibull (piecewise exponential) distribution. In scenarios S1-S4 (S13-S16), we set simulation model parameters, ***θ***^***w***^(***θ***^***p***^), equal to the estimates obtained from fitting a Weibull (piecewise exponential) model, to the SARC-OS dataset (see green curves in Fig. 1a and b). In scenarios S5-S8 (S17-S20), simulation model parameters were set as the lower (upper) limits of the 95% confidence intervals for ***θ***^***w***^(***θ***^***p***^) obtained from fitting the SARC-OS dataset (see the red curves in Fig. 1a and b); this approach creates a ‘negative prior-data conflict’, where the prognosis of the new controls is worse than that of the historical controls. Conversely, in scenarios S9-S12 (S21-S24), model parameters were taken to be the upper (lower) limits of the 95% confidence intervals for ***θ***^***w***^(***θ***^***p***^) (see blue curves in Fig. 1a and b), thus creating a ‘positive prior-data conflict’, where the prognosis of the new controls is better than that of the historical controls.

Simulation scenarios S25-S32 characterise cases where there is a conflict between the historical and new control data in terms of the underlying survival distribution. Scenarios S25-S28 (S29-S32) take the historical control data to be the hypothetical dataset generated from a Weibull (piecewise exponential) model, whereas the new control data are samples from a piecewise exponential (Weibull) distribution (Figs. 1c-1d).

Regarding the treatment effect in the new trial, *β*, four cases representing different degrees of commensurability with the historical aggregate data (${D}_{TE}^H$) were considered: (i) no treatment effect, i.e. *β* = ln(1) (‘null’ scenario; S1, S5, S9, S13, S17, S21, S25 and S29), (ii) a treatment effect inferior to the historical treatment effect derived from the pooled INT-0133 estimates, i.e. $\beta =0.5\times \widehat{\beta_H}=\ln (0.886)$ (‘disappointing effect’ scenario; S2, S6, S10, S14, S18, S22, S26 and S30), (iii) a treatment effect equal to the estimated historical treatment effect, i.e. $\beta =\widehat{\beta_H}=\ln (0.786)$ (‘historical effect’ scenario; S3, S7, S11, S15, S19, S23, S27, and S31), and (iv) a treatment effect equal to the target effect of the Sarcome-13 trial which is superior to the historical treatment effect, i.e. *β* = ln(0.55) (‘anticipated effect’ scenario; S4, S8, S12, S16, S20, S24, S28, and S32).

In summary, we considered eight configurations of differences between the survival distribution and model parameters which generated the historical and new control data. Furthermore, we investigated four cases of discrepancies between the historical and new treatment effect, leading to a total of 32 simulation scenarios reflecting different degrees of commensurability between the historical information and new trial data (Table 1; see Supplementary Figs. A4-A6, Additional file 7, for a graphical representation). For each of the 32 scenarios, 5000 trials were simulated with fixed sample size *n* = 105, as in the planned Sarcome-13 trial, with 1:1 randomisation between trial arms and a uniform censoring rate of 5%.

**Table 1:** Summary of the 32 scenarios considered for the simulation of the historical controls and new trial data

Scenario	Survival distribution of historical controls^a^	Generation of new data		
		Survival distribution^b^	Parameters	
			Control arm	Treatment effect^c^
S1	Weibull	Weibull	Commensurate controls	Null
S2				Disappointing
S3				Historical
S4				Anticipated
S5	Weibull	Weibull	Negative prior-data conflict	Null
S6				Disappointing
S7				Historical
S8				Anticipated
S9	Weibull	Weibull	Positive prior-data conflict	Null
S10				Disappointing
S11				Historical
S12				Anticipated
S13	Piecewise exponential	Piecewise exponential	Commensurate controls	Null
S14				Disappointing
S15				Historical
S16				Anticipated
S17	Piecewise exponential	Piecewise exponential	Negative prior-data conflict	Null
S18				Disappointing
S19				Historical
S20				Anticipated
S21	Piecewise exponential	Piecewise exponential	Positive prior-data conflict	Null
S22				Disappointing
S23				Historical
S24				Anticipated
S25	Weibull	Piecewise exponential	Commensurate controls	Null
S26				Disappointing
S27				Historical
S28				Anticipated
S29	Piecewise exponential	Weibull	Commensurate controls	Null
S30				Disappointing
S31				Historical
S32				Anticipated

^a^Survival distribution used to generate individual historical controls

^b^Survival distribution used to generate individual patient data for the control arm of the new trial

^c^Null, Disappointing, historical and anticipated effects correspond to a hazard ratio of 1, 0.886, 0.786, and 0.55 in the new trial, respectively

#### Trial analysis

The data generated according to scenarios S1-S12 and S13-S24 were analysed by fitting a Weibull or a 3-parameter exponential Bayesian model, respectively. For scenarios S25-S32 in which the historical and new control data are samples from different distributions, we evaluated the impact of the analysis model by comparing the results obtained when data were analysed using the model consistent with the underlying distribution of the historical control data or with the distribution used to generate the new data.

Prior distributions for the Weibull model parameters, *β*_0_ and *γ*, were set as Normal(0, 10000) and Inverse Gamma(0.0001, 0.0001), respectively. Concerning the piecewise exponential model, we stipulated Normal(0, 10000) prior distributions for *λ*_1_, *λ*_2_, and *λ*_3_ (log scale). These priors were used whenever a Weibull or a piecewise model was used for the trial analysis. The impact of incorporating historical data into prior distributions was evaluated by performing analyses under different configurations of the weights *α*_0_ and *ω*: we considered pairs of weights with *α*_0_ ∈ {0, 0.3, 0.6, 1} and *ω* ∈ {0, 0.1, 0.2, 0.4, 0.6, 0.8, 1}. Different values for the variance $\left({\sigma}_H^2\right)$ of the informative component of the mixture prior for *β* were also considered to represent situations where the amount of historical information is larger, equivalent, or smaller than the information that will be generated by the new trial for scenarios S1 to S12. These values were ${\sigma}_H^2=\left\{{s}^2,5{s}^2\mathrm{and}\ 15{s}^2\right\}$ with *s*^2^ = 0.012 (variance of the historical effect estimate), and thus equivalent to 329, 66, and 22 events, respectively (expected event numbers calculated according to Schoenfeld formula [35]). The main results correspond to ${\sigma}_H^2={s}^2$, equivalent to 329 events.

Bayesian survival models were fitted using Markov chain Monte Carlo. We ran one chain, sampling using a Metropolis-Hastings algorithm [37] for 20,000 iterations with a ‘burn-in’ period of 5000 iterations, leaving 15,000 samples for posterior inferences. Convergence to the stationary distribution was assessed by Geweke’s diagnostic test.

We evaluated the frequentist operating characteristics of the proposed Bayesian survival trial design assuming that the following rule will be used to make final treatment decisions: post-operative chemotherapy plus mifamurtide will be deemed superior to post-chemotherapy alone if the posterior probability of a HR lower than one exceeds 0.9. The means of the posterior distributions of *β* was recorded for each simulated trial.

#### Metrics of the simulation study

For each simulation scenario, we estimated the bias of the posterior estimate of the treatment effect as the difference between the sample mean of the 5000 means of the posterior distributions of *β* and the scenario-specific true treatment effect. The empirical standard deviation (SD) and the root mean square error (RMSE) of these estimates were calculated as measures of precision and accuracy, respectively. By counting the number of positive conclusions among the simulated trials, we could compute the frequentist type I error rate (in null scenarios, i.e. HR = 1) and the frequentist power of a Bayesian decision (in all other scenarios in which the new treatment is superior to the contemporary performance of control, i.e. HR < 1).

All simulations and Bayesian analyses were performed using a customised program written in R 3.3.1 [38] calling the MCMCpack [39] and LearnBayespackages [40]. The R code to perform the Bayesian survival analysis with a power prior and a mixture prior assuming a Weibull distribution is provided in Additional file 4. The code for the whole simulation study is available upon request from the authors.

## Results

### Impact of including historical aggregate treatment effect only (***α***_**0**_ **= 0**)

**Fig. 2:**
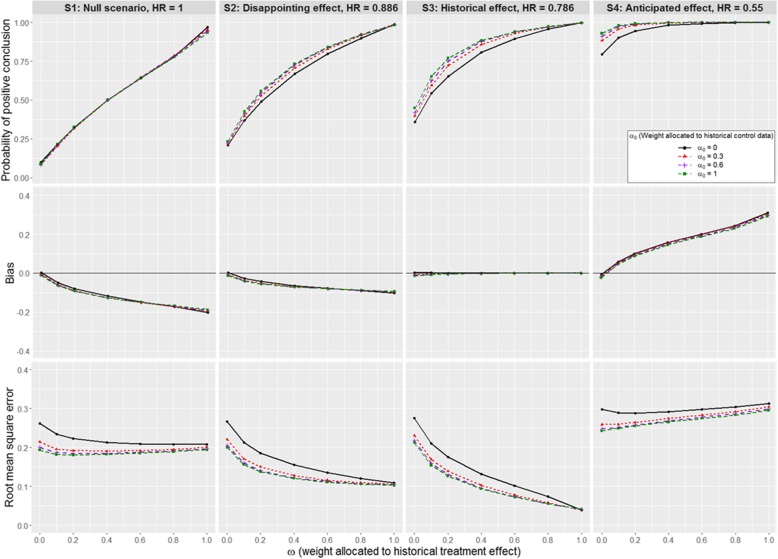
Impact of *α*_0_ and *ω* on the operating characteristics for scenarios S1 to S4. A Weibull distribution is used for the historical and new data, with ${\boldsymbol{\sigma}}_{\boldsymbol{H}}^{\mathbf{2}}$ equivalent to 329 events. We assume that historical and new control arm are commensurate

**Fig. 3:**
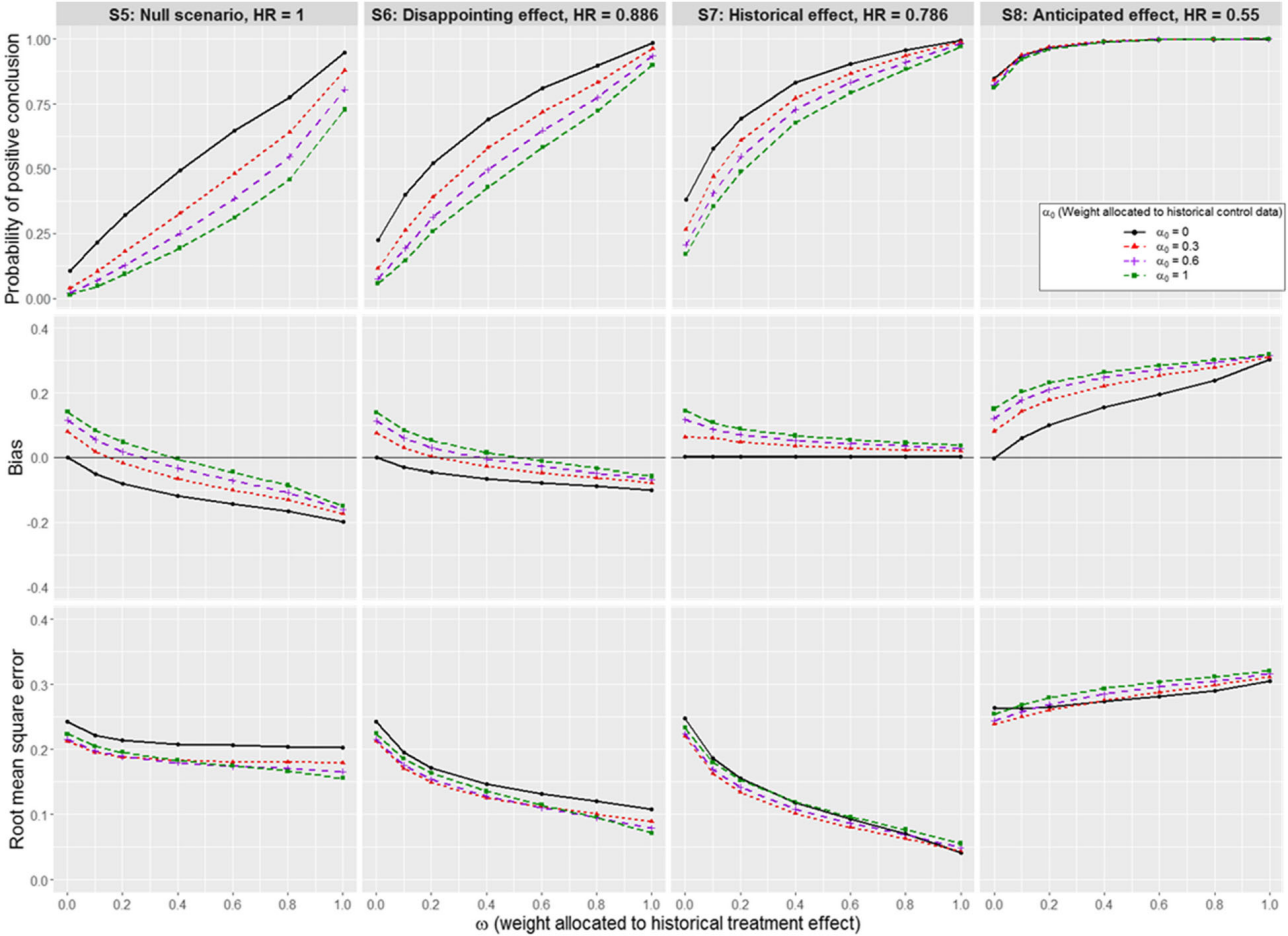
Impact of *α*_0_ and *ω* on the operating characteristics S5 to S8. A Weibull distribution is used for the historical and new data, with ${\sigma}_H^2$ equivalent to 329 events. We assume a negative prior-data conflict between historical and new control data

**Fig. 4:**
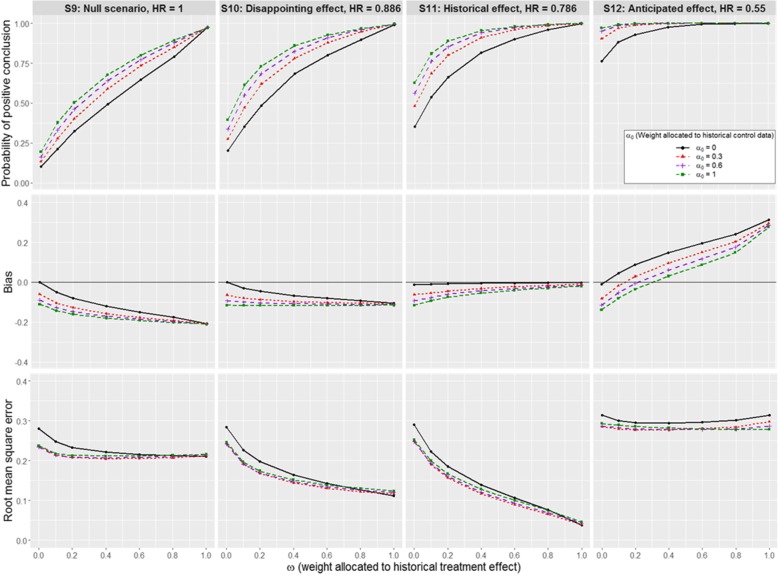
Impact of *α*_0_ and *ω* on the operating characteristics for scenarios S9 to S12. A Weibull distribution is used for the historical and new data, with ${\sigma}_H^2$ equivalent to 329 events. We assume a positive prior-data conflict between historical and new control data

The impact of including only historical aggregate information on the treatment effect and excluding the historical controls can be determined by looking at results when *α*_0_ = 0. Figures 2, 3 and 4 summarise the main findings of simulation scenarios S1-S12 with respect to type I and II errors, bias and RMSE of the Bayesian posterior treatment effect estimate. In these scenarios, the historical controls were generated by sampling from Weibull distributions; prior-data conflicts arise either due to differences between corresponding parameters of these Weibull distributions or because the treatment effect underlying the new trial differs from the historical estimate. Detailed results for scenarios S1-S12 are listed in Supplementary Tables A1-A3, Additional file 8. Similar findings hold when data are samples from piecewise exponential models; results for scenarios S13-S24 are listed in Supplementary Tables A4-A6, Additional file 9.

Setting *α*_0_ = 0, the effect of increasing *ω* is illustrated by the black curves in Figs. 2, 3 and 4. In scenarios S1-S4, S5-S8 and S9-S12, the contemporary control data are samples from models with different baseline hazards: these differences have no major impact, so that similar patterns of results are observed across null scenarios S1, S5 and S9. The same applies to disappointing effect scenarios S2, S6, and S10; historical effect scenarios S3, S7, and S11; and anticipated effect scenarios S4, S8, and S12.

Incorporating historical information on the treatment effect leads to important gains in power both when the treatment effect in the new trial is less than the historical estimate (power increases from 20.8% for *ω* = 0 to 98.9% for *ω* = 1 in scenario S2) and when it exceeds the historical estimate (power increases from 79.4% for *ω* = 0 to 100% for *ω* = 1 in scenario S4). In most scenarios, the largest gains in power are made by increasing *ω* from 0 to 0.2 or 0.4; increasing *ω* further tends to result in smaller gains. The increases in power seen under alternative effect scenarios must be balanced against the risk that we will inflate the type I error rate if, in fact, the new treatment is no better than standard treatment in the new trial: in null scenario S1, the type I error rate increases rapidly with *ω*, reaching 96.9% for *ω* = 1.

In the scenarios where the new treatment effect is equal to the historical effect estimate (scenario S3), incorporating historical data on the treatment effect leads to no bias in the treatment effect estimate. If the treatment effect in the new trial is worse than indicated by the historical data (S1-S2) the new effect is overestimated, where the magnitude of the bias increases with *ω*. Conversely, if the treatment effect in the new trial exceeds the historical estimate (S4), incorporating the historical data causes to underestimate the new effect.

When *ω* = 0, the average empirical SD of the posterior treatment effect estimate differs slightly across the simulation scenarios due to the different number of events which are expected to occur in the new trial in each case (fewer events expected when there is a larger treatment effect or the baseline hazards of death are smaller). Despite this, in all scenarios, the average precision of the posterior estimate increases as *ω* increases from 0 to 1. In contrast, the manner in which the RMSE changes with *ω* depends on the simulation scenario. For example, in the disappointing and historical effect scenarios S2 and S3, the RMSE decreases rapidly as *ω* increases: the relative change in RMSE is − 59% in S2 and − 86% in S3 as *ω* increases from 0 to 1. Smaller decreases in the RMSE are observed in null scenario S1 (relative change of − 21% as *ω* increases from 0 to 1). Meanwhile, in anticipated effect scenario S4, small changes in the RMSE are observed as *ω* varies between 0 and 1, but whereas a slight decrease results from increasing *ω* from 0 to 0.2, consistent (small) increases are observed as *ω* increases beyond 0.4, reflecting the trade-off between increased precision and increased bias.

#### Choice of ***ω*** value for Sarcome-13 trial

When choosing how much weight to assign to the historical information for the treatment effect, we must consider the impact of increasing *ω* on the bias, precision, and accuracy of the treatment effect estimator, as well as the power and type I error rate of the trial. Overall with this in mind, we recommend setting *ω* = 0.1 when *α*_0_ = 0 if the variance of the informative component of the mixture prior for *β* is equivalent to 329 events. This choice of *ω* leads to a type I error rate of 21.6% (S1). However, the scenario of no treatment effect on EFS was deemed unlikely by the investigators. This is why, in the setting of Sarcome-13 trial, we accept this level of type I error. Setting *ω* = 0.1 enables substantial gains in power as *ω* increases from 0 to 0.1 (from 35.9 to 54.5% in scenario S3, for example) and accuracy (RMSE decreases from 0.275 to 0.210 in scenario S3).

#### Different variances of the informative component of the mixture prior

**Fig. 5:**
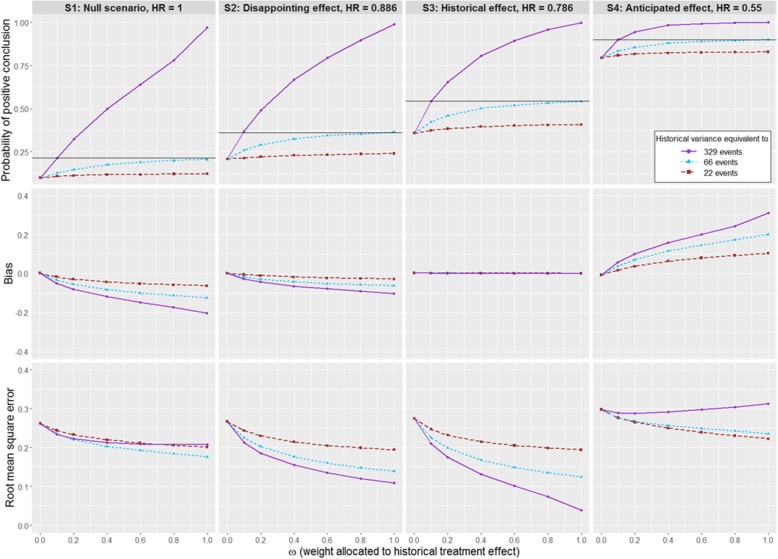
Impact of ω, for *α*_0_ = 0, on the operating characteristics for scenarios S1 to S4. A Weibull distribution is used for the historical and new data. We assume here commensurability between historical and new control arm, with various values of ${\sigma}_H^2$ equivalent to 329, 66 and 22 events, The horizontal line represents the metric for *α*_0_ = 0 and ω = 0.1

Different recommendations for *ω* may apply depending upon the amount of historical information available for the treatment effect. When ${\sigma}_H^2={s}^2,\kern0.5em 5{s}^2$ or 15*s*^2^, we observe similar trends in the properties of the treatment effect estimator and trial operating characteristics as *ω* increases from 0 to 1, although the impact of changes in *ω* decreases as ${\sigma}_H^2$ increases. This is especially true for power, as illustrated in Fig. 5 for scenarios S1 to S4 (details of the results for all scenarios are in Supplementary Tables A7-A12, Additional file 10). For any specific value of ${\sigma}_H^2$, the optimal choice of *ω* must balance the competing aims of increasing power and controlling bias. For example, suppose that the historical information for *β* is such that ${\sigma}_H^2=5{s}^2$. Then in this case, if the survival distribution is as specified in scenarios S2-S4, to achieve the same gain in power as would be attained by setting *ω* = 0.1 when ${\sigma}_H^2={s}^2$, we must set *ω* = 1. However, the risks associated with setting *ω* = 1 when ${\sigma}_H^2=5{s}^2$ are higher than those associated with setting *ω* = 0.1 when ${\sigma}_H^2={s}^2$, since we see a larger bias in the treatment effect estimator when the historical and new data are not commensurate. For instance, in scenario S2, bias is estimated at − 0.0275 when ${\sigma}_H^2={s}^2$ and *ω* = 0.1 versus − 0.0627 when ${\sigma}_H^2=5{s}^2$ and *ω* = 1; in scenario S4, the bias is + 0.0582 versus + 0.2014. When the amount of historical information is reduced $\left({\sigma}_H^2=15{s}^2\right)$, the gains in power possible by incorporating historical information for *β* are negligible, although bias still increases with increasing *ω*. This contrasts with the fact that gains in precision are still possible by increasing *ω* even for large values of the variance ${\sigma}_H^2$.

### Impact of including historical control data only (***ω*** **= 0**)

We begin by summarising results for scenarios S1-S12 (assuming historical controls and the new trial data are samples from a Weibull model) for *ω* = 0 and various choices of *α*_0_, which can be read off as the y-intercepts of the curves shown in Figs. 2-4 and from Supplementary Tables A1-A3, Additional file 8.

#### Historical and new control data are commensurate

Fixing *ω* = 0, we only incorporate historical control data into the new trial analysis, which leads to a limited increase in power when the historical and contemporary controls are commensurate (S1-S4, Fig. 2). The magnitude of the gains in power possible by increasing *α*_0_ from 0 to 1 depends on the true treatment effect, with power increasing from 20.8 to 23.3% in S2; from 35.9 to 44.8% in S3; and from 79.4 to 92.9% in S4. Power is increased by shifting *α*_0_ from 0 to 0.3, with limited gains possible for higher *α*_0_ values. Increasing *α*_0_ is also associated with a slightly improved control of the type I error rate; for example, the false positive error rate decreases from 10 to 8.7% as *α*_0_ changes from 0 to 1 in S1. The main benefit of incorporating historical control data is a gain in precision and accuracy of the treatment effect estimate (RMSE decreases from 0.275 to 0.212 in S3). As we incur a slight unexpected increase in bias when increasing *α*_0_, we extended the simulations presented here to consider the case that the new trial recruits 2000 patients (data not shown). In these additional scenarios, we observed no increase in bias when increasing *α*_0_, which suggests that the small bias observed here when the new trial recruits only 105 patients is due to the small sample size.

#### Negative prior-data conflict between historical and new control data

Focusing now on scenarios where survival for new controls is worse than for historical controls (S5-S8, Fig. 3), we observe a reduction in the probability of a positive conclusion in all scenarios, leading to an improved control of the type I error rate (error rate is 10.4 and 1.4% when *α*_0_= 0 and 1, respectively, in S5) but also a loss in power in alternative effect scenarios. As we allocate more weight to the historical information, power decreases from 22.4 to 5.6% (− 75%), from 38.2 to 16.8% (− 56%), and from 84.9 to 81.1% (− 4.5%), in the disappointing (S6), historical (S7) and anticipated (S8) treatment effect scenarios, respectively. These losses in power are due to the fact that the treatment effect is underestimated, and the magnitude of the bias increases with *α*_0_. This can be explained by noting that increasing *α*_0_ amounts to augmenting the contemporary control data with an increasing number of pseudo-observations on control patients who have an improved prognosis which reduces the difference between the control and experimental arm in the analysis of the new trial. The increase in precision seen across scenarios S5-S8 is similar to the increase seen in scenarios S1-S4 when historical and contemporary controls are commensurate. The increase in bias and precision seen when increasing *α*_0_ results in a minor improvement in RMSE for *α*_0_ = 0.3 that disappears for higher *α*_0_. Thus, in such settings, there is minor advantage to incorporating historical control data in terms of RMSE and we observe a detrimental effect in terms of power.

#### Positive prior-data conflict between historical and new control data

Similar results in terms of precision are observed for scenarios S9-S12, where survival of new controls is better than historical data. The type I error rate increases with increasing *α*_0_ from 10.2 to 19.4% (Fig. 4). Power also increases as we incorporate the historical control data. In scenarios S9-S12, we overestimate the treatment effect when incorporating the historical control data: augmenting the contemporary controls with patient data on controls who have a worse prognosis increases the estimate of the treatment difference.

#### Results when considering a piecewise exponential distribution

Overall, similar results are observed when historical and new control data follow a piecewise exponential survival distribution with different degrees of commensurability between the baseline survival of the historical and new controls (S13-S24) (Supplementary Tables A4-A6, Additional file 9). In small samples, the bias in the treatment effect estimate seen when the historical and new controls are commensurate is more marked when control outcomes are samples from a piecewise exponential model. However, this bias disappears for larger new trial sample sizes (data not shown).

### Impact of including both individual historical control data and aggregate historical information on the treatment effect

We first suppose that the historical controls and new trial data follow a Weibull distribution, and that the variance of the informative component of the mixture prior for the treatment effect is equivalent to what would be obtained if we observed 329 events.

In this situation, assuming we incorporate both the historical controls and existing information on the treatment effect into the new trial analysis by setting *ω* > 0 and *α*_0_ > 0, we find that changes in the probability of a successful trial outcome, and the empirical SD and RMSE of the posterior treatment effect estimate appear to be largely driven by changes in *ω* rather than *α*_0_. The patterns of changes in operating characteristics seen as *ω* increases are consistent across different values of *α*_0_. In many scenarios, the impact of *α*_0_ on different performance metrics is smaller under higher values of *ω*. It is explained by the fact that *ω* is assigned to the information on the treatment effect. In scenario S3, when all historical data (historical controls and treatment effect prior) are commensurate with the new trial data, power is respectively 35.9% when *α*_0_ = 0 and *ω* = 0; 44.8% when *α*_0_ = 1 and *ω* = 0; and 99.7% when *ω* = 1, independent of the value of *α*_0_. In terms of type I error, we observed an overall inflation with increasing *ω*, whatever the level of commensurability between historical and new control, and the values of *α*_0_. However, we note a positive impact of increasing *α*_0_ in negative-prior conflict (S5): for instance, for *ω* = 1, type I error varied from 95% for *α*_0_ = 0 to 73% for *α*_0_ = 1. However, the type I error rapidly increases when increasing *ω* counterbalancing the small gain obtained with the increase of *α*_0_.

#### Choice of weighting parameters for the Sarcome-13 trial Bayesian analysis

Using the parameter configuration *α*_0_ = 0 and *ω* = 0 as a benchmark, we find that incorporating historical data by setting *α*_0_ = 0.3 and *ω* = 0.1 is an acceptable trade-off. It increases power from 20.8 to 39.5% in the disappointing effect scenario (S2); from 35.9 to 61.3% in the historical effect scenario (S3); and from 79.4 to 95.7% in the anticipated effect scenario (S4). The configuration α_0_ = 0.3 and ω = 0.1 also leads to an increased type I error rate at 20.4% (S1). However, as already written, the scenario of no effect of mifamurtide on EFS was deemed unlikely by the investigators. This is why in the setting of Sarcome-13 trial, we accepted this level of type I error. The Supplementary Fig. A7, Additional file 11 presents the posterior distribution of the log-hazard ratio depending on the values of the weighting parameters.

#### Results when considering a piecewise exponential distribution

Similar conclusions hold when historical and new trial follow a piecewise exponential distribution and are analysed using a Bayesian piecewise exponential model (Supplementary Tables A4-A6, Additional file 9).

### Impact of including individual historical data in conflict with new control data in terms of survival distributions

Table 2 summarises the impact of incorporating historical control data only (fixing *ω* = 0 and varying *α*_0_) when the historical control data follow a Weibull survival distribution and the new trial data follow a piecewise exponential distribution (Scenario S27 with HR = 0.786). Simulated data in this scenario were analysed using either a Weibull model (W/P/W) or a piecewise exponential model (W/P/P). Overall, the choice of the analysis model had a limited impact. This may be explained by the fact that these historical datasets were, by construction, relatively similar even if generated from different survival distributions. However, in scenario S27 there is a slightly smaller bias for the treatment effect if the analysis model is consistent with the distribution of the new trial data. Compared with scenario S3 where there is no conflict between the survival distributions of the historical and new data, and where data are analysed with a Weibull model (W/W/W), incorporating historical data when there is a conflict in survival distribution leads to increases in bias and losses in accuracy. Similar results were observed for scenarios with different underlying HRs (detailed in Supplementary Tables A13-A14, Additional file 12).

**Table 2:** Impact of individual historical data in conflict with new data in terms of survival distribution for S27^a^

	W/P/W				W/P/P				W/W/W			
α_0_	Bias	SD	RMSE	Power	Bias	SD	RMSE	Power	Bias	SD	RMSE	Power
0	−0.0142	0.292	0.298	0.358	−0.0087	0.287	0.292	0.345	0.0022	0.275	0.275	0.359
0.3	−0.0541	0.258	0.255	0.447	−0.0366	0.256	0.248	0.416	−0.0070	0.245	0.231	0.396
0.6	−0.0736	0.245	0.248	0.505	−0.0544	0.243	0.240	0.464	−0.0111	0.233	0.218	0.418
1	−0.0883	0.235	0.247	0.554	−0.0702	0.235	0.239	0.518	−0.0141	0.223	0.212	0.448

^a^Results correspond to scenario S27 defined with Weibull survival distribution for the historical control data and piecewise exponential distribution for the new data with HR = 0.786 and analysed with ω = 0 either with a Weibull model (W/P/W) or with a piecewise exponential model (W/P/P). These results are compared to scenario S3, given as a benchmark and defined by commensurate historical and new control data which follow a Weibull distribution, and are analysed with a Weibull model (W/W/W)

When historical control data follow a piecewise exponential distribution and new data follow a Weibull survival distribution, both types of model (Weibull and piecewise exponential) lead to very similar results (see Supplementary Tables A15 for P/W/P and A16 for P/W/W, Additional file 12).

## Discussion

This paper proposes using a power prior (with fixed power parameter) and a mixture prior to incorporate simultaneously individual historical controls and aggregate treatment effect estimates into the Bayesian analysis of a new survival trial. Trial operating characteristics under this approach were evaluated through simulations. Properties varied according to the weights assigned to each source of historical information, the variance of the informative and vague component of the mixture prior and the level of commensurability between the historical and new data. Indeed, in one hand, a high inflation of type I error is observed which challenges the benefit of using historical data, but this type I error is computed for the scenario of no treatment effect deemed unlikely, and with, however, a gain in terms of precision. In the other hand, an increase in power is observed in the other scenarios, even if a more stringent decision threshold is set, such as P(HR < 1) > 95%, to control the type I error rate at 10% (data available on request). The incorporation of historical individual control data had a very little impact in terms of power. Incorporate these data could be a questionable choice given the potential risk to bias results. However, these data enable us to gain in precision and thus, as we are confident that these data will be commensurate with Sarcome-13 control data, we decided to incorporate them. We identified empirically values for *α*_0_ and *ω* which is a reasonable trade-off between power, bias and accuracy for small studies with a set-up similar to the Sarcome-13 trial. In addition to the evaluation of the weight allocated to the historical data, we also evaluated the impact of the model uncertainty i.e. when individual historical control data used to specify the analysis do not fit well the new controls (shape of the modelling). In our simulations, whatever the scenarios, choosing a piecewise exponential compared to a Weibull model for the Bayesian analysis of the new trial did not provide any advantage in terms of bias, precision or power. These results may be explained by the similarity of the distribution of the hypothetical historical data set compared to that of the generated datasets for the new trial in the setting of Sarcome-13. Incorporating historical control data requires survival modelling; the definition of survival modelling type may be challenging due to the uncertainty that often surrounds the shape of the survival distribution in rare diseases.

All these results were obtained from a Bayesian approach using two different priors (power prior and mixture prior) for incorporating two different types of historical data. We could use similar priors for both types of data but our pragmatic approach has two main advantages: a) applying a mixture prior to the aggregate estimate allows to have a robustifying component which allows to better respond to prior-data conflict, b) applying a power prior (with fixed power parameter) to individual patient data is less complex, because a mixture prior would require asymptotic assumption about the form of joint posterior distribution of the control survival model parameters. As there will often be several parameters characterising survival on control, the power prior seems a more parsimonious way of incorporating these data.

The impact of incorporating historical data of the same type into the analysis of a new trial is not new and has previously been evaluated for the cases of binary [21, 23, 24, 41, 42] and survival outcomes [43], with similar findings to those presented here. Focusing on binary endpoints, Cuffe [41] highlights the risks to power and unbiased estimation of response rates of incorporating historical controls if these are not commensurate with new data which is in agreement with our work. Furthermore, Cuffe points out that if we want to ensure control of the type I error rate accounting for possible prior-data conflicts, we may not increase power and even reduce it. More recently, Li et al. [23] present an empirical meta-analytic predictive prior to better adjust the weight of historical data according to the degree of prior-data conflict. They compare their method to the meta-analytic predictive prior and its robust version presented by Schmidli et al. [21]and show that their method better control the type I error rate in case of heterogeneity between historical and new study. They also highlight the difficulties of determining the weight parameter *ω* in the robust meta-analytic predictive prior as this would be based on the investigators confidence in the relevance of the historical data. When only a single historical study is available, Gravestock et al. [24] propose an empirical Bayes approach to power prior construction in order to adaptively respond to prior-data conflict. This approach performs well compared to a full Bayes approach or a fixed parameter approach where the choice of the weighting parameter *α*_0_ is not straightforward. In the context of censored endpoints, Van Rosmalen et al. [43] compared different methods for including individual patient data from the control arms of several historical trials. These authors showed that accounting for between-trial heterogeneity is necessary to take full advantage of the historical data. More generally, Neuenschwander et al. [18], Gsteiger et al. [44], Schmidli et al. [21] adopt a meta-analytic model to describe between-trial differences in a key parameter; a prior distribution for the parameter in a future trial is derived assuming parameters in the historical and future studies are exchangeable. As stated in the background section, given the limited amount of historical data likely to be available when planning a rare disease trial, we do not attempt to model between-trial differences in key parameters. Instead, we propose accounting for potential differences, that is, prior-data conflicts, by discounting the historical control data (using a power prior with fixed power parameter) and adopting a robust mixture prior for the treatment effect. We speculate that using a power prior with a dynamic, rather than a fixed power parameter would imply improved type I error rate control as the analysis would respond quicker to a prior-data conflict arising because the new trial data are less promising than the treatment effect prior. However, a dynamic version of the power prior may lead to greater penalisation of the historical control data in the setting of small trials when larger differences between the historical and new controls can be observed due to random variation, rather than true differences between study-specific parameters [45, 46]. Furthermore, it may also lead to reduced borrowing when new and historical datasets are commensurate [19]. The evaluation of the impact of fixed versus adaptive prior in the setting of rare diseases with a high level of uncertainty is worth of further investigations.

Regarding the mixture prior for the log hazard ratio, selecting suitable values for *ω* and the variance of the vague mixture component is not straightforward, and both will impact trial operating characteristics. All simulations in this paper were performed setting the vague component to be a normal distribution with mean zero and variance 10. Mutsvari et al. [22]note the importance of an appropriate choice of variance for the vague component to ensure adequate discounting of the historical information in the event of a prior-data conflict. If this variance is excessively large, the mixture prior will have very heavy tails, placing prior mass on treatment effects with implausibly large absolute values. This leads to little down-weighting of the historical data, even if there is a clear prior-data conflict. This is why we chose, after some simulations, a variance of 10 that seemed to be a good trade-off. In practice, we would recommend statisticians planning to use a mixture prior for a new Bayesian clinical trial should run simulations to calibrate the variance of the vague prior component and its weight, (1 − *ω*), to find values producing favourable trial operating characteristics.

Even though we attempted to make the simulation study wider in considering a various set of scenarios, the considered scenarios closely mimic the Sarcome-13 trial; consequently, additional simulations would be required to improve generalisability to all rare disease trials measuring time-to-event outcomes. As such, it would be worth evaluating the impact of the type of modelling considering historical data with more different shapes than those we considered in our hypothetical historical data. We also did not evaluate the impact on trial operating characteristics of different ratios of the number of historical and new controls. Since the impact of including individual historical control data is small even when this ratio is close to 3 (as in the settings considered in this paper i.e. 52 patients in the new trial versus 165 in the historical data), we can extrapolate to conclude that varying this ratio would have little impact on trial properties. Furthermore, we did not explore how changing the randomisation ratio in the new trial to recruit more patients to the experimental arm would impact performance. However, in the particular setting of the Sarcome-13 trial, investigators preferred to keep a 1:1 randomisation ratio which is generally what is decided in rare disease trials because high quality randomised data on the control is often scarce. We also only considered Weibull and three-parameter exponential models for analysing the new survival trial; other flexible survival models [47] were not investigated because we judged that they would to be too complex to apply to trials with small sample sizes. We do not consider our focus on Weibull and piecewise exponential models to be unnecessarily restrictive since they can accommodate a large variety of survival patterns. Even though we did not assess fundamentally different survival models with various values for baseline parameters, this was quite unlikely to occur from a clinical point of view. It should also be noted that while this simulation study was based on the Sarcome-13 trial, we considered a range of simulation scenarios to allow our conclusions to be informative for statisticians who are considering adopting a Bayesian approach for their own rare disease trial. We chose to use frequentist criteria (power, type I error) to express results. It is possible that this not fully reflect all the interest of the Bayesian approach. However, this choice was based on FDA recommendations on the presentation of Bayesian results in its guideline for the use of Bayesian statistics in medical device clinical trials [13]. Some other criteria such as the probability of success would maybe allow to better takes advantage of the Bayesian approach. It seems important to us that a trialist who wants to launch a new Bayesian study, using available historical data, evaluates the benefit-risk balance of combining these data with the current trial. Even if we are exposed to bias and increased type I error, using historical data is not only useful to gain in power but it also allows us to potentially gain a lot in precision and accuracy. This benefit-risk balance depends on the commensurability between historical and new data which can be evaluated both (i) from the beliefs of the trialist or from experts’ opinion, and (ii) from a simple statistical test comparing historical and new data. However, it is necessary to perform a preliminary large simulation study including various scenarios, which enables the trialist to evaluate the risks and possible benefits of the approach incorporating historical data, and to calibrate the weigh assigned to these data. When designing a trial, one must consider the time and resources needed to run such simulations. For a given scenario, running 5000 simulated trials for each of 28 configurations of the pair (*α*_0_, *ω*) given one scenario and analysing them by a Bayesian Weibull regression model took two hours on a server with 125 cores using R parallel programming (Parallel R package [48]).

To our knowledge, no previous published work has investigated the impact of incorporating both individual historical control data and aggregate treatment effect information when designing a randomised survival trial using a Weibull or a piecewise exponential survival regression model. One avenue for further research is to extend this approach to multiple sources of historical data. This will require taking into account the heterogeneity between studies. Ibrahim et al. [49] proposed a meta-analytic framework for incorporating aggregate (trial-level) historical data from the control or experimental or both into the analysis of a new survival trial when outcomes follow an exponential regression model. We could extend the method proposed by Ibrahim et al. to other survival models like Weibull or piecewise exponential models. Some authors proposed a “meta-experiment” approach based on a prospective meta-analysis design compared to a classical single randomised trial for challenging the sample size calculation [50]. However, this approach could be challenging in the context of rare diseases.

## Conclusions

In conclusion, the gains in power and accuracy possible by incorporating historical information commensurate with the new trial data has to be balanced against the risk of biased estimates and a possible loss in power if data are not commensurate. The weights allocated to the historical data have to be carefully chosen based on this trade-off.

## Additional files

Additional file 1: Motivating example: the Sarcome-13 trial. Context and design of the Sarcome-13 trial. This document also describes the available historical data. (PDF 296 kb)

Additional file 2: Likelihood of the distributions used. Description of the likelihood of a Weibull proportional hazards model and of a piecewise exponential model. (PDF 198 kb)

Additional file 3:**Figure A1.** Mixture prior distribution of the treatment effect for different values of *ω*. Mixture prior distribution where the informative component is ${\uppi}_{\mathrm{H}}\left(\upbeta |{D}_{TE}^H\right)\sim \mathrm{N}\left(\log (0.786),0.012\right)$ and the vague component is π_0_(β)~N(0, 10) (PDF 225 kb)

Additional file 4: R code of to compute the posterior joint distribution in the context of a Bayesian Weibull model (PDF 111 kb)

Additional file 5: **Figure A2.** Subgroup of OS2006 historical data (SARC-OS data). Observed Kaplan-Meier and parametric estimates (Weibull, 3-parameter exponential and Royston & Parmar flexible models) of the event-free survival curves for the subgroup of OS2006 patients satisfying the Sarcome-13 eligibility criteria (*n* = 165, 73 events). (PDF 113 kb)

Additional file 6: **Figure A3.** Historical datasets considered in the simulation study. Observed Kaplan-Meier curves of SARC-OS patients, that is, the subgroup of OS2006 patients satisfying the Sarcome-13 trial eligibility criteria, and Kaplan-Meier curves of the two hypothetical historical datasets: one simulated from a Weibull distribution and one from a piecewise exponential distribution. (PDF 115 kb)

Additional file 7: **Figure A4-A6.** Graphical representation of scenarios S1 to S32. Graphical representations of scenarios S1 to S12 (Fig. A4) where there is no conflict in terms of survival distribution and where a Weibull distribution is used for generating the historical control and new trial data; scenarios S13 to S24 (Fig. A5) where there is no conflict in terms of survival distribution and where a piecewise exponential survival distribution is used to generate the historical control and new trial data; scenarios S25 to S32 (Fig. A6) where there is a conflict in terms of survival distribution between the historical and new trial data. (PDF 655 kb)

Additional file 8:**Tables A1-A3.** Impact of α_0_ and ω on the operating characteristics for scenarios S1 to S12. These tables describe the impact of α_0_ and ω on the operating characteristics for scenarios S1 to S4 (Table A1), when historical and new control arm are commensurate, for scenarios S5 to S8 (Table A2), when there is a negative prior-data conflict, and for scenarios S9 to S12 (Table A3), when there is a positive prior-data conflict. A Weibull distribution is used to generate and analyse historical and new data, and ${\upsigma}_{\mathrm{H}}^2$ is equivalent to 329 events. (PDF 483 kb)

Additional file 9:**Tables A4-A6.** Impact of α_0_ and ω on the operating characteristics for scenarios S13 to S24. These tables describe the impact of α_0_ and ω on the operating characteristics for scenarios S13 to S16 (Table A4), when historical and new control arm are commensurate, for scenarios S17 to S20 (Table A5), when there is a negative prior-data conflict, and for scenarios S21 to S24 (Table A6), when there is a positive prior-data conflict. A piecewise exponential distribution with 3 pieces is used to generate and analyse historical and new data, and ${\upsigma}_{\mathrm{H}}^2$ is equivalent to 329 events. (PDF 483 kb)

Additional file 10:**Tables A7-A12.** Impact of α_0_ and ω on the operating characteristics for scenarios S1 to S12 when ${\upsigma}_{\mathrm{H}}^2$ is equivalent to 66 or 22 events. These tables describe the impact of α_0_ and ω on the operating characteristics for scenarios S1 to S4 (Tables A7 and A10), when historical and new control arm are commensurate, for scenarios S5 to S8 (Tables A8 and A11), when there is a negative prior-data conflict, and for scenarios S9 to S12 (Tables A9 and A12), when there is a positive prior-data conflict. A Weibull distribution is used to generate and analyse historical and new data, and ${\upsigma}_{\mathrm{H}}^2$ is equivalent to 66 (Tables A7-A9) or 22 (Tables A10-A12) events. (PDF 689 kb)

Additional file 11: **Figure A7.** Posterior distribution of the log-hazard ratio depending on the values of the weighting parameters. This figure represents no incorporation of historical data (solid black curve), weighted incorporation of historical data (dashed red curve), and full incorporation of historical data (dot dashed blue curve). (PDF 117 kb)

Additional file 12: **Tables A13-A16.** Impact of α_0_ and ω on the operating characteristics for scenarios S25 to S32 when there is a conflict in terms of the underlying survival distribution between historical and new data. These tables describe the impact of α_0_ and ω on the operating characteristics in case of a conflict in terms of survival distribution between historical and new data. Tables A13 and A14 describe results for scenarios S25 to S28 when the historical data follow a Weibull distribution, the new data follow a piecewise exponential distribution, and data are analysed using either a Bayesian Weibull model (Table A13) or a Bayesian piecewise exponential model (Table A14). Tables A15 and A16 describe results for scenarios S29 to S32 when the historical data follow a piecewise exponential distribution, the new data follow a Weibull distribution, and data are analysed using either a Bayesian piecewise exponential model (Table A15) or a Bayesian Weibull model (Table A16). (PDF 554 kb)

## Abbreviations

AIC: Akaike information criterion
EFS: Event-free survival
HCP: Historical controls sampled from a piecewise exponential model
HCW: Historical controls sampled from a Weibull distribution
HR: Hazard ratio
IPD: Individual patient data
MAP: Meta-analytic predictive prior
RMSE: Root mean square error
SD: Standard deviation
