# Supplementary material for: Incorporating individual historical controls and aggregate treatment effect estimates into a Bayesian survival trial: a simulation study
Source: BMC Med Res Methodol. 2019 Apr 24;19:85. doi: 10.1186/s12874-019-0714-z (PMC6480797; doi:10.1186/s12874-019-0714-z)
Supplement: Supplementary file 1 — Motivating example: the Sarcome-13 trial. Context and design of the Sarcome-13 trial. This document also describes the available historical data. (PDF 296 kb) [file 12874_2019_714_MOESM1_ESM.pdf]

Motivating example: the Sarcome-13 trial

Osteosarcoma, a malignant tumor of the bones, is a rare disease representing less than 10% of all cancers in adolescents and young adults and accounts for around 0.2-3 incident cases per million per year in Europe. Patients with metastases or poor histological response to neoadjuvant chemotherapy have a persistently dismal prognosis.

Regulatory history

The INT-0133 randomised phase III trial evaluated, using a factorial design, the benefit associated with both the addition of mifamurtide and the addition of ifosfamide to standard chemotherapy for the treatment of osteosarcoma in patients younger than 31 years. The analysis was performed considering two strata: localised [1, 2] and metastatic [3] osteosarcoma. The effect of mifamurtide on overall survival, as well as on event-free survival (EFS), was homogeneous across both strata even if the benefit of mifamurtide for overall survival was significant in localised osteosarcoma but not significant in metastatic osteosarcoma. Furthermore, a possible interaction between the addition of ifosfamide and mifamurtide, reported by Meyer et al. in the first publication [1], complicated the interpretation of subsequent findings [2, 3].

Based on these results, the European Medicines Agency granted mifamurtide a centralised marketing authorisation. However, this European approval does not cover patients with primary metastatic disease. In addition, no approval has been granted in the United States, and the drug is not reimbursed in France. The French Transparency Commission required additional investigations before it would approve and reimburse mifamurtide for standard use in first line therapy of osteosarcoma.

Planned Sarcome-13 trial

Against this backdrop, a new randomised controlled phase II trial (Sarcome-13) will be conducted in France evaluating mifamurtide in patients with newly diagnosed high-risk osteosarcoma (metastatic and poor histological response). The aim of this trial is to evaluate the benefit in terms of EFS of mifamurtide in combination with post-operative chemotherapy compared with chemotherapy alone (randomisation

1:1) in patients less than 31 years old. Due to the rare disease setting, we relaxed the alpha level of the one-sided log-rank test to a significance level of 10% for the standard frequentist approach [4, 5], and a pragmatic recruitment target has been set of accruing 105 patients over 3 years (with 2 years of follow-up for the last patient). If this target is met, the power is 80% if the true hazard ratio (HR) is 0.55 (based on 43 events), whereas it decreases to 33% and 20% for a 0.786 (48 events) and 0.886 (50 events), respectively. The trial will be funded by the French Ministry of Health (Programme Hospitalier de Recherche Clinique, PHRC-K 16-130) and supported by Takeda. The primary analysis of the Sarcome-13 trial will be frequentist, based on fitting a Cox proportional hazards model. Through a Bayesian analysis, we seek to augment data from the Sarcome-13 study with relevant historical information to increase the trial's power to reliably detect smaller, but more plausible, effects. In the Bayesian analysis, post-operative chemotherapy plus mifamurtide will be deemed superior to post-operative chemotherapy alone if the posterior probability of a HR lower than one exceeds 0.9.

Individual historical data

The first source of available historical information is individual patient data on patients with high-risk osteosarcoma from the OS2006 trial (NCT00470223) [6]. This trial included 318 patients and used the same backbone chemotherapy as will be used in the Sarcome-13 trial. Selecting from OS2006 all patients who fulfilled the planned Sarcome-13 eligibility criteria, referred as to SARC-OS thereafter, we identified EFS data on 165 patients (73 events) who were under the age of 31 at diagnosis, with metastases or poor histological response to neoadjuvant chemotherapy, who did not experience an event during pre-operative chemotherapy, and who underwent surgery of the primary tumour. In order to match with the Sarcome-13 duration, we truncated data at 5 years. The Kaplan-Meier curve for these patients is presented in Figure S1.

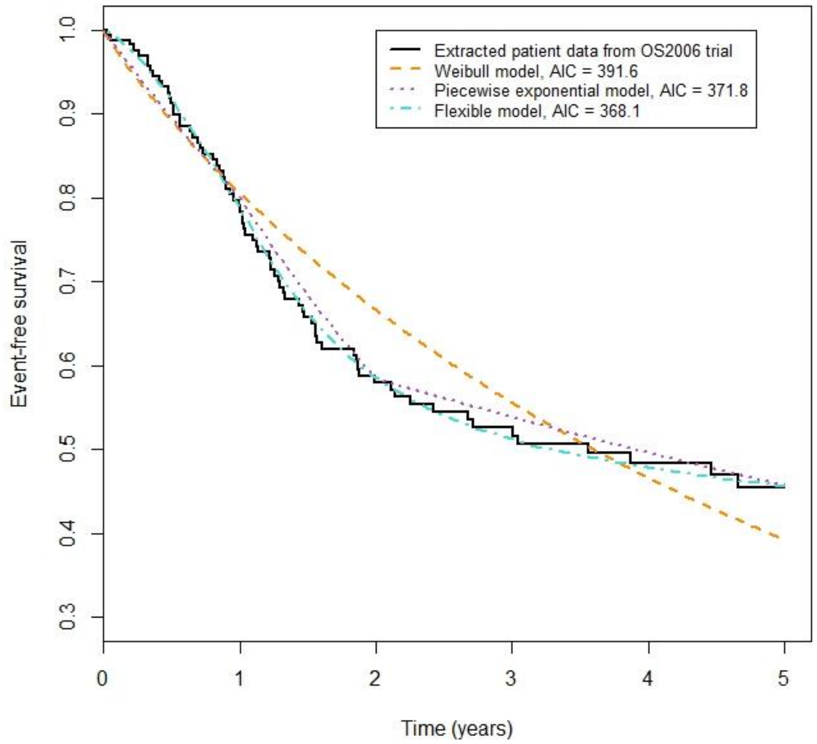

Figure S1: Subgroup of OS2006 historical data (SARC-OS data)

Observed Kaplan-Meier and parametric estimates (Weibull, 3-parameter exponential and Royston & Parmar flexible models) of the event-free survival curves for the subgroup of OS2006 patients satisfying the Sarcome-13 eligibility criteria (n = 165, 73 events).

We investigated which parametric survival regression model best fits these data. Restricting attention to models consistent with an assumption of proportional hazards, we compared the fit of a Weibull model with that of 3-parameter piecewise exponential models with various time partitions. A flexible Royston & Parmar model [7] with three degrees of freedom was also fitted and taken to be the benchmark model. Based on the Akaike information criterion [8] (AIC), we selected the piecewise exponential model with partitions set at [0,1), [1,2) and [2,+∞) years with AIC = 371.8 as closest to the benchmark model (AIC = 368.1); the Weibull model has AIC = 391.6. We notice that the selected piecewise exponential model fits poorly at short follow-up times. However, changing time partitions to have a shorter interval for the first partition did not significantly increase goodness-of-fit (data not shown) and furthermore has no clinical justification.

Aggregate historical data

The second source of available historical information comprises the two estimates of the relative treatment effect on EFS of post-operative chemotherapy plus mifamurtide versus post-operative chemotherapy alone which were reported by the INT-0133 trial (one for localised osteosarcoma [2] and one for metastatic osteosarcoma [3]). Given the published HRs obtained from fitting a Cox proportional hazard model in patients with localised osteosarcoma [2] (HR = 0.80; 95%CI, 0.62-1.00; n = 662) and in patients with metastatic osteosarcoma [3] (HR = 0.72; 95%CI, 0.42-1.20; n = 91), we concluded the absence of significant heterogeneity of the treatment effect across the two strata ($p = 0.72$, $i^2 = 0$).

Therefore, a fixed effect meta-analysis with inverse variance weighting was used to obtain an overall estimate of the treatment effect (HR = 0.786; 95%CI, 0.63-0.98). The corresponding estimates of the log hazard ratio ($\widehat{\beta_H}$), which is the parameter that we will consider thereafter, and its variance (s^2) are -0.241 and 0.012, respectively. This variance is approximately equivalent to what would be obtained if the estimate was based on 329 events (Schoenfeld formula [9]).

References

1. Meyers PA, Schwartz CL, Krailo M, Kleinerman ES, Betcher D, Bernstein ML, et al. Osteosarcoma: a randomized, prospective trial of the addition of ifosfamide and/or muramyl tripeptide to cisplatin, doxorubicin, and high-dose methotrexate. J Clin Oncol. 2005;23:2004–11.

2. Meyers PA, Schwartz CL, Krailo MD, Healey JH, Bernstein ML, Betcher D, et al. Osteosarcoma: the addition of muramyl tripeptide to chemotherapy improves overall survival--a report from the Children's Oncology Group. J Clin Oncol. 2008;26:633–8.

3. Chou AJ, Kleinerman ES, Krailo MD, Chen Z, Betcher DL, Healey JH, et al. Addition of muramyl tripeptide to chemotherapy for patients with newly diagnosed metastatic osteosarcoma: a report from the Children's Oncology Group. Cancer. 2009;115:5339–48.

4. Bogaerts J, Sydes MR, Keat N, McConnell A, Benson A, Ho A, et al. Clinical trial designs for rare diseases: studies developed and discussed by the International Rare Cancers Initiative. Eur J Cancer Oxf Engl 1990. 2015;51:271–81.

5. Parmar MKB, Sydes MR, Morris TP. How do you design randomised trials for smaller populations? A framework. BMC Med. 2016;14:183.

6. Piperno-Neumann S, Deley M-CL, Rédini F, Pacquement H, Marec-Bérard P, Petit P, et al. Zoledronate in combination with chemotherapy and surgery to treat osteosarcoma (OS2006): a randomised, multicentre, open-label, phase 3 trial. Lancet Oncol. 2016;17:1070–80.

7. Royston P, Parmar MKB. Flexible parametric proportional-hazards and proportional-odds models for censored survival data, with application to prognostic modelling and estimation of treatment effects. Stat Med. 2002;21:2175–97.

8. Akaike H. A new look at the statistical model identification. IEEE Trans Autom Control. 1974;19:716–23.

9. Schoenfeld DA. Sample-size formula for the proportional-hazards regression model. Biometrics. 1983;39:499–503.
