# Supplementary material for: Incorporating individual historical controls and aggregate treatment effect estimates into a Bayesian survival trial: a simulation study
Source: BMC Med Res Methodol. 2019 Apr 24;19:85. doi: 10.1186/s12874-019-0714-z (PMC6480797; doi:10.1186/s12874-019-0714-z)
Supplement: Supplementary file 2 — Likelihood of the distributions used. Description of the likelihood of a Weibull proportional hazards model and of a piecewise exponential model. (PDF 198 kb) [file 12874_2019_714_MOESM2_ESM.pdf]

Likelihood of the distributions used

The likelihood of a Weibull proportional hazards model for the *n* trial subjects is written as

$$L(\beta_0, \gamma|D) = \prod_{i=1}^{n} \left[\exp\{\beta_0\} \frac{1}{\gamma} t_i^{\frac{1}{\gamma}-1} \right]^{\nu_i} exp\left\{ -exp\{\beta_0\} t_i^{\frac{1}{\gamma}} \right\}$$

where γ is the scale parameter, β_0 is the intercept, $t = (t_1, t_2, \dots, t_n)$ represents survival times, and where $\nu_i = 1$ if the ith subject failed, and 0 if t_i did not fail.

For a piecewise exponential model, where the time axis is divided in J intervals $(0,\ s_1], (s_1, s_2], \dots, (s_{J-1}, s_J]$ and where we assume constant baseline hazard (λ_j) for $t_i \in\ I_j = (s_{j-1}, s_j]$, the hazard function is as follows

$$h_i(t) = \ \lambda_j \times \exp(\beta \times trt) \qquad for\ s_{j-1} < t < s_j$$

The likelihood of a piecewise model for the *n* trial subjects is written as

$$L(\lambda|D) = \prod_{i=1}^{n} \prod_{j=1}^{J} (\lambda_j)^{\delta_{ij}\nu_i} exp\left\{ -\delta_{ij} \left[\lambda_j(t_i - s_{j-1}) + \sum_{g=1}^{j-1} \lambda_g(s_g - s_{g-1}) \right] \right\}$$

Letting λ_j with $j = 1, \dots, J$ be the baseline hazard in each interval, and $\delta_i = 1$ of the ith subject failed or was censored in the jth interval, and 0 otherwise.
