# Supplementary material for: Incorporating individual historical controls and aggregate treatment effect estimates into a Bayesian survival trial: a simulation study
Source: BMC Med Res Methodol. 2019 Apr 24;19:85. doi: 10.1186/s12874-019-0714-z (PMC6480797; doi:10.1186/s12874-019-0714-z)
Supplement: Supplementary file 3 — Figure A1. Mixture prior distribution of the treatment effect for different values of ω. Mixture prior distribution where the informative component is ${\uppi}_{\mathrm{H}}\left(\upbeta |{D}_{TE}^H\right)\sim \mathrm{N}\left(\log (0.786),0.012\right)$πHβDTEH~Nlog0.7860.012 and the vague component is π0(β)~N(0, 10) (PDF 225 kb) [file 12874_2019_714_MOESM3_ESM.pdf]

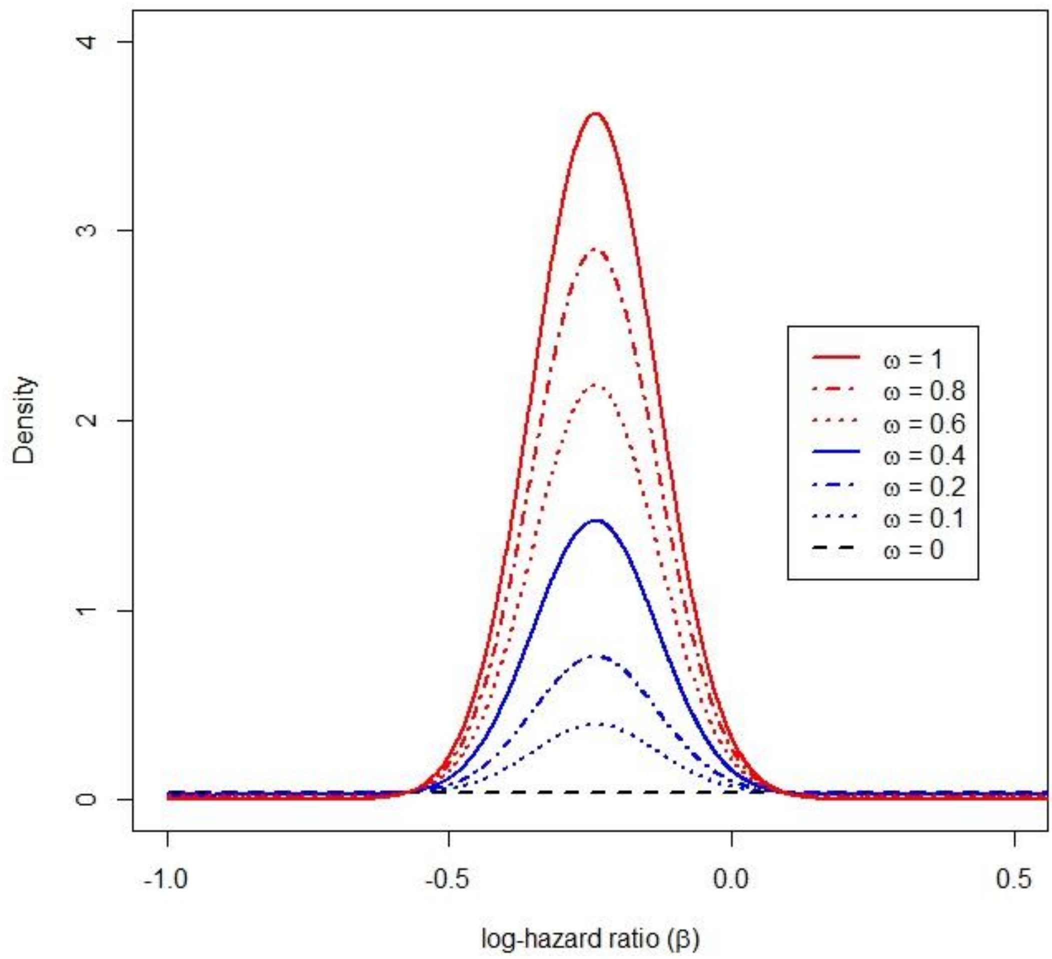

Figure A2: Mixture prior distribution of the treatment effect for different values of $\boldsymbol{\omega}$

Mixture prior distribution where the informative component is $\pi_H(\beta|D_{TE}^H) \sim N(\log(0.786), 0.012)$ and the vague component is $\pi_0(\beta) \sim N(0, 10)$
