# Supplementary material for: Incorporating individual historical controls and aggregate treatment effect estimates into a Bayesian survival trial: a simulation study
Source: BMC Med Res Methodol. 2019 Apr 24;19:85. doi: 10.1186/s12874-019-0714-z (PMC6480797; doi:10.1186/s12874-019-0714-z)
Supplement: Supplementary file 4 — R code of to compute the posterior joint distribution in the context of a Bayesian Weibull model (PDF 111 kb) [file 12874_2019_714_MOESM4_ESM.pdf]

R code used to compute the joint posterior distribution in the context of a Weibull model

```
########################################
# Data of the new trial

var_lik = cbind(t, status, trt, type)

#######################################
# Frequentist Weibull analysis

f_w_s    <- survreg(Surv(data$t, data$status)~data$trt, data = data, dist = "weibull")

beta1   = data.frame(summary(f_w_s)$coefficients)[2,1]
beta0   = data.frame(summary(f_w_s)$coefficients)[1,1]
scale   = f_w_s$scale

theta = c(beta0, scale, beta1)

#####################################
# Historical individual control data

hist_control <- subset(hist_control, select = c(t, status, trt, type))

#####################################
# Pool of historical indiviual control data and new data

ind_data = rbind(var_lik, hist_control)

var_ind_data = cbind(ind_data$t, ind_data$status, ind_data$trt, ind_data$type)

data_all  <- matrix(data = var_ind_data, nrow = dim(var_ind_data)[1], ncol = 4)

#####################################
# Bayesian Weibull analysis
# theta = vector of parameters
# data = pool of historical and new individual data
# hr_hist = aggregate historical relative treatment effect
# sd_hist = standard deviation of the historical relative treatment effect
# hr_limit = threshold for the decision rule
# prob_limit = Probability of posterior distribution ( P(HR_new < hr_limit) > prob_limit) )
# nmcmc = number of mcmc
# burn_in = number of burn_in
######################################

bay_weibull <- function(theta, data, hr_hist, sd_hist, w, a0, hr_limit, prob_limit, nmcmc, burn_in)
{
  i <- 0
  j <- 0

  pool    <- matrix(data = NA, nrow = length(theta) + 4, ncol = length(w))
```

```
  bw_beta1 <- matrix(data = NA, nrow = length(w), ncol = length(a0))

  bw_beta1_sd  <- matrix(data = NA, nrow = length(w), ncol = length(a0))
  bw_beta1_inf <- matrix(data = NA, nrow = length(w), ncol = length(a0))
  bw_beta1_sup <- matrix(data = NA, nrow = length(w), ncol = length(a0))

  bw_beta0    <- matrix(data = NA, nrow = length(w), ncol = length(a0))
  bw_scale    <- matrix(data = NA, nrow = length(w), ncol = length(a0))

  proportion <- matrix(data = NA, nrow = length(w), ncol = length(a0))

  for (i in(1:length(a0)))
  {
   for (j in (1:length(w)))
   {
    logpost <- function(theta, data)
    {
     beta0 = theta[1]
     scale = theta[2]
     beta1 = theta[3]

     t     = data[,1]
     c     = data[,2]
     trt   = data[,3]

     logf = function(t, c, trt, beta0, scale, beta1, a0)
     {
      lambda = -(beta0 + beta1*trt) * (1/scale)

      llike  = ifelse(data[,4] == 1, c * (log(1/scale) + ((1/scale)-1) * log(t) + lambda) - exp(lambda) *
(t^(1/scale)),
                      a0[i]*(c * (log(1/scale) + ((1/scale)-1) * log(t) + lambda) - exp(lambda) * (t^(1/scale))) )
      return(llike)
     }

     scale_p = dgamma(scale, shape = 0.0001, scale = 10000, log = TRUE)
     beta0_p = dnorm(beta0, 0, sqrt(10000), log = TRUE)
     mixture = w[j] * dnorm(-beta1/scale, log(hr_hist), sd_hist) + (1 - w[j]) * dnorm(-beta1/scale, 0,
sqrt(10))

     v_prior  = beta0_p + log(-mixture/scale_p) + scale_p

     val_post = v_prior + sum(logf(t, c, trt, beta0, scale, beta1, a0))

     return(val_post)
    }

    start      = c(beta0, scale, -beta1/scale)

    laplace = laplace(logpost, start, data)
```

```
 proposal    = list(var = laplace$var, scale = 1)

 s = rwmetrop(logpost, proposal, start, nmcmc, data)
 dimnames(s$par)[[2]] = c("beta0", "scale", "beta1")

 mcmc = mcmc(s$par[-c(1:burn_in),])

 diag = geweke.diag(mcmc)

 if ((1-pnorm(diag$z[1]) <= 0.05)) stop("Convergence issue")
 if ((1-pnorm(diag$z[2]) <= 0.05)) stop("Convergence issue")
 if ((1-pnorm(diag$z[3]) <= 0.05)) stop("Convergence issue")

 cpt <- function(beta, scale)
 {
   n   = length(beta)
   res = numeric(n)

   fun <- function(i, beta, scale, n) res <- ifelse(exp(-beta/scale) < hr_limit, 1, 0)

   res = sapply(X = 1, FUN = fun, beta = beta, scale = scale, n = n)
   z   = sum(res)/length(beta)

   return(z)
 }

 prop = cpt(beta = mcmc[,3], scale = mcmc[,2])

 post_mean =   apply(mcmc, 2, mean)
 b_beta0   =   post_mean[1]
 b_scale   =   post_mean[2]
 b_beta1   =  -post_mean[3] / b_scale

 post_sds     = apply(mcmc, 2, sd)
 b_beta1_sd   = post_sds[3] / b_scale

 cred_int  = apply(mcmc, 2, quantile, c(prob_limit, 1-prob_limit))
 beta_CI   = -cred_int[,3]/b_scale

 pool[,j] = c(b_beta0, b_scale, b_beta1, b_beta1_sd, beta_CI, prop)

 j <- j + 1
}

bw_beta0[,i]     = pool[1,]
bw_scale[,i]     = pool[2,]
bw_beta1[,i]     = pool[3,]
bw_beta1_sd[,i]  = pool[4,]
bw_beta1_sup[,i] = pool[5,]
bw_beta1_inf[,i] = pool[6,]
proportion[,i]   = pool[7,]
```

```
    i <- i + 1
  }

  return(list(beta1 = bw_beta1, beta1_sd = bw_beta1_sd, beta1_inf = bw_beta1_inf, beta1_sup =
bw_beta1_sup, beta0 = bw_beta0, scale = bw_scale, prop = proportion))
  }

bay_res = bay_weibull(theta = theta, data = data_all, hr_hist = 0.786, sd_hist = 0.1103,
                      w = c(0), a0 = c(0), hr_limit = 1, prob_limit = 0.10, nmcmc = 25000, burn_in = 5000)
```
