# Supplementary material for: Incorporating individual historical controls and aggregate treatment effect estimates into a Bayesian survival trial: a simulation study
Source: BMC Med Res Methodol. 2019 Apr 24;19:85. doi: 10.1186/s12874-019-0714-z (PMC6480797; doi:10.1186/s12874-019-0714-z)
Supplement: Supplementary file 5 — Figure A2. Subgroup of OS2006 historical data (SARC-OS data). Observed Kaplan-Meier and parametric estimates (Weibull, 3-parameter exponential and Royston & Parmar flexible models) of the event-free survival curves for the subgroup of OS2006 patients satisfying the Sarcome-13 eligibility criteria (n = 165, 73 events). (PDF 113 kb) [file 12874_2019_714_MOESM5_ESM.pdf]

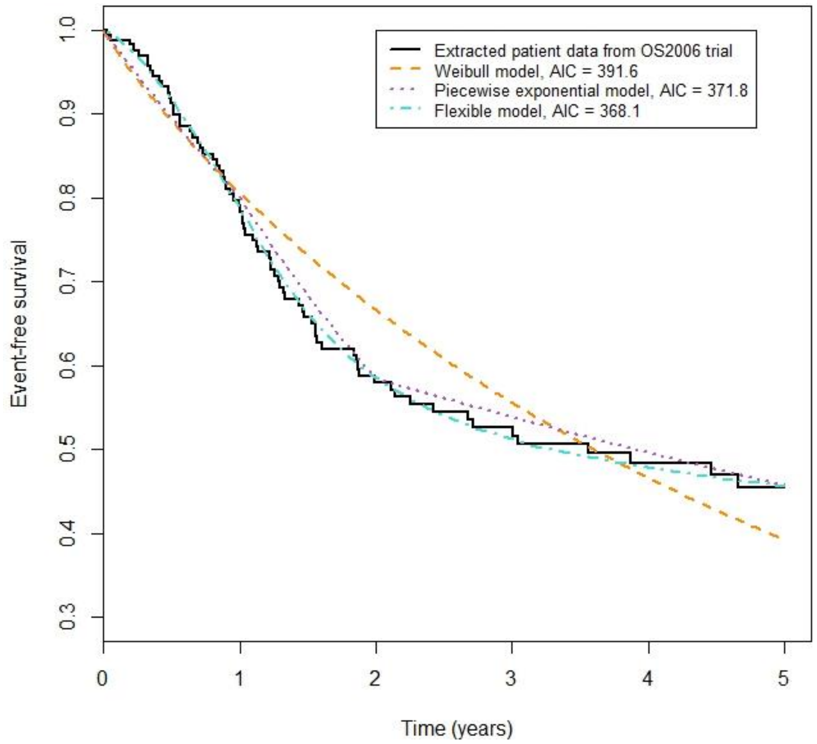

Figure A2: Subgroup of OS2006 historical data (SARC-OS data)

Observed Kaplan-Meier and parametric estimates (Weibull, 3-parameter exponential and Royston & Parmar flexible models) of the event-free survival curves for the subgroup of OS2006 patients satisfying the Sarcome-13 eligibility criteria (n = 165, 73 events).
