# Supplementary material for: Incorporating individual historical controls and aggregate treatment effect estimates into a Bayesian survival trial: a simulation study
Source: BMC Med Res Methodol. 2019 Apr 24;19:85. doi: 10.1186/s12874-019-0714-z (PMC6480797; doi:10.1186/s12874-019-0714-z)
Supplement: Supplementary file 6 — Figure A3. Historical datasets considered in the simulation study. Observed Kaplan-Meier curves of SARC-OS patients, that is, the subgroup of OS2006 patients satisfying the Sarcome-13 trial eligibility criteria, and Kaplan-Meier curves of the two hypothetical historical datasets: one simulated from a Weibull distribution and one from a piecewise exponential distribution. (PDF 115 kb) [file 12874_2019_714_MOESM6_ESM.pdf]

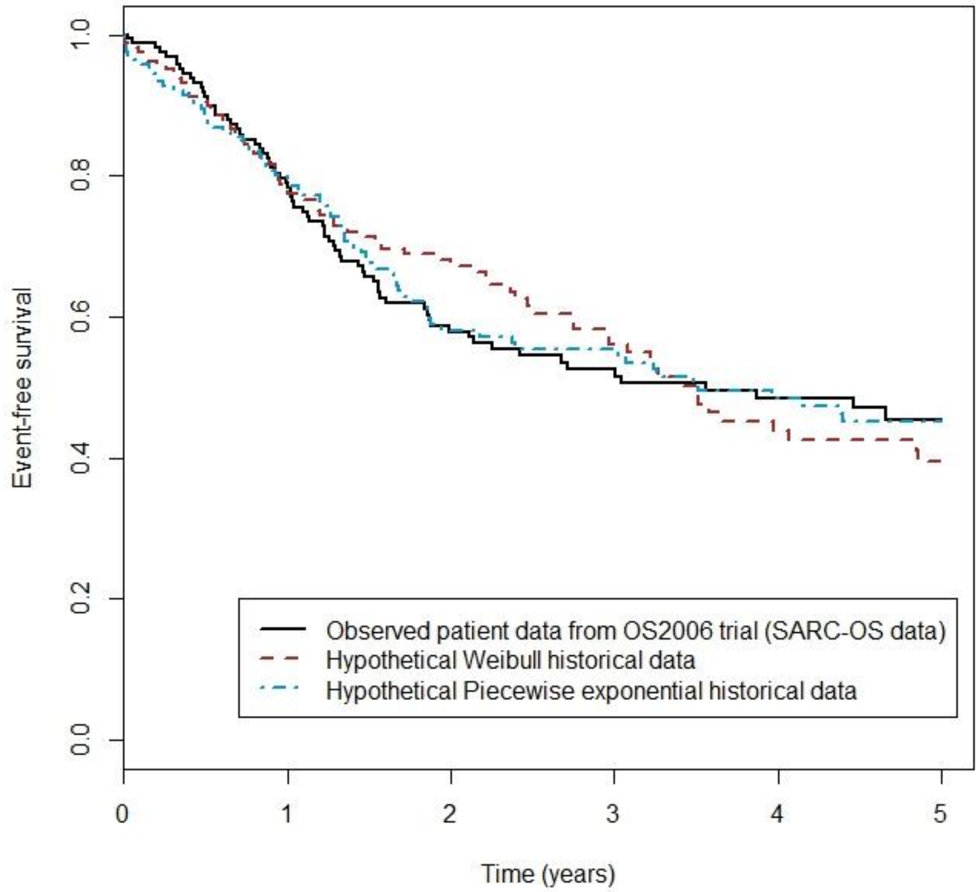

Figure A3: Historical datasets considered in the simulation study

Observed Kaplan-Meier curves of SARC-OS patients, that is, the subgroup of OS2006 patients satisfying the Sarcome-13 trial eligibility criteria (solid black curve), and Kaplan-Meier curves of the two hypothetical historical datasets: one simulated from a Weibull distribution (dashed brown curve) and one from a piecewise exponential distribution (dot dashed blue curve)
