# Supplementary material for: Incorporating individual historical controls and aggregate treatment effect estimates into a Bayesian survival trial: a simulation study
Source: BMC Med Res Methodol. 2019 Apr 24;19:85. doi: 10.1186/s12874-019-0714-z (PMC6480797; doi:10.1186/s12874-019-0714-z)

Figure A4: Graphical representation of scenarios S1 to S12: no conflict in terms of survival distributions - Weibull survival distribution for the historical control and the new trial data. Black curves represent the Kaplan-Meier EFS of hypothetical historical control survival data, colored curves represent EFS distribution in the control (plain line) and experimental (dashed line) arm of the new trial

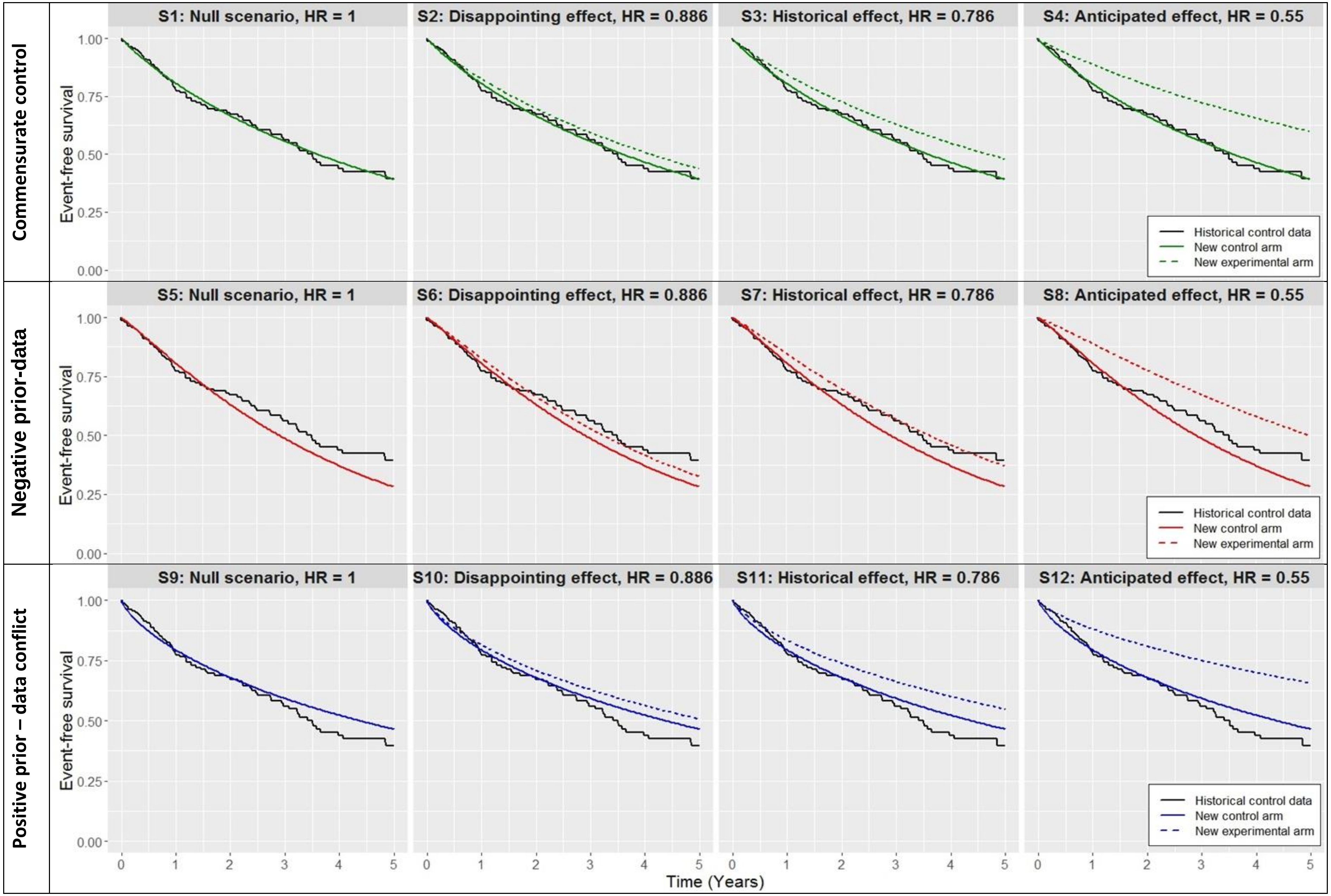

Figure A5: Graphical representations of scenarios S13 to S24: no conflict in terms of survival distribution - Piecewise exponential survival distribution for the historical control and the new trial data. Black curves represent the Kaplan-Meier EFS of hypothetical historical control survival data, colored curves represent EFS distribution in the control (plain line) and experimental (dashed line) arm of the new trial

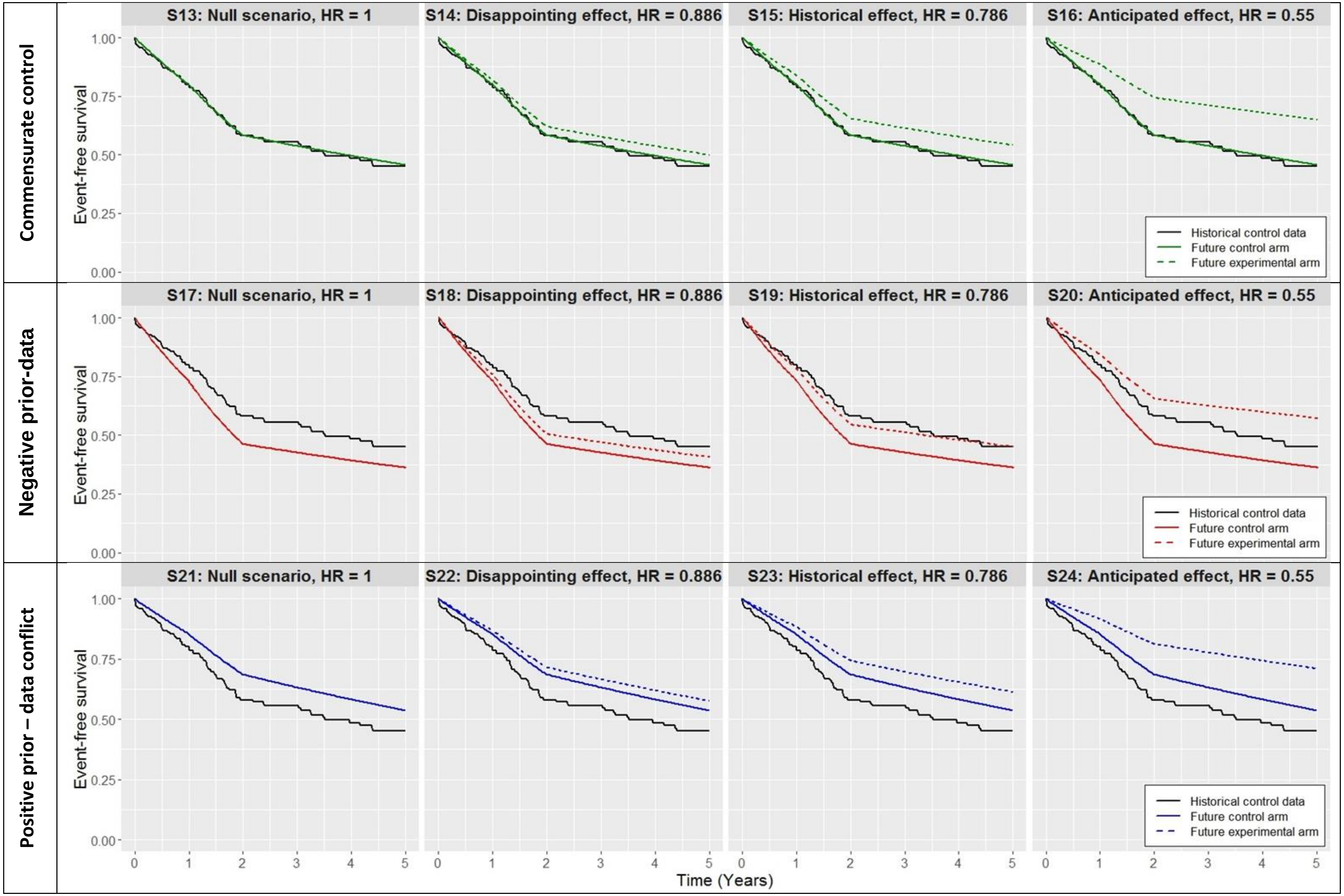

Figure A6: Graphical representations of scenarios S25 to S32: conflict in terms of survival distribution between historical and new trial data. Black curves represent the Kaplan-Meier EFS of hypothetical historical control survival data, colored curves represent EFS distribution in the control (plain line) and experimental (dashed line) arm of the new trial

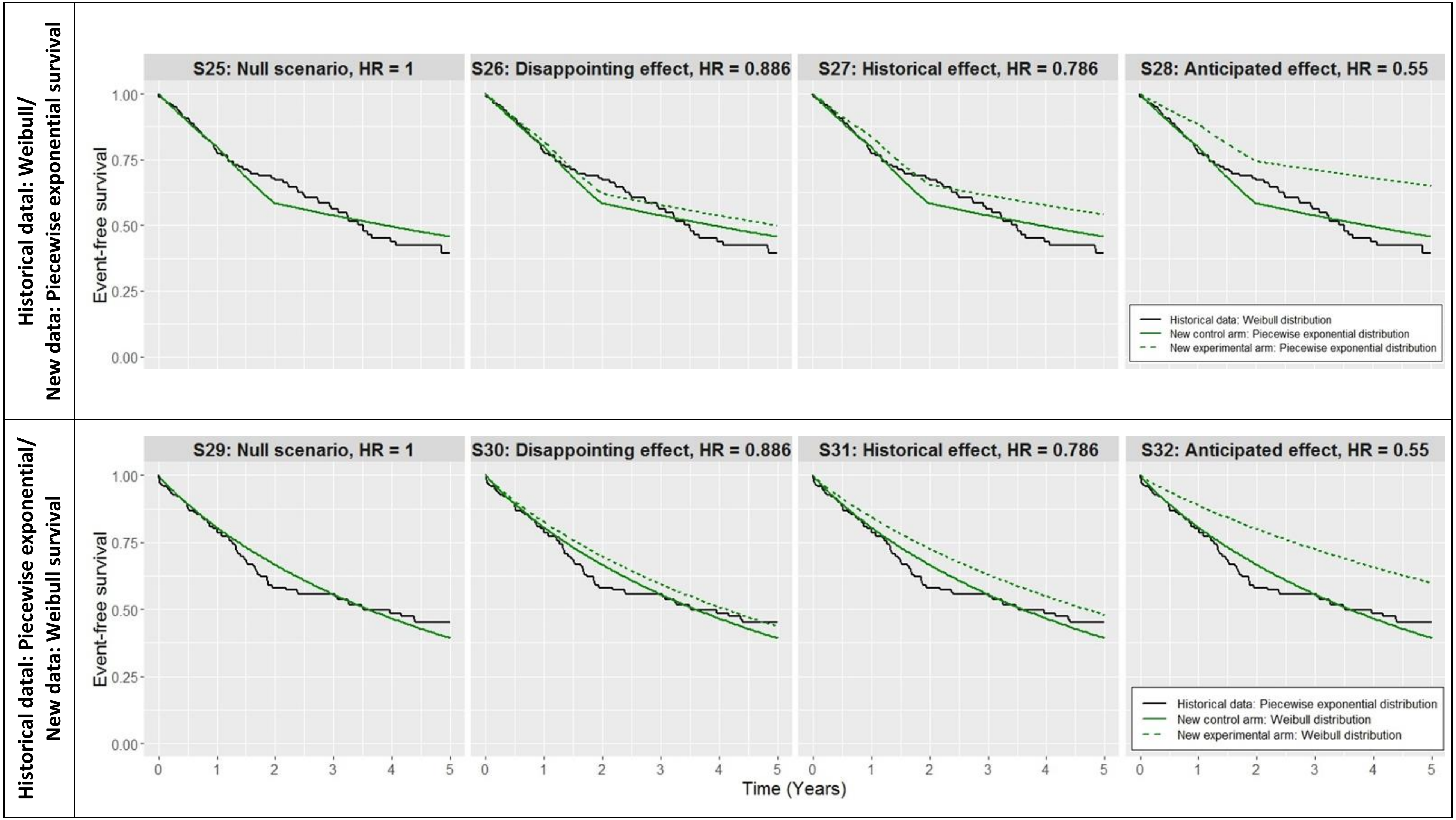

Supplement: Supplementary file 7 — Figure A4-A6. Graphical representation of scenarios S1 to S32. Graphical representations of scenarios S1 to S12 (Fig. A4) where there is no conflict in terms of survival distribution and where a Weibull distribution is used for generating the historical control and new trial data; scenarios S13 to S24 (Fig. A5) where there is no conflict in terms of survival distribution and where a piecewise exponential survival distribution is used to generate the historical control and new trial data; scenarios S25 to S32 (Fig. A6) where there is a conflict in terms of survival distribution between the historical and new trial data. (PDF 655 kb) [file 12874_2019_714_MOESM7_ESM.pdf]
