# Supplementary material for: Incorporating individual historical controls and aggregate treatment effect estimates into a Bayesian survival trial: a simulation study
Source: BMC Med Res Methodol. 2019 Apr 24;19:85. doi: 10.1186/s12874-019-0714-z (PMC6480797; doi:10.1186/s12874-019-0714-z)
Supplement: Supplementary file 8 — Tables A1-A3. Impact of α0 and ω on the operating characteristics for scenarios S1 to S12. These tables describe the impact of α0 and ω on the operating characteristics for scenarios S1 to S4 (Table A1), when historical and new control arm are commensurate, for scenarios S5 to S8 (Table A2), when there is a negative prior-data conflict, and for scenarios S9 to S12 (Table A3), when there is a positive prior-data conflict. A Weibull distribution is used to generate and analyse historical and new data, and ${\upsigma}_{\mathrm{H}}^2$σH2 is equivalent to 329 events. (PDF 483 kb) [file 12874_2019_714_MOESM8_ESM.pdf]

Table A1. Impact of α_0 and ω on the operating characteristics and the estimated performance for scenarios S1 to S4 (Weibull distribution for historical and new data, commensurate control arm), with σ_H^2 equivalent to 329 events.

Commensurate control arm																	
Treatment effect / Parameters		S1: Null scenario HR = 1				S2: Disappointing effect scenario HR = 0.886				S3: Historical effect scenario HR = 0.786				S4: Anticipated effect scenario HR = 0.55			
α_0	ω	Bias	SD	RMSE	Alpha	Bias	SD	RMSE	Power	Bias	SD	RMSE	Power	Bias	SD	RMSE	Power
0	0	0.0024	0.263	0.262	0.100	0.0015	0.268	0.267	0.208	0.0022	0.275	0.275	0.359	-0.0075	0.304	0.298	0.794
	0.1	-0.0490	0.240	0.234	0.216	-0.0275	0.234	0.213	0.368	0.0014	0.236	0.210	0.545	0.0582	0.288	0.289	0.900
	0.2	-0.0794	0.222	0.223	0.323	-0.0439	0.210	0.185	0.491	0.0008	0.210	0.176	0.655	0.0999	0.272	0.289	0.944
	0.4	-0.1189	0.195	0.213	0.499	-0.0645	0.178	0.155	0.668	0.0007	0.176	0.132	0.808	0.1573	0.244	0.292	0.983
	0.6	-0.1469	0.171	0.210	0.641	-0.0782	0.155	0.136	0.796	0.0007	0.152	0.102	0.893	0.2015	0.217	0.298	0.993
	0.8	-0.1718	0.147	0.208	0.780	-0.0894	0.133	0.121	0.897	0.0007	0.131	0.074	0.958	0.2432	0.185	0.304	0.997
	1	-0.2035	0.105	0.207	0.969	-0.1017	0.106	0.109	0.989	0.0006	0.107	0.039	0.997	0.3109	0.110	0.313	1
0.3	0	-0.0058	0.232	0.214	0.084	-0.0071	0.238	0.220	0.211	-0.0070	0.245	0.231	0.396	-0.0158	0.274	0.259	0.883
	0.1	-0.0598	0.213	0.196	0.204	-0.0370	0.205	0.171	0.395	-0.0044	0.206	0.170	0.613	0.0542	0.263	0.260	0.957
	0.2	-0.0900	0.197	0.191	0.319	-0.0526	0.183	0.150	0.528	-0.0029	0.183	0.139	0.721	0.0971	0.251	0.264	0.981
	0.4	-0.1268	0.174	0.190	0.499	-0.0705	0.156	0.128	0.706	-0.0014	0.154	0.103	0.859	0.1552	0.227	0.274	0.995
	0.6	-0.1519	0.153	0.192	0.643	-0.0815	0.136	0.116	0.828	-0.0009	0.134	0.078	0.929	0.1990	0.203	0.283	0.999
	0.8	-0.1726	0.133	0.195	0.784	-0.0899	0.121	0.109	0.917	-0.0007	0.119	0.058	0.969	0.2402	0.174	0.292	1
	1	-0.1959	0.102	0.200	0.954	-0.0977	0.103	0.105	0.987	-0.0006	0.103	0.039	0.997	0.3023	0.105	0.305	1
0.6	0	-0.0095	0.219	0.200	0.082	-0.0108	0.225	0.206	0.222	-0.0111	0.233	0.218	0.418	-0.0199	0.261	0.248	0.911
	0.1	-0.0629	0.202	0.186	0.210	-0.0401	0.193	0.161	0.408	-0.0066	0.195	0.159	0.625	0.0501	0.253	0.252	0.973
	0.2	-0.0920	0.187	0.184	0.321	-0.0549	0.173	0.141	0.547	-0.0046	0.173	0.130	0.752	0.0925	0.242	0.258	0.988
	0.4	-0.1275	0.165	0.185	0.500	-0.0715	0.147	0.122	0.725	-0.0023	0.146	0.096	0.877	0.1500	0.221	0.268	0.997
	0.6	-0.1512	0.147	0.188	0.644	-0.0815	0.130	0.113	0.840	-0.0012	0.129	0.074	0.938	0.1935	0.198	0.278	0.999
	0.8	-0.1707	0.129	0.191	0.785	-0.0891	0.116	0.107	0.921	-0.0008	0.115	0.056	0.971	0.2344	0.171	0.287	1
	1	-0.1920	0.101	0.196	0.944	-0.0959	0.101	0.104	0.985	-0.0005	0.102	0.040	0.997	0.2973	0.103	0.300	1
1	0	-0.0121	0.210	0.193	0.087	-0.0137	0.216	0.200	0.233	-0.0141	0.223	0.212	0.448	-0.0230	0.252	0.242	0.929
	0.1	-0.0640	0.194	0.182	0.215	-0.0418	0.185	0.156	0.425	-0.0086	0.187	0.154	0.653	0.0459	0.246	0.249	0.980
	0.2	-0.0923	0.180	0.180	0.325	-0.0558	0.166	0.138	0.559	-0.0059	0.166	0.126	0.771	0.0878	0.237	0.255	0.992
	0.4	-0.1271	0.160	0.182	0.501	-0.0719	0.142	0.121	0.733	-0.0030	0.141	0.094	0.884	0.1443	0.217	0.266	0.998
	0.6	-0.1497	0.143	0.185	0.641	-0.0812	0.126	0.111	0.841	-0.0012	0.125	0.072	0.939	0.1874	0.196	0.274	0.999
	0.8	-0.1685	0.126	0.189	0.776	-0.0883	0.114	0.106	0.920	-0.0009	0.113	0.056	0.973	0.2285	0.169	0.283	1
	1	-0.1892	0.099	0.194	0.933	-0.0945	0.100	0.103	0.983	-0.0006	0.100	0.042	0.997	0.2931	0.101	0.296	1

Table A2. Impact of α_0 and ω on the operating characteristics for scenarios S5 to S8 (Weibull distribution for historical and new data, negative prior-data conflict), with σ_H^2 equivalent to 329 events.

Control arm: negative prior-data conflict																	
Treatment effect / Parameters		S5: Null scenario HR = 1				S6: Disappointing effect scenario HR = 0.886				S7: Historical effect scenario HR = 0.786				S8: Anticipated effect scenario HR = 0.55			
α_0	ω	Bias	SD	RMSE	Alpha	Bias	SD	RMSE	Power	Bias	SD	RMSE	Power	Bias	SD	RMSE	Power
0	0	0.0003	0.241	0.243	0.104	0.0008	0.240	0.243	0.224	0.0036	0.245	0.247	0.382	-0.0024	0.263	0.264	0.849
	0.1	-0.0494	0.221	0.222	0.215	-0.0290	0.208	0.195	0.399	0.0030	0.208	0.187	0.580	0.0594	0.254	0.263	0.936
	0.2	-0.0791	0.206	0.214	0.321	-0.0454	0.188	0.172	0.523	0.0025	0.185	0.155	0.693	0.0991	0.244	0.266	0.968
	0.4	-0.1168	0.181	0.208	0.495	-0.0651	0.160	0.147	0.692	0.0019	0.156	0.118	0.832	0.1541	0.222	0.274	0.989
	0.6	-0.1429	0.161	0.206	0.646	-0.0781	0.140	0.132	0.810	0.0014	0.136	0.093	0.904	0.1966	0.200	0.282	0.997
	0.8	-0.1658	0.141	0.205	0.774	-0.0882	0.122	0.120	0.899	0.0013	0.119	0.071	0.957	0.2374	0.172	0.291	0.999
	1	-0.1980	0.103	0.203	0.947	-0.0994	0.100	0.108	0.986	0.0013	0.100	0.042	0.995	0.3024	0.102	0.305	1
0.3	0	0.0798	0.216	0.213	0.038	0.0755	0.217	0.213	0.115	0.0619	0.223	0.221	0.265	0.0813	0.243	0.239	0.841
	0.1	0.0184	0.211	0.195	0.104	0.0289	0.195	0.171	0.261	0.0598	0.188	0.163	0.470	0.1419	0.226	0.250	0.937
	0.2	-0.0180	0.202	0.189	0.181	0.0029	0.179	0.150	0.389	0.0485	0.167	0.135	0.608	0.1772	0.212	0.260	0.971
	0.4	-0.0650	0.185	0.183	0.328	-0.0280	0.156	0.126	0.581	0.0359	0.142	0.102	0.772	0.2214	0.187	0.276	0.992
	0.6	-0.0996	0.166	0.181	0.481	-0.0475	0.138	0.111	0.718	0.0286	0.125	0.080	0.865	0.2522	0.165	0.288	0.998
	0.8	-0.1306	0.145	0.180	0.640	-0.0629	0.121	0.100	0.833	0.0234	0.112	0.063	0.935	0.2781	0.142	0.299	1
	1	-0.1739	0.100	0.179	0.878	-0.0788	0.099	0.089	0.965	0.0189	0.099	0.045	0.999	0.3099	0.100	0.312	1
0.6	0	0.1150	0.205	0.216	0.020	0.1124	0.206	0.216	0.075	0.1169	0.212	0.224	0.206	0.1212	0.233	0.245	0.824
	0.1	0.0548	0.206	0.198	0.067	0.0598	0.191	0.176	0.193	0.0871	0.181	0.169	0.406	0.1781	0.212	0.258	0.931
	0.2	0.0178	0.201	0.189	0.126	0.0304	0.178	0.154	0.313	0.0710	0.163	0.142	0.548	0.2094	0.196	0.270	0.964
	0.4	-0.0327	0.188	0.180	0.251	-0.0051	0.157	0.128	0.496	0.0532	0.140	0.108	0.728	0.2471	0.171	0.285	0.990
	0.6	-0.0709	0.172	0.174	0.385	-0.0283	0.140	0.110	0.648	0.0426	0.124	0.087	0.833	0.2720	0.151	0.297	0.998
	0.8	-0.1067	0.151	0.171	0.547	-0.0472	0.123	0.095	0.775	0.0352	0.111	0.069	0.911	0.2921	0.131	0.306	0.999
	1	-0.1607	0.099	0.166	0.805	-0.0672	0.098	0.079	0.934	0.0287	0.098	0.050	0.984	0.3147	0.099	0.317	1
1	0	0.1407	0.196	0.224	0.014	0.1395	0.198	0.225	0.056	0.1448	0.204	0.233	0.168	0.1503	0.225	0.255	0.811
	0.1	0.0838	0.202	0.205	0.046	0.0848	0.188	0.186	0.145	0.1083	0.177	0.180	0.355	0.2036	0.202	0.269	0.922
	0.2	0.0474	0.200	0.196	0.093	0.0535	0.178	0.164	0.257	0.0888	0.161	0.153	0.499	0.2314	0.185	0.280	0.961
	0.4	-0.0041	0.190	0.183	0.193	0.0150	0.159	0.135	0.429	0.0676	0.139	0.119	0.677	0.2639	0.161	0.294	0.989
	0.6	-0.0448	0.176	0.175	0.311	-0.0113	0.143	0.115	0.582	0.0545	0.124	0.096	0.792	0.2846	0.142	0.304	0.997
	0.8	-0.0851	0.156	0.166	0.458	-0.0330	0.126	0.096	0.723	0.0450	0.112	0.077	0.883	0.3011	0.124	0.312	0.999
	1	-0.1501	0.098	0.156	0.728	-0.0577	0.097	0.071	0.900	0.0368	0.097	0.055	0.971	0.3188	0.098	0.321	1

Table A3. Impact of α_0 and ω on the operating characteristics for scenarios S9 to S12 (Weibull distribution for historical and new data, positive prior-data conflict), with σ_H^2 equivalent to 329 events.

		**Control arm: positive prior-data conflict**															
**Parameters**	**Treatment effect**	**S9: Null scenario HR = 1**				**S10: Disappointing effect scenario HR = 0.886**				**S11: Historical effect scenario HR = 0.786**				**S12: Anticipated effect scenario HR = 0.55**			
α_0	ω	Bias	SD	RMSE	Alpha	Bias	SD	RMSE	Power	Bias	SD	RMSE	Power	Bias	SD	RMSE	Power
0	0	0.0009	0.281	0.280	0.102	-0.0003	0.280	0.284	0.201	0.0008	0.286	0.291	0.354	-0.0089	0.315	0.315	0.764
	0.1	-0.0498	0.256	0.248	0.213	-0.0301	0.244	0.227	0.354	0.0007	0.246	0.223	0.538	0.0447	0.300	0.300	0.882
	0.2	-0.0801	0.236	0.233	0.326	-0.0463	0.219	0.198	0.486	0.0005	0.219	0.185	0.662	0.0872	0.285	0.295	0.931
	0.4	-0.1205	0.205	0.221	0.494	-0.0669	0.183	0.164	0.684	0.0004	0.181	0.139	0.816	0.1475	0.256	0.294	0.976
	0.6	-0.1493	0.179	0.215	0.648	-0.0808	0.157	0.143	0.801	0.0003	0.153	0.106	0.902	0.1947	0.227	0.297	0.994
	0.8	-0.1744	0.153	0.212	0.791	-0.0923	0.133	0.127	0.897	0	0.130	0.076	0.959	0.2402	0.192	0.301	0.999
	1	-0.2075	0.107	0.211	0.975	-0.1048	0.102	0.112	0.992	0	0.102	0.038	0.998	0.3125	0.104	0.315	1
0.3	0	-0.0605	0.246	0.237	0.132	-0.0640	0.248	0.244	0.274	-0.0616	0.256	0.250	0.481	-0.0817	0.287	0.286	0.905
	0.1	-0.1043	0.218	0.214	0.278	-0.0799	0.210	0.192	0.472	-0.0557	0.217	0.191	0.686	-0.0180	0.285	0.279	0.969
	0.2	-0.1285	0.198	0.207	0.402	-0.0877	0.186	0.168	0.620	-0.0447	0.192	0.157	0.801	0.0287	0.277	0.277	0.987
	0.4	-0.1577	0.171	0.205	0.591	-0.0968	0.155	0.144	0.783	-0.0315	0.161	0.117	0.910	0.0957	0.258	0.277	0.999
	0.6	-0.1770	0.149	0.205	0.734	-0.1021	0.135	0.130	0.880	-0.0232	0.138	0.089	0.959	0.1496	0.234	0.280	1
	0.8	-0.1921	0.130	0.207	0.851	-0.1061	0.118	0.122	0.949	-0.0170	0.120	0.065	0.985	0.2033	0.202	0.284	1
	1	-0.2083	0.103	0.212	0.975	-0.1094	0.101	0.116	0.995	-0.0110	0.101	0.039	0.999	0.2954	0.102	0.297	1
0.6	0	-0.0892	0.231	0.234	0.163	-0.0938	0.235	0.242	0.336	-0.0926	0.243	0.248	0.562	-0.1145	0.275	0.287	0.950
	0.1	-0.1272	0.202	0.214	0.331	-0.1012	0.197	0.192	0.550	-0.0769	0.208	0.193	0.763	-0.0529	0.279	0.283	0.986
	0.2	-0.1474	0.183	0.208	0.462	-0.1046	0.175	0.170	0.685	-0.0614	0.185	0.160	0.853	-0.0068	0.275	0.280	0.996
	0.4	-0.1710	0.158	0.207	0.641	-0.1082	0.147	0.146	0.825	-0.0437	0.156	0.121	0.942	0.0606	0.260	0.278	1
	0.6	-0.1862	0.139	0.208	0.772	-0.1104	0.129	0.134	0.909	-0.0326	0.135	0.093	0.973	0.1169	0.240	0.278	1
	0.8	-0.1980	0.122	0.210	0.879	-0.1120	0.114	0.126	0.961	-0.0241	0.119	0.069	0.991	0.1753	0.209	0.279	1
	1	-0.2098	0.102	0.214	0.976	-0.1131	0.100	0.120	0.995	-0.0163	0.100	0.042	1	0.2850	0.101	0.287	1
1	0	-0.1108	0.221	0.238	0.194	-0.1162	0.226	0.246	0.400	-0.1158	0.235	0.253	0.628	-0.1391	0.268	0.293	0.971
	0.1	-0.1433	0.192	0.218	0.378	-0.1171	0.190	0.197	0.613	-0.0933	0.202	0.199	0.810	-0.0809	0.274	0.289	0.993
	0.2	-0.1603	0.174	0.213	0.503	-0.1168	0.168	0.175	0.730	-0.0749	0.181	0.167	0.889	-0.0363	0.272	0.286	0.998
	0.4	-0.1804	0.150	0.211	0.677	-0.1168	0.142	0.152	0.860	-0.0540	0.154	0.128	0.955	0.0302	0.262	0.282	1
	0.6	-0.1926	0.132	0.212	0.800	-0.1168	0.125	0.139	0.927	-0.0409	0.135	0.100	0.980	0.0872	0.245	0.280	1
	0.8	-0.2022	0.118	0.213	0.893	-0.1168	0.112	0.130	0.966	-0.0307	0.118	0.075	0.993	0.1487	0.217	0.277	1
	1	-0.2116	0.100	0.216	0.973	-0.1165	0.099	0.124	0.996	-0.0211	0.099	0.046	1	0.2761	0.100	0.279	1
