# Supplementary material for: Incorporating individual historical controls and aggregate treatment effect estimates into a Bayesian survival trial: a simulation study
Source: BMC Med Res Methodol. 2019 Apr 24;19:85. doi: 10.1186/s12874-019-0714-z (PMC6480797; doi:10.1186/s12874-019-0714-z)
Supplement: Supplementary file 9 — Tables A4-A6. Impact of α0 and ω on the operating characteristics for scenarios S13 to S24. These tables describe the impact of α0 and ω on the operating characteristics for scenarios S13 to S16 (Table A4), when historical and new control arm are commensurate, for scenarios S17 to S20 (Table A5), when there is a negative prior-data conflict, and for scenarios S21 to S24 (Table A6), when there is a positive prior-data conflict. A piecewise exponential distribution with 3 pieces is used to generate and analyse historical and new data, and ${\upsigma}_{\mathrm{H}}^2$σH2 is equivalent to 329 events. (PDF 483 kb) [file 12874_2019_714_MOESM9_ESM.pdf]

Table A4. Impact of α_0 and ω on the operating characteristics for scenarios S13 to S16 (Piecewise distribution for historical and new data, commensurate control arm), with σ_H^2 equivalent to 329 events.

Commensurate control arm																	
Parameters \ Treatment effect		S13: Null scenario HR = 1				S14: Disappointing effect scenario HR = 0.886				S15: Historical effect scenario HR = 0.786				S16: Anticipated effect scenario HR = 0.55			
α_0	ω	Bias	SD	RMSE	Alpha	Bias	SD	RMSE	Power	Bias	SD	RMSE	Power	Bias	SD	RMSE	Power
0	0	-0.0018	0.274	0.280	0.104	-0.0050	0.277	0.283	0.211	-0.0029	0.287	0.296	0.333	-0.0139	0.311	0.320	0.770
	0.1	-0.0514	0.249	0.248	0.217	-0.0333	0.242	0.225	0.364	-0.0019	0.248	0.229	0.500	0.0419	0.296	0.304	0.880
	0.2	-0.0813	0.230	0.235	0.328	-0.0491	0.217	0.195	0.489	-0.0018	0.221	0.192	0.639	0.0843	0.281	0.300	0.935
	0.4	-0.1205	0.200	0.222	0.508	-0.0685	0.182	0.162	0.680	-0.0010	0.184	0.146	0.799	0.1441	0.254	0.300	0.979
	0.6	-0.1483	0.175	0.216	0.654	-0.0813	0.156	0.142	0.808	-0.0009	0.157	0.112	0.883	0.1910	0.226	0.303	0.993
	0.8	-0.1733	0.148	0.213	0.794	-0.0919	0.133	0.126	0.902	-0.0007	0.132	0.080	0.953	0.2367	0.192	0.306	0.998
	1	-0.2070	0.102	0.211	0.972	-0.1039	0.102	0.111	0.992	-0.0001	0.103	0.038	0.998	0.3132	0.104	0.315	1
0.3	0	-0.0227	0.241	0.227	0.095	-0.0251	0.246	0.233	0.225	-0.0251	0.256	0.246	0.385	-0.0439	0.283	0.276	0.874
	0.1	-0.0732	0.218	0.206	0.226	-0.0509	0.210	0.181	0.408	-0.0177	0.216	0.183	0.598	0.0280	0.276	0.271	0.951
	0.2	-0.1016	0.200	0.200	0.341	-0.0641	0.187	0.158	0.552	-0.0137	0.191	0.151	0.731	0.0732	0.265	0.273	0.981
	0.4	-0.1368	0.174	0.197	0.527	-0.0791	0.157	0.135	0.734	-0.0089	0.160	0.112	0.862	0.1353	0.242	0.278	0.995
	0.6	-0.1598	0.152	0.198	0.673	-0.0882	0.136	0.122	0.851	-0.0060	0.138	0.085	0.932	0.1833	0.217	0.284	0.998
	0.8	-0.1796	0.132	0.201	0.812	-0.0951	0.119	0.114	0.929	-0.0036	0.120	0.062	0.971	0.2295	0.186	0.291	0.999
	1	-0.2013	0.101	0.205	0.966	-0.1017	0.101	0.109	0.991	-0.0011	0.101	0.038	0.999	0.3027	0.102	0.305	1
0.6	0	-0.0299	0.227	0.214	0.096	-0.0325	0.233	0.220	0.239	-0.0330	0.243	0.234	0.421	-0.0510	0.272	0.266	0.906
	0.1	-0.0791	0.206	0.196	0.234	-0.0563	0.198	0.172	0.438	-0.0228	0.204	0.173	0.642	0.0209	0.267	0.264	0.970
	0.2	-0.1065	0.189	0.193	0.352	-0.0683	0.176	0.151	0.579	-0.0175	0.181	0.142	0.763	0.0656	0.258	0.267	0.990
	0.4	-0.1394	0.165	0.192	0.534	-0.0815	0.149	0.130	0.752	-0.0116	0.152	0.106	0.881	0.1276	0.237	0.273	0.997
	0.6	-0.1614	0.145	0.195	0.680	-0.0894	0.130	0.120	0.863	-0.0077	0.132	0.081	0.943	0.1757	0.214	0.279	0.999
	0.8	-0.1788	0.127	0.198	0.807	-0.0952	0.116	0.113	0.933	-0.0048	0.117	0.060	0.976	0.2221	0.184	0.286	0.999
	1	-0.1984	0.100	0.203	0.958	-0.1007	0.100	0.108	0.989	-0.0020	0.100	0.039	0.998	0.2973	0.101	0.300	1
1	0	-0.0345	0.218	0.208	0.104	-0.0376	0.224	0.215	0.255	-0.0381	0.234	0.228	0.454	-0.0555	0.264	0.261	0.925
	0.1	-0.0825	0.197	0.193	0.244	-0.0596	0.190	0.168	0.459	-0.0262	0.197	0.170	0.669	0.0153	0.261	0.261	0.981
	0.2	-0.1088	0.182	0.190	0.357	-0.0706	0.170	0.148	0.597	-0.0202	0.175	0.140	0.783	0.0595	0.253	0.264	0.992
	0.4	-0.1403	0.159	0.191	0.541	-0.0827	0.144	0.129	0.763	-0.0133	0.147	0.105	0.893	0.1207	0.234	0.271	0.997
	0.6	-0.1610	0.141	0.193	0.686	-0.0898	0.127	0.119	0.867	-0.0090	0.129	0.081	0.945	0.1685	0.212	0.277	0.999
	0.8	-0.1779	0.124	0.196	0.807	-0.0951	0.113	0.113	0.934	-0.0056	0.115	0.060	0.977	0.2154	0.183	0.283	0.999
	1	-0.1963	0.099	0.201	0.949	-0.1000	0.099	0.108	0.986	-0.0026	0.099	0.041	0.998	0.2930	0.100	0.295	1

Table A5. Impact of α_0 and ω on the operating characteristics for scenarios S17 to S20 (Piecewise distribution for historical and new data, negative prior-data conflict), with σ^2_H equivalent to 329 events.

Control arm: negative prior-data conflict																	
Treatment effect / Parameters		S17: Null scenario HR = 1				S18: Disappointing effect scenario HR = 0.886				S19: Historical effect scenario HR = 0.786				S20: Anticipated effect scenario HR = 0.55			
α_0	ω	Bias	SD	RMSE	Alpha	Bias	SD	RMSE	Power	Bias	SD	RMSE	Power	Bias	SD	RMSE	Power
0	0	-0.0068	0.251	0.254	0.109	-0.0046	0.256	0.261	0.219	-0.0111	0.262	0.265	0.368	-0.0079	0.283	0.286	0.819
	0.1	-0.0581	0.230	0.228	0.221	-0.0318	0.223	0.209	0.387	-0.0086	0.224	0.202	0.559	0.0582	0.271	0.280	0.913
	0.2	-0.0878	0.212	0.219	0.336	-0.0470	0.200	0.183	0.515	-0.0074	0.199	0.168	0.688	0.0997	0.257	0.281	0.954
	0.4	-0.1255	0.185	0.211	0.511	-0.0662	0.170	0.154	0.687	-0.0054	0.166	0.127	0.836	0.1558	0.232	0.286	0.984
	0.6	-0.1516	0.162	0.209	0.650	-0.0786	0.148	0.136	0.808	-0.0038	0.144	0.100	0.916	0.1994	0.207	0.292	0.994
	0.8	-0.1740	0.139	0.209	0.793	-0.0894	0.127	0.122	0.896	-0.0025	0.124	0.074	0.963	0.2408	0.177	0.299	0.998
	1	-0.2030	0.101	0.207	0.966	-0.1015	0.101	0.109	0.989	-0.0012	0.101	0.040	0.997	0.3080	0.102	0.310	1
0.3	0	0.1096	0.224	0.235	0.028	0.1107	0.229	0.242	0.088	0.1041	0.236	0.245	0.216	0.1019	0.258	0.268	0.764
	0.1	0.0475	0.221	0.213	0.083	0.0581	0.209	0.196	0.211	0.0760	0.202	0.185	0.397	0.1616	0.236	0.273	0.899
	0.2	0.0092	0.214	0.203	0.148	0.0286	0.194	0.172	0.327	0.0619	0.180	0.154	0.545	0.1949	0.218	0.280	0.947
	0.4	-0.0424	0.197	0.192	0.286	-0.0072	0.170	0.144	0.506	0.0456	0.153	0.118	0.724	0.2364	0.190	0.292	0.980
	0.6	-0.0818	0.179	0.186	0.429	-0.0312	0.150	0.124	0.655	0.0357	0.134	0.093	0.833	0.2651	0.166	0.301	0.992
	0.8	-0.1188	0.155	0.182	0.588	-0.0518	0.131	0.107	0.790	0.0284	0.118	0.072	0.921	0.2895	0.142	0.310	0.998
	1	-0.1724	0.100	0.177	0.865	-0.0751	0.100	0.085	0.954	0.0212	0.100	0.045	0.989	0.3188	0.101	0.321	1
0.6	0	0.1542	0.216	0.249	0.014	0.1543	0.217	0.255	0.055	0.1473	0.224	0.257	0.164	0.1430	0.248	0.278	0.735
	0.1	0.0954	0.216	0.228	0.051	0.0974	0.206	0.212	0.150	0.1095	0.196	0.200	0.332	0.1962	0.222	0.283	0.884
	0.2	0.0570	0.213	0.216	0.097	0.0644	0.194	0.188	0.251	0.0898	0.177	0.169	0.467	0.2252	0.203	0.290	0.939
	0.4	0.0029	0.202	0.201	0.204	0.0237	0.174	0.157	0.411	0.0671	0.152	0.132	0.653	0.2600	0.175	0.301	0.975
	0.6	-0.0407	0.187	0.189	0.321	-0.0056	0.155	0.133	0.570	0.0532	0.134	0.106	0.782	0.2831	0.154	0.310	0.990
	0.8	-0.0841	0.165	0.179	0.480	-0.0307	0.135	0.110	0.714	0.0425	0.118	0.083	0.884	0.3021	0.132	0.317	0.997
	1	-0.1578	0.099	0.163	0.764	-0.0625	0.099	0.075	0.908	0.0318	0.099	0.052	0.977	0.3237	0.100	0.326	0.999
1	0	0.1834	0.211	0.264	0.011	0.1828	0.209	0.269	0.041	0.1754	0.216	0.271	0.138	0.1695	0.240	0.289	0.719
	0.1	0.1287	0.211	0.244	0.035	0.1252	0.202	0.228	0.121	0.1328	0.192	0.215	0.291	0.2177	0.212	0.293	0.867
	0.2	0.0921	0.211	0.231	0.072	0.0914	0.193	0.204	0.202	0.1101	0.176	0.185	0.408	0.2435	0.193	0.299	0.928
	0.4	0.0382	0.204	0.214	0.156	0.0475	0.176	0.172	0.356	0.0838	0.153	0.147	0.601	0.2736	0.167	0.309	0.971
	0.6	-0.0072	0.192	0.199	0.256	0.0157	0.160	0.146	0.497	0.0669	0.135	0.120	0.735	0.2934	0.146	0.316	0.987
	0.8	-0.0552	0.172	0.184	0.398	-0.0134	0.140	0.119	0.647	0.0535	0.120	0.094	0.846	0.3093	0.127	0.322	0.996
	1	-0.1470	0.099	0.153	0.683	-0.0533	0.099	0.069	0.865	0.0395	0.099	0.058	0.959	0.3272	0.099	0.330	0.999

Table A6. Impact of α_0 and ω on the operating characteristics for scenarios S21 to S24 (Piecewise distribution for historical and new data, positive prior-data conflict), with σ_H^2 equivalent to 329 events.

		Control arm: positive prior-data conflict															
Parameters \ Treatment effect		S21: Null scenario HR = 1				S22: Disappointing effect scenario HR = 0.886				S23: Historical effect scenario HR = 0.786				S24: Anticipated effect scenario HR = 0.55			
α_0	ω	Bias	SD	RMSE	Alpha	Bias	SD	RMSE	Power	Bias	SD	RMSE	Power	Bias	SD	RMSE	Power
0	0	0.0015	0.299	0.307	0.108	-0.0051	0.307	0.316	0.200	-0.0106	0.315	0.323	0.323	-0.0186	0.345	0.349	0.707
	0.1	-0.0481	0.272	0.270	0.203	-0.0303	0.269	0.255	0.308	-0.0082	0.274	0.251	0.478	0.0491	0.324	0.328	0.833
	0.2	-0.0782	0.250	0.253	0.310	-0.0453	0.242	0.222	0.469	-0.0067	0.244	0.210	0.607	0.0936	0.305	0.320	0.900
	0.4	-0.1188	0.216	0.236	0.502	-0.0655	0.202	0.182	0.654	-0.0050	0.202	0.157	0.775	0.1555	0.271	0.314	0.962
	0.6	-0.1487	0.186	0.227	0.659	-0.0801	0.171	0.156	0.788	-0.0036	0.169	0.120	0.881	0.2037	0.237	0.314	0.985
	0.8	-0.1748	0.156	0.220	0.799	-0.0919	0.143	0.135	0.892	-0.0021	0.140	0.087	0.953	0.2499	0.197	0.316	0.996
	1	-0.2114	0.103	0.215	0.983	-0.1063	0.104	0.112	0.995	0	0.104	0.035	0.999	0.3212	0.105	0.323	1
0.3	0	-0.1517	0.259	0.287	0.214	-0.1585	0.268	0.299	0.392	-0.1618	0.277	0.309	0.574	-0.1670	0.311	0.343	0.926
	0.1	-0.1749	0.222	0.255	0.392	-0.1476	0.227	0.240	0.591	-0.1207	0.242	0.244	0.756	-0.0919	0.313	0.327	0.971
	0.2	-0.1871	0.197	0.243	0.537	-0.1413	0.200	0.211	0.722	-0.0973	0.217	0.206	0.853	-0.0408	0.308	0.319	0.989
	0.4	-0.2017	0.165	0.233	0.728	-0.1343	0.166	0.178	0.867	-0.0694	0.183	0.159	0.945	0.0363	0.291	0.310	0.997
	0.6	-0.2107	0.142	0.229	0.848	-0.1299	0.142	0.157	0.936	-0.0503	0.157	0.123	0.978	0.1006	0.266	0.304	0.999
	0.8	-0.2179	0.123	0.228	0.928	-0.1266	0.123	0.143	0.973	-0.0355	0.133	0.090	0.993	0.1683	0.230	0.301	1
	1	-0.2244	0.101	0.228	0.993	-0.1227	0.102	0.128	0.999	-0.0198	0.102	0.042	1	0.2948	0.102	0.297	1
0.6	0	-0.2058	0.245	0.308	0.301	-0.2122	0.254	0.320	0.506	-0.2147	0.264	0.329	0.700	-0.2190	0.298	0.362	0.965
	0.1	-0.2145	0.205	0.273	0.510	-0.1860	0.215	0.261	0.713	-0.1611	0.235	0.265	0.854	-0.1505	0.308	0.347	0.990
	0.2	-0.2191	0.182	0.260	0.648	-0.1724	0.191	0.231	0.822	-0.1312	0.214	0.230	0.922	-0.1012	0.307	0.337	0.997
	0.4	-0.2241	0.152	0.247	0.810	-0.1560	0.160	0.195	0.926	-0.0951	0.183	0.181	0.974	-0.0249	0.298	0.324	0.999
	0.6	-0.2274	0.133	0.241	0.901	-0.1464	0.139	0.173	0.965	-0.0714	0.160	0.145	0.990	0.0418	0.280	0.314	1
	0.8	-0.2295	0.117	0.238	0.953	-0.1388	0.121	0.155	0.987	-0.0512	0.136	0.106	0.998	0.1175	0.247	0.304	1
	1	-0.2312	0.100	0.234	0.995	-0.1311	0.101	0.136	0.999	-0.0297	0.101	0.047	1	0.2812	0.101	0.283	1
1	0	-0.2394	0.235	0.327	0.373	-0.2460	0.245	0.338	0.597	-0.2479	0.255	0.347	0.777	-0.2513	0.290	0.378	0.981
	0.1	-0.2386	0.196	0.289	0.593	-0.2107	0.210	0.279	0.784	-0.1882	0.231	0.285	0.904	-0.1892	0.302	0.364	0.997
	0.2	-0.2381	0.174	0.273	0.722	-0.1921	0.188	0.247	0.876	-0.1551	0.213	0.249	0.951	-0.1439	0.305	0.355	0.998
	0.4	-0.2377	0.146	0.258	0.857	-0.1713	0.159	0.211	0.949	-0.1149	0.186	0.200	0.985	-0.0706	0.300	0.340	1
	0.6	-0.2374	0.128	0.250	0.925	-0.1585	0.139	0.187	0.976	-0.0872	0.162	0.163	0.995	-0.0023	0.286	0.327	1
	0.8	-0.2370	0.114	0.245	0.966	-0.1480	0.121	0.166	0.992	-0.0641	0.139	0.124	1	0.0761	0.259	0.312	1
	1	-0.2363	0.100	0.240	0.996	-0.1375	0.100	0.143	0.999	-0.0372	0.100	0.053	1	0.2706	0.100	0.273	1
