# Supplementary material for: Incorporating individual historical controls and aggregate treatment effect estimates into a Bayesian survival trial: a simulation study
Source: BMC Med Res Methodol. 2019 Apr 24;19:85. doi: 10.1186/s12874-019-0714-z (PMC6480797; doi:10.1186/s12874-019-0714-z)
Supplement: Supplementary file 10 — Tables A7-A12. Impact of α0 and ω on the operating characteristics for scenarios S1 to S12 when ${\upsigma}_{\mathrm{H}}^2$σH2 is equivalent to 66 or 22 events. These tables describe the impact of α0 and ω on the operating characteristics for scenarios S1 to S4 (Tables A7 and A10), when historical and new control arm are commensurate, for scenarios S5 to S8 (Tables A8 and A11), when there is a negative prior-data conflict, and for scenarios S9 to S12 (Tables A9 and A12), when there is a positive prior-data conflict. A Weibull distribution is used to generate and analyse historical and new data, and ${\upsigma}_{\mathrm{H}}^2$σH2 is equivalent to 66 (Tables A7-A9) or 22 (Tables A10-A12) events. (PDF 689 kb) [file 12874_2019_714_MOESM10_ESM.pdf]

Table A7. Impact of α_0 and ω on the operating characteristics for scenarios S1 to S4 (Weibull distribution for historical and new data, commensurate control arm), with σ_H^2 equivalent to 66 events.

Commensurate control arm																	
Parameters \ Treatment effect		S1: Null scenario HR = 1				S2: Disappointing effect scenario HR = 0.886				S3: Historical effect scenario HR = 0.786				S4: Anticipated effect scenario HR = 0.55			
α_0	ω	Bias	SD	RMSE	Alpha	Bias	SD	RMSE	Power	Bias	SD	RMSE	Power	Bias	SD	RMSE	Power
0	0	0.0024	0.263	0.262	0.100	0.0015	0.268	0.267	0.208	0.0022	0.275	0.275	0.359	-0.0075	0.304	0.298	0.794
	0.1	-0.0338	0.246	0.235	0.127	-0.0182	0.239	0.226	0.259	0.0020	0.243	0.225	0.422	0.0381	0.279	0.277	0.835
	0.2	-0.0556	0.234	0.221	0.147	-0.0297	0.225	0.203	0.289	0.0015	0.228	0.199	0.460	0.0706	0.269	0.268	0.854
	0.4	-0.0825	0.218	0.203	0.174	-0.0434	0.207	0.177	0.324	0.0011	0.209	0.168	0.502	0.1142	0.250	0.257	0.879
	0.6	-0.0998	0.205	0.193	0.189	-0.0518	0.195	0.161	0.344	0.0010	0.197	0.149	0.518	0.1455	0.233	0.250	0.889
	0.8	-0.1130	0.193	0.184	0.199	-0.0578	0.186	0.148	0.354	0.0009	0.188	0.135	0.532	0.1719	0.215	0.243	0.895
	1	-0.1250	0.181	0.176	0.204	-0.0627	0.178	0.139	0.365	0.0008	0.181	0.124	0.542	0.2014	0.186	0.235	0.900
0.3	0	-0.0058	0.232	0.214	0.084	-0.0071	0.238	0.220	0.211	-0.0070	0.245	0.231	0.396	-0.0158	0.274	0.259	0.883
	0.1	-0.0405	0.218	0.194	0.114	-0.0255	0.214	0.186	0.265	-0.0068	0.218	0.188	0.469	0.0302	0.258	0.243	0.912
	0.2	-0.0599	0.209	0.184	0.135	-0.0349	0.202	0.169	0.296	-0.0063	0.205	0.167	0.504	0.0625	0.249	0.237	0.930
	0.4	-0.0821	0.195	0.174	0.156	-0.0451	0.188	0.151	0.332	-0.0042	0.191	0.144	0.545	0.1047	0.233	0.229	0.944
	0.6	-0.0954	0.186	0.168	0.169	-0.0509	0.180	0.140	0.353	-0.0030	0.182	0.131	0.565	0.1338	0.217	0.224	0.951
	0.8	-0.1049	0.178	0.164	0.181	-0.0546	0.174	0.134	0.366	-0.0021	0.176	0.122	0.576	0.1580	0.201	0.219	0.954
	1	-0.1124	0.171	0.161	0.187	-0.0573	0.169	0.129	0.376	-0.0014	0.172	0.115	0.588	0.1829	0.177	0.215	0.955
0.6	0	-0.0095	0.219	0.200	0.082	-0.0108	0.225	0.206	0.222	-0.0111	0.233	0.218	0.418	-0.0199	0.261	0.248	0.911
	0.1	-0.0424	0.207	0.183	0.114	-0.0277	0.203	0.176	0.278	-0.0107	0.208	0.178	0.499	0.0255	0.248	0.233	0.943
	0.2	-0.0602	0.198	0.175	0.135	-0.0360	0.192	0.161	0.309	-0.0082	0.196	0.159	0.537	0.0574	0.240	0.228	0.953
	0.4	-0.0805	0.186	0.166	0.154	-0.0449	0.180	0.144	0.344	-0.0057	0.184	0.138	0.571	0.0982	0.225	0.221	0.963
	0.6	-0.0923	0.178	0.161	0.169	-0.0499	0.173	0.136	0.363	-0.0042	0.176	0.127	0.588	0.1265	0.211	0.215	0.967
	0.8	-0.1004	0.172	0.158	0.174	-0.0529	0.168	0.130	0.375	-0.0031	0.171	0.119	0.604	0.1499	0.195	0.211	0.969
	1	-0.1068	0.166	0.156	0.183	-0.0551	0.165	0.127	0.386	-0.0023	0.167	0.114	0.612	0.1735	0.172	0.206	0.971
1	0	-0.0121	0.210	0.193	0.087	-0.0137	0.216	0.200	0.233	-0.0141	0.223	0.212	0.448	-0.0230	0.252	0.242	0.929
	0.1	-0.0433	0.198	0.178	0.119	-0.0290	0.195	0.171	0.288	-0.0124	0.201	0.173	0.522	0.0217	0.241	0.228	0.957
	0.2	-0.0600	0.190	0.170	0.137	-0.0366	0.186	0.157	0.323	-0.0100	0.190	0.155	0.555	0.0527	0.234	0.223	0.965
	0.4	-0.0791	0.180	0.163	0.158	-0.0445	0.175	0.142	0.356	-0.0068	0.178	0.136	0.594	0.0927	0.219	0.216	0.971
	0.6	-0.0899	0.172	0.159	0.171	-0.0490	0.169	0.134	0.376	-0.0050	0.172	0.126	0.612	0.1203	0.206	0.210	0.972
	0.8	-0.0972	0.167	0.156	0.177	-0.0517	0.164	0.129	0.389	-0.0039	0.167	0.119	0.621	0.1431	0.191	0.205	0.975
	1	-0.1029	0.162	0.154	0.180	-0.0536	0.161	0.126	0.395	-0.0031	0.164	0.114	0.627	0.1662	0.169	0.200	0.976

Table A8. Impact of α_0 and ω on the operating characteristics for scenarios S5 to S8 (Weibull distribution for historical and new data, negative prior-data conflict), with σ_H^2 equivalent to 66 events.

Control arm: negative prior-data conflict																	
Parameters \ Treatment effect		S5: Null scenario HR = 1				S6: Disappointing effect scenario HR = 0.886				S7: Historical effect scenario HR = 0.786				S8: Anticipated effect scenario HR = 0.55			
α_0	ω	Bias	SD	RMSE	Alpha	Bias	SD	RMSE	Power	Bias	SD	RMSE	Power	Bias	SD	RMSE	Power
0	0	0.0003	0.241	0.243	0.104	0.0008	0.240	0.243	0.224	0.0036	0.245	0.247	0.382	-0.0024	0.263	0.264	0.849
	0.1	-0.0325	0.227	0.221	0.134	-0.0217	0.221	0.209	0.277	0.0035	0.223	0.207	0.448	0.0456	0.251	0.253	0.882
	0.2	-0.0528	0.217	0.210	0.151	-0.0323	0.209	0.190	0.308	0.0035	0.211	0.185	0.478	0.0758	0.242	0.247	0.902
	0.4	-0.0773	0.202	0.195	0.171	-0.0444	0.194	0.169	0.345	0.0034	0.195	0.160	0.513	0.1150	0.227	0.240	0.919
	0.6	-0.0926	0.192	0.186	0.182	-0.0515	0.185	0.156	0.362	0.0034	0.186	0.144	0.534	0.1425	0.213	0.235	0.927
	0.8	-0.1042	0.183	0.178	0.192	-0.0564	0.178	0.146	0.375	0.0032	0.179	0.134	0.550	0.1648	0.198	0.231	0.930
	1	-0.1151	0.173	0.171	0.196	-0.0603	0.172	0.139	0.386	0.0028	0.174	0.124	0.552	0.1884	0.177	0.224	0.932
0.3	0	0.0798	0.216	0.213	0.038	0.0755	0.217	0.213	0.115	0.0619	0.223	0.221	0.265	0.0813	0.243	0.239	0.841
	0.1	0.0388	0.209	0.190	0.052	0.0460	0.203	0.181	0.154	0.0602	0.204	0.189	0.327	0.1239	0.227	0.239	0.880
	0.2	0.0151	0.202	0.177	0.062	0.0302	0.194	0.163	0.180	0.0599	0.193	0.170	0.362	0.1482	0.217	0.240	0.897
	0.4	-0.0134	0.191	0.160	0.074	0.0130	0.182	0.142	0.205	0.0538	0.181	0.150	0.398	0.1769	0.202	0.242	0.914
	0.6	-0.0316	0.182	0.147	0.080	0.0033	0.174	0.129	0.221	0.0503	0.174	0.139	0.418	0.1947	0.190	0.244	0.920
	0.8	-0.0452	0.174	0.137	0.086	-0.0033	0.169	0.120	0.228	0.0481	0.169	0.131	0.431	0.2078	0.180	0.245	0.928
	1	-0.0567	0.164	0.127	0.087	-0.0082	0.164	0.113	0.236	0.0465	0.166	0.125	0.441	0.2192	0.169	0.246	0.928
0.6	0	0.1150	0.205	0.216	0.020	0.1124	0.206	0.216	0.075	0.1169	0.212	0.224	0.206	0.1212	0.233	0.245	0.824
	0.1	0.0739	0.201	0.192	0.030	0.0796	0.195	0.184	0.107	0.0991	0.195	0.194	0.264	0.1593	0.215	0.248	0.867
	0.2	0.0497	0.196	0.176	0.038	0.0622	0.187	0.166	0.124	0.0904	0.186	0.177	0.296	0.1802	0.205	0.250	0.889
	0.4	0.0194	0.186	0.156	0.045	0.0430	0.177	0.144	0.144	0.0817	0.175	0.158	0.329	0.2038	0.191	0.254	0.904
	0.6	0.0001	0.178	0.140	0.050	0.0320	0.170	0.130	0.155	0.0768	0.169	0.147	0.348	0.2178	0.181	0.257	0.911
	0.8	-0.0143	0.169	0.127	0.050	0.0247	0.164	0.120	0.162	0.0736	0.165	0.140	0.359	0.2277	0.173	0.258	0.917
	1	-0.0271	0.160	0.114	0.054	0.0191	0.160	0.112	0.168	0.0713	0.161	0.134	0.368	0.2359	0.165	0.260	0.918
1	0	0.1407	0.196	0.224	0.014	0.1395	0.198	0.225	0.056	0.1448	0.204	0.233	0.168	0.1503	0.225	0.255	0.811
	0.1	0.1006	0.195	0.199	0.019	0.1054	0.189	0.193	0.080	0.1243	0.189	0.205	0.218	0.1849	0.207	0.259	0.853
	0.2	0.0763	0.191	0.183	0.025	0.0871	0.183	0.175	0.095	0.1141	0.181	0.188	0.250	0.2032	0.197	0.262	0.872
	0.4	0.0454	0.183	0.160	0.029	0.0666	0.173	0.152	0.111	0.1034	0.171	0.169	0.283	0.2232	0.184	0.266	0.891
	0.6	0.0251	0.175	0.142	0.032	0.0546	0.167	0.137	0.121	0.0975	0.165	0.158	0.296	0.2347	0.175	0.269	0.900
	0.8	0.0096	0.166	0.127	0.036	0.0465	0.161	0.126	0.123	0.0938	0.161	0.151	0.306	0.2428	0.168	0.270	0.904
	1	-0.0042	0.157	0.110	0.035	0.0404	0.157	0.116	0.129	0.0913	0.158	0.145	0.314	0.2489	0.162	0.272	0.908

Table A9. Impact of α_0 and ω on the operating characteristics for scenarios S9 to S12 (Weibull distribution for historical and new data, positive prior-data conflict), with σ_H^2 equivalent to 66 events.

Control arm: positive prior-data conflict																	
Treatment effect / Parameters		S9: Null scenario HR = 1				S10: Disappointing effect scenario HR = 0.886				S11: Historical effect scenario HR = 0.786				S12: Anticipated effect scenario, HR = 0.55			
α_0	ω	Bias	SD	RMSE	Alpha	Bias	SD	RMSE	Power	Bias	SD	RMSE	Power	Bias	SD	RMSE	Power
0	0	0.0009	0.281	0.280	0.102	-0.0003	0.280	0.284	0.201	0.0008	0.286	0.291	0.354	-0.0089	0.315	0.315	0.764
	0.1	-0.0360	0.263	0.250	0.130	-0.0221	0.256	0.240	0.252	0.0008	0.262	0.241	0.412	0.0430	0.300	0.294	0.796
	0.2	-0.0584	0.249	0.233	0.152	-0.0338	0.240	0.216	0.276	0.0007	0.245	0.212	0.450	0.0767	0.288	0.283	0.819
	0.4	-0.0867	0.230	0.214	0.177	-0.0477	0.219	0.186	0.309	0.0005	0.223	0.177	0.487	0.1224	0.266	0.270	0.844
	0.6	-0.1053	0.215	0.201	0.192	-0.0567	0.205	0.167	0.332	0.0004	0.209	0.154	0.510	0.1557	0.246	0.261	0.861
	0.8	-0.1198	0.202	0.191	0.201	-0.0632	0.195	0.153	0.341	0.0002	0.198	0.137	0.523	0.1845	0.226	0.254	0.867
	1	-0.1333	0.187	0.182	0.210	-0.0688	0.184	0.141	0.353	0.0001	0.187	0.123	0.535	0.2167	0.192	0.247	0.872
0.3	0	-0.0605	0.246	0.237	0.132	-0.0640	0.248	0.244	0.274	-0.0616	0.256	0.250	0.481	-0.0817	0.287	0.286	0.905
	0.1	-0.0885	0.227	0.216	0.169	-0.0741	0.225	0.208	0.339	-0.0489	0.234	0.206	0.524	-0.0249	0.279	0.268	0.924
	0.2	-0.1042	0.215	0.206	0.193	-0.0784	0.212	0.190	0.377	-0.0416	0.220	0.182	0.560	0.0121	0.271	0.258	0.938
	0.4	-0.1218	0.200	0.197	0.220	-0.0833	0.196	0.170	0.414	-0.0339	0.203	0.155	0.597	0.0625	0.255	0.242	0.952
	0.6	-0.1322	0.190	0.192	0.240	-0.0858	0.186	0.159	0.433	-0.0291	0.192	0.138	0.618	0.0995	0.237	0.230	0.957
	0.8	-0.1395	0.183	0.188	0.248	-0.0874	0.180	0.151	0.445	-0.0261	0.184	0.127	0.629	0.1321	0.217	0.218	0.960
	1	-0.1452	0.176	0.187	0.260	-0.0887	0.175	0.146	0.454	-0.0236	0.177	0.118	0.639	0.1696	0.182	0.203	0.963
0.6	0	-0.0892	0.231	0.234	0.163	-0.0938	0.235	0.242	0.336	-0.0926	0.243	0.248	0.562	-0.1145	0.275	0.287	0.950
	0.1	-0.1120	0.213	0.216	0.204	-0.0978	0.214	0.209	0.408	-0.0739	0.224	0.206	0.605	-0.0586	0.271	0.269	0.961
	0.2	-0.1240	0.203	0.208	0.236	-0.0989	0.201	0.192	0.445	-0.0642	0.211	0.183	0.642	-0.0218	0.265	0.257	0.970
	0.4	-0.1374	0.189	0.200	0.270	-0.1001	0.187	0.174	0.479	-0.0532	0.195	0.157	0.676	0.0296	0.251	0.238	0.979
	0.6	-0.1450	0.181	0.197	0.289	-0.1007	0.179	0.164	0.501	-0.0472	0.186	0.141	0.692	0.0683	0.235	0.222	0.981
	0.8	-0.1501	0.175	0.195	0.296	-0.1011	0.174	0.158	0.509	-0.0428	0.179	0.130	0.700	0.1031	0.215	0.205	0.981
	1	-0.1539	0.170	0.193	0.304	-0.1013	0.170	0.154	0.519	-0.0397	0.173	0.121	0.707	0.1448	0.177	0.182	0.983
1	0	-0.1108	0.221	0.238	0.194	-0.1162	0.226	0.246	0.400	-0.1158	0.235	0.253	0.628	-0.1391	0.268	0.293	0.971
	0.1	-0.1294	0.204	0.221	0.246	-0.1158	0.206	0.215	0.467	-0.0936	0.217	0.212	0.671	-0.0851	0.265	0.275	0.979
	0.2	-0.1389	0.194	0.213	0.278	-0.1149	0.195	0.198	0.499	-0.0820	0.205	0.189	0.703	-0.0488	0.261	0.261	0.984
	0.4	-0.1497	0.182	0.207	0.313	-0.1136	0.182	0.181	0.535	-0.0690	0.191	0.163	0.731	0.0027	0.248	0.240	0.986
	0.6	-0.1554	0.175	0.204	0.328	-0.1130	0.175	0.172	0.556	-0.0618	0.182	0.147	0.743	0.0423	0.233	0.220	0.988
	0.8	-0.1592	0.170	0.202	0.338	-0.1124	0.170	0.166	0.568	-0.0567	0.175	0.136	0.756	0.0790	0.213	0.200	0.988
	1	-0.1621	0.166	0.200	0.346	-0.1120	0.166	0.162	0.574	-0.0528	0.169	0.127	0.758	0.1251	0.173	0.167	0.989

Table A10. Impact of α_0 and ω on the operating characteristics for scenarios S1 to S4 (Weibull distribution for historical and new data, commensurate control arm), with σ_H^2 equivalent to 22 events.

Commensurate control arm																	
Parameters \ Treatment effect		S1: Null scenario HR = 1				S2: Disappointing effect scenario HR = 0.886				S3: Historical effect scenario HR = 0.786				S4: Anticipated effect scenario, HR = 0.55			
α_0	ω	Bias	SD	RMSE	Alpha	Bias	SD	RMSE	Power	Bias	SD	RMSE	Power	Bias	SD	RMSE	Power
0	0	0.0024	0.263	0.262	0.100	0.0015	0.268	0.267	0.208	0.0022	0.275	0.275	0.359	-0.0075	0.304	0.298	0.794
	0.1	-0.0171	0.253	0.244	0.108	-0.0048	0.252	0.244	0.213	0.0021	0.256	0.247	0.375	0.0170	0.281	0.277	0.809
	0.2	-0.0292	0.246	0.233	0.112	-0.0111	0.245	0.230	0.220	0.0020	0.248	0.232	0.384	0.0371	0.274	0.266	0.817
	0.4	-0.0439	0.238	0.220	0.117	-0.0186	0.236	0.215	0.229	0.0016	0.239	0.215	0.396	0.0632	0.262	0.250	0.823
	0.6	-0.0527	0.232	0.212	0.118	-0.0229	0.231	0.205	0.232	0.0014	0.233	0.206	0.401	0.0803	0.253	0.239	0.826
	0.8	-0.0588	0.227	0.206	0.121	-0.0259	0.227	0.199	0.237	0.0013	0.230	0.199	0.405	0.0929	0.245	0.230	0.828
	1	-0.0630	0.224	0.201	0.122	-0.0280	0.224	0.195	0.240	0.0011	0.227	0.194	0.407	0.1034	0.237	0.223	0.829
0.3	0	-0.0058	0.232	0.214	0.084	-0.0071	0.238	0.220	0.211	-0.0070	0.245	0.231	0.396	-0.0158	0.274	0.259	0.883
	0.1	-0.0225	0.225	0.201	0.092	-0.0120	0.227	0.203	0.215	-0.0069	0.231	0.208	0.415	0.0069	0.258	0.240	0.896
	0.2	-0.0324	0.220	0.194	0.097	-0.0166	0.221	0.194	0.225	-0.0069	0.225	0.197	0.425	0.0262	0.252	0.231	0.900
	0.4	-0.0432	0.214	0.186	0.101	-0.0217	0.215	0.183	0.233	-0.0060	0.218	0.185	0.436	0.0505	0.242	0.218	0.905
	0.6	-0.0494	0.210	0.181	0.104	-0.0245	0.211	0.178	0.237	-0.0052	0.214	0.179	0.447	0.0658	0.234	0.208	0.907
	0.8	-0.0535	0.207	0.178	0.107	-0.0262	0.209	0.174	0.242	-0.0048	0.212	0.174	0.445	0.0768	0.228	0.201	0.909
	1	-0.0564	0.205	0.176	0.109	-0.0275	0.207	0.171	0.242	-0.0046	0.210	0.171	0.452	0.0857	0.221	0.195	0.911
0.6	0	-0.0095	0.219	0.200	0.082	-0.0108	0.225	0.206	0.222	-0.0111	0.233	0.218	0.418	-0.0199	0.261	0.248	0.911
	0.1	-0.0246	0.213	0.190	0.090	-0.0147	0.215	0.191	0.224	-0.0110	0.220	0.198	0.443	0.0022	0.248	0.230	0.926
	0.2	-0.0332	0.208	0.184	0.097	-0.0188	0.210	0.183	0.235	-0.0100	0.215	0.188	0.454	0.0206	0.242	0.220	0.931
	0.4	-0.0428	0.203	0.177	0.101	-0.0230	0.205	0.174	0.244	-0.0084	0.209	0.177	0.466	0.0440	0.233	0.208	0.934
	0.6	-0.0480	0.200	0.173	0.103	-0.0252	0.202	0.170	0.249	-0.0076	0.205	0.171	0.475	0.0587	0.226	0.199	0.937
	0.8	-0.0514	0.198	0.171	0.106	-0.0267	0.200	0.167	0.252	-0.0069	0.203	0.168	0.476	0.0691	0.220	0.192	0.938
	1	-0.0538	0.196	0.169	0.106	-0.0276	0.199	0.165	0.252	-0.0066	0.202	0.165	0.477	0.0772	0.214	0.187	0.939
1	0	-0.0121	0.210	0.193	0.087	-0.0137	0.216	0.200	0.233	-0.0141	0.223	0.212	0.448	-0.0230	0.252	0.242	0.929
	0.1	-0.0260	0.204	0.184	0.095	-0.0167	0.207	0.186	0.236	-0.0136	0.212	0.192	0.467	-0.0015	0.241	0.225	0.944
	0.2	-0.0337	0.200	0.179	0.100	-0.0202	0.203	0.178	0.245	-0.0121	0.207	0.183	0.479	0.0166	0.235	0.216	0.947
	0.4	-0.0428	0.196	0.173	0.104	-0.0239	0.198	0.170	0.256	-0.0102	0.202	0.173	0.491	0.0390	0.227	0.203	0.949
	0.6	-0.0474	0.193	0.170	0.106	-0.0258	0.195	0.166	0.259	-0.0092	0.199	0.168	0.495	0.0533	0.220	0.194	0.953
	0.8	-0.0504	0.191	0.167	0.108	-0.0270	0.194	0.164	0.262	-0.0087	0.197	0.165	0.500	0.0633	0.214	0.188	0.952
	1	-0.0525	0.190	0.166	0.111	-0.0279	0.192	0.162	0.264	-0.0083	0.195	0.163	0.502	0.0710	0.208	0.182	0.954

Table A11. Impact of α_0 and ω on the operating characteristics for scenarios S5 to S8 (Weibull distribution for historical and new data, negative prior-data conflict), with σ_H^2 equivalent to 22 events.

		Control arm: negative prior-data conflict															
Parameters	Treatment effect	S5: Null scenario HR = 1				S6: Disappointing effect scenario HR = 0.886				S7: Historical effect scenario HR = 0.786				S8: Anticipated effect scenario, HR = 0.55			
α_0	ω	Bias	SD	RMSE	Alpha	Bias	SD	RMSE	Power	Bias	SD	RMSE	Power	Bias	SD	RMSE	Power
0	0	0.0003	0.241	0.243	0.104	0.0008	0.240	0.243	0.224	0.0036	0.245	0.247	0.382	-0.0024	0.263	0.264	0.849
	0.1	-0.0174	0.233	0.228	0.110	-0.0075	0.231	0.225	0.229	0.0040	0.236	0.227	0.405	0.0217	0.256	0.251	0.858
	0.2	-0.0283	0.227	0.219	0.117	-0.0132	0.225	0.214	0.237	0.0040	0.230	0.216	0.414	0.0394	0.250	0.242	0.864
	0.4	-0.0411	0.221	0.208	0.122	-0.0196	0.219	0.202	0.246	0.0036	0.223	0.202	0.424	0.0613	0.241	0.231	0.870
	0.6	-0.0486	0.216	0.201	0.124	-0.0233	0.214	0.195	0.254	0.0037	0.218	0.195	0.430	0.0753	0.234	0.223	0.873
	0.8	-0.0536	0.213	0.197	0.127	-0.0258	0.212	0.190	0.255	0.0038	0.216	0.190	0.432	0.0853	0.228	0.217	0.877
	1	-0.0572	0.210	0.194	0.128	-0.0272	0.210	0.187	0.259	0.0038	0.214	0.186	0.436	0.0937	0.223	0.211	0.874
0.3	0	0.0798	0.216	0.213	0.038	0.0755	0.217	0.213	0.115	0.0619	0.223	0.221	0.265	0.0813	0.243	0.239	0.841
	0.1	0.0576	0.211	0.196	0.037	0.0662	0.211	0.198	0.120	0.0577	0.216	0.206	0.283	0.1003	0.235	0.233	0.852
	0.2	0.0457	0.208	0.186	0.041	0.0585	0.207	0.188	0.127	0.0554	0.211	0.196	0.291	0.1135	0.229	0.231	0.861
	0.4	0.0320	0.202	0.175	0.042	0.0501	0.201	0.177	0.131	0.0530	0.205	0.187	0.300	0.1289	0.221	0.228	0.865
	0.6	0.0241	0.199	0.167	0.044	0.0454	0.198	0.171	0.134	0.0518	0.202	0.182	0.308	0.1379	0.216	0.226	0.868
	0.8	0.0188	0.196	0.162	0.044	0.0425	0.196	0.167	0.137	0.0510	0.200	0.178	0.310	0.1442	0.212	0.224	0.871
	1	0.0151	0.194	0.159	0.045	0.0404	0.195	0.164	0.137	0.0500	0.199	0.174	0.312	0.1484	0.209	0.223	0.873
0.6	0	0.1150	0.205	0.216	0.020	0.1124	0.206	0.216	0.075	0.1169	0.212	0.224	0.206	0.1212	0.233	0.245	0.824
	0.1	0.0928	0.201	0.198	0.021	0.1017	0.201	0.202	0.079	0.1095	0.206	0.209	0.222	0.1377	0.224	0.242	0.838
	0.2	0.0809	0.198	0.188	0.022	0.0934	0.197	0.192	0.083	0.1055	0.202	0.201	0.231	0.1487	0.219	0.241	0.844
	0.4	0.0670	0.194	0.175	0.023	0.0843	0.193	0.181	0.090	0.1013	0.197	0.191	0.241	0.1611	0.212	0.239	0.849
	0.6	0.0589	0.190	0.167	0.024	0.0794	0.190	0.175	0.092	0.0991	0.194	0.187	0.244	0.1683	0.208	0.239	0.854
	0.8	0.0537	0.188	0.162	0.025	0.0762	0.189	0.171	0.092	0.0977	0.193	0.184	0.248	0.1729	0.204	0.238	0.855
	1	0.0498	0.186	0.158	0.024	0.0740	0.187	0.168	0.093	0.0968	0.191	0.182	0.252	0.1763	0.202	0.238	0.857
1	0	0.1407	0.196	0.224	0.014	0.1395	0.198	0.225	0.056	0.1448	0.204	0.233	0.168	0.1503	0.225	0.255	0.811
	0.1	0.1189	0.193	0.206	0.013	0.1280	0.194	0.211	0.059	0.1361	0.198	0.219	0.182	0.1650	0.217	0.253	0.822
	0.2	0.1071	0.191	0.195	0.015	0.1195	0.191	0.202	0.062	0.1315	0.195	0.211	0.190	0.1744	0.212	0.253	0.828
	0.4	0.0929	0.187	0.182	0.016	0.1101	0.187	0.191	0.065	0.1266	0.191	0.202	0.196	0.1851	0.205	0.253	0.833
	0.6	0.0848	0.184	0.174	0.016	0.1049	0.184	0.184	0.068	0.1240	0.188	0.198	0.202	0.1909	0.202	0.253	0.837
	0.8	0.0796	0.182	0.168	0.017	0.1016	0.183	0.180	0.070	0.1225	0.187	0.195	0.204	0.1949	0.199	0.253	0.840
	1	0.0756	0.183	0.164	0.017	0.0993	0.181	0.177	0.070	0.1212	0.186	0.193	0.206	0.1976	0.197	0.253	0.841

Table A12. Impact of α_0 and ω on the operating characteristics for scenarios S9 to S12 (Weibull distribution for historical and new data, positive prior-data conflict), with σ_H^2 equivalent to 22 events.

Parameters \ Treatment effect		Control arm: positive prior-data conflict															
		S9: Null scenario HR = 1				S10: Disappointing effect scenario HR = 0.886				S11: Historical effect scenario HR = 0.786				S12: Anticipated effect scenario, HR = 0.55			
α_0	ω	Bias	SD	RMSE	Alpha	Bias	SD	RMSE	Power	Bias	SD	RMSE	Power	Bias	SD	RMSE	Power
0	0	0.0009	0.281	0.280	0.102	-0.0003	0.280	0.284	0.201	0.0008	0.286	0.291	0.354	-0.0089	0.315	0.315	0.764
	0.1	-0.0197	0.270	0.260	0.109	-0.0113	0.268	0.259	0.217	0.0007	0.274	0.263	0.368	0.0182	0.303	0.296	0.770
	0.2	-0.0327	0.262	0.247	0.112	-0.0180	0.259	0.244	0.223	0.0006	0.265	0.247	0.378	0.0398	0.295	0.282	0.777
	0.4	-0.0489	0.252	0.232	0.117	-0.0259	0.249	0.225	0.232	0.0006	0.254	0.226	0.387	0.0689	0.281	0.264	0.783
	0.6	-0.0586	0.245	0.222	0.121	-0.0306	0.242	0.214	0.235	0.0006	0.247	0.214	0.392	0.0881	0.270	0.251	0.788
	0.8	-0.0655	0.239	0.215	0.124	-0.0338	0.238	0.206	0.235	0.0005	0.242	0.205	0.398	0.1028	0.260	0.241	0.791
	1	-0.0706	0.235	0.209	0.125	-0.0363	0.234	0.201	0.238	0.0003	0.239	0.199	0.404	0.1153	0.250	0.232	0.793
0.3	0	-0.0605	0.246	0.237	0.132	-0.0640	0.248	0.244	0.274	-0.0616	0.256	0.250	0.481	-0.0817	0.287	0.286	0.905
	0.1	-0.0739	0.236	0.224	0.141	-0.0677	0.237	0.225	0.291	-0.0599	0.246	0.230	0.484	-0.0489	0.279	0.266	0.909
	0.2	-0.0820	0.230	0.218	0.147	-0.0697	0.231	0.215	0.299	-0.0558	0.239	0.217	0.495	-0.0256	0.272	0.251	0.914
	0.4	-0.0907	0.223	0.210	0.154	-0.0719	0.223	0.203	0.309	-0.0508	0.230	0.201	0.504	0.0051	0.261	0.230	0.918
	0.6	-0.0957	0.219	0.206	0.157	-0.0732	0.219	0.197	0.317	-0.0481	0.225	0.193	0.510	0.0259	0.251	0.214	0.920
	0.8	-0.0990	0.216	0.203	0.159	-0.0740	0.216	0.193	0.320	-0.0463	0.222	0.187	0.515	0.0416	0.242	0.200	0.921
	1	-0.1012	0.214	0.201	0.162	-0.0746	0.214	0.191	0.321	-0.0451	0.220	0.183	0.517	0.0547	0.232	0.188	0.923
0.6	0	-0.0892	0.231	0.234	0.163	-0.0938	0.235	0.242	0.336	-0.0926	0.243	0.248	0.562	-0.1145	0.275	0.287	0.950
	0.1	-0.0996	0.223	0.224	0.174	-0.0944	0.225	0.225	0.357	-0.0878	0.234	0.230	0.566	-0.0821	0.269	0.266	0.951
	0.2	-0.1053	0.218	0.218	0.182	-0.0949	0.220	0.216	0.366	-0.0823	0.228	0.217	0.573	-0.0585	0.263	0.250	0.954
	0.4	-0.1116	0.212	0.213	0.185	-0.0949	0.213	0.206	0.379	-0.0757	0.221	0.203	0.585	-0.0273	0.252	0.227	0.958
	0.6	-0.1151	0.208	0.210	0.191	-0.0951	0.209	0.201	0.385	-0.0724	0.216	0.194	0.594	-0.0061	0.243	0.208	0.958
	0.8	-0.1173	0.206	0.208	0.195	-0.0951	0.207	0.198	0.387	-0.0700	0.213	0.189	0.594	0.0102	0.234	0.192	0.958
	1	-0.1189	0.204	0.206	0.194	-0.0954	0.206	0.195	0.392	-0.0684	0.211	0.185	0.596	0.0240	0.223	0.177	0.960
1	0	-0.1108	0.221	0.238	0.194	-0.1162	0.226	0.246	0.400	-0.1158	0.235	0.253	0.628	-0.1391	0.268	0.293	0.971
	0.1	-0.1189	0.214	0.229	0.208	-0.1148	0.217	0.231	0.415	-0.1092	0.226	0.235	0.632	-0.1073	0.262	0.272	0.973
	0.2	-0.1232	0.209	0.224	0.219	-0.1139	0.212	0.222	0.425	-0.1028	0.221	0.223	0.640	-0.0843	0.257	0.256	0.973
	0.4	-0.1282	0.204	0.219	0.225	-0.1129	0.206	0.213	0.434	-0.0956	0.214	0.209	0.650	-0.0525	0.247	0.230	0.975
	0.6	-0.1308	0.201	0.217	0.231	-0.1124	0.203	0.208	0.439	-0.0914	0.210	0.200	0.657	-0.0309	0.237	0.209	0.976
	0.8	-0.1324	0.199	0.215	0.231	-0.1120	0.201	0.204	0.443	-0.0887	0.207	0.195	0.661	-0.0140	0.228	0.191	0.977
	1	-0.1334	0.197	0.214	0.232	-0.1118	0.199	0.202	0.448	-0.0868	0.205	0.191	0.665	0.0004	0.218	0.173	0.977
