# Supplementary material for: Incorporating individual historical controls and aggregate treatment effect estimates into a Bayesian survival trial: a simulation study
Source: BMC Med Res Methodol. 2019 Apr 24;19:85. doi: 10.1186/s12874-019-0714-z (PMC6480797; doi:10.1186/s12874-019-0714-z)
Supplement: Supplementary file 11 — Figure A7. Posterior distribution of the log-hazard ratio depending on the values of the weighting parameters. This figure represents no incorporation of historical data (solid black curve), weighted incorporation of historical data (dashed red curve), and full incorporation of historical data (dot dashed blue curve). (PDF 117 kb) [file 12874_2019_714_MOESM11_ESM.pdf]

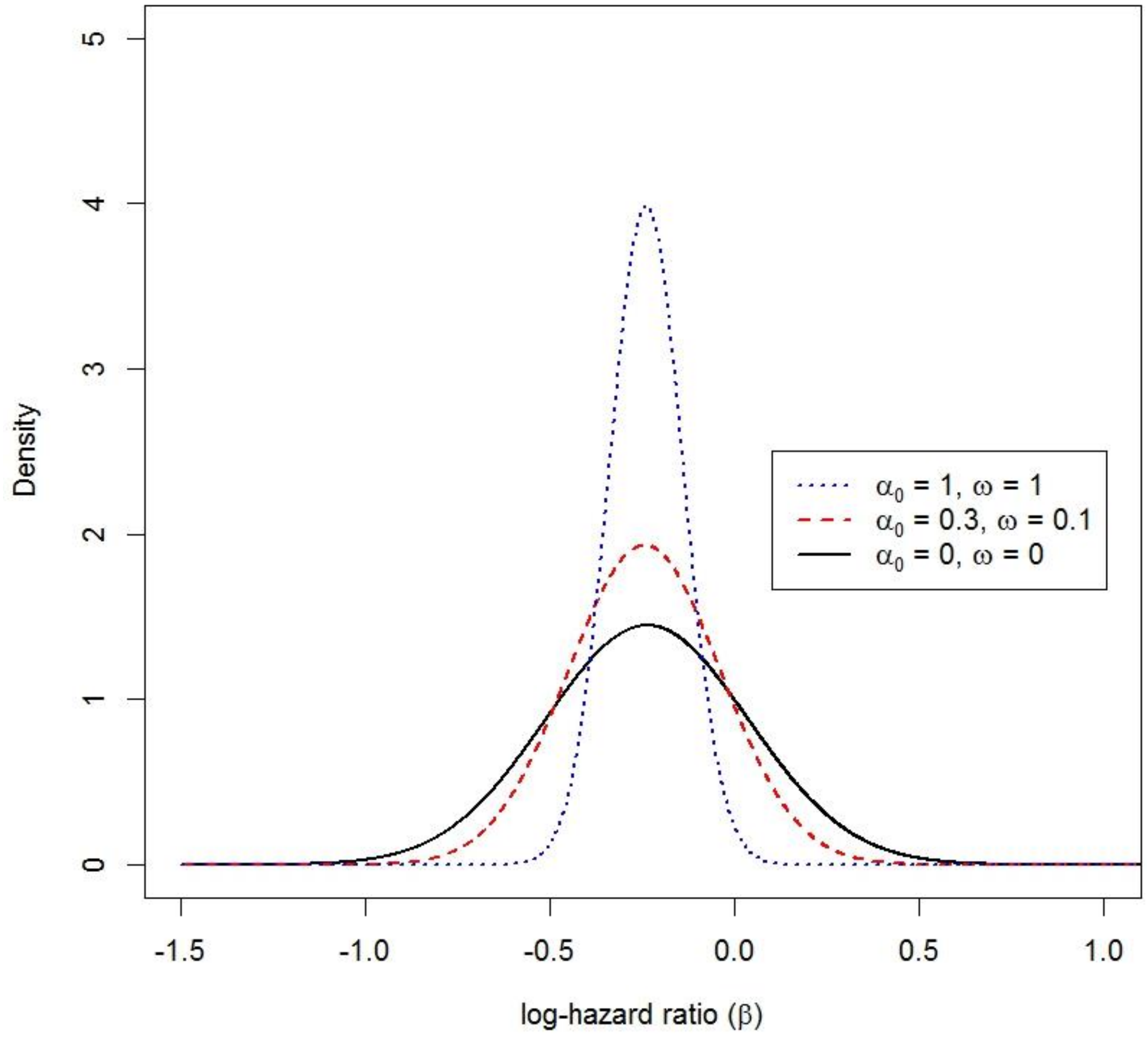

Figure A7: Posterior distribution of the log-hazard ratio depending on the values of the weighting parameters.

The figure represents no incorporation of historical data (solid black curve), weighted incorporation of historical data (dashed red curve), and full incorporation of historical data (dot dashed blue curve).
