# Supplementary material for: Incorporating individual historical controls and aggregate treatment effect estimates into a Bayesian survival trial: a simulation study
Source: BMC Med Res Methodol. 2019 Apr 24;19:85. doi: 10.1186/s12874-019-0714-z (PMC6480797; doi:10.1186/s12874-019-0714-z)
Supplement: Supplementary file 12 — Tables A13-A16. Impact of α0 and ω on the operating characteristics for scenarios S25 to S32 when there is a conflict in terms of the underlying survival distribution between historical and new data. These tables describe the impact of α0 and ω on the operating characteristics in case of a conflict in terms of survival distribution between historical and new data. Tables A13 and A14 describe results for scenarios S25 to S28 when the historical data follow a Weibull distribution, the new data follow a piecewise exponential distribution, and data are analysed using either a Bayesian Weibull model (Table A13) or a Bayesian piecewise exponential model (Table A14). Tables A15 and A16 describe results for scenarios S29 to S32 when the historical data follow a piecewise exponential distribution, the new data follow a Weibull distribution, and data are analysed using either a Bayesian piecewise exponential model (Table A15) or a Bayesian Weibull model (Table A16). (PDF 554 kb) [file 12874_2019_714_MOESM12_ESM.pdf]

Table A13. Impact of α_0 and ω on the operating characteristics for scenarios S25 to S28 (Weibull distribution for historical data / piecewise exponential distribution for new data) with a Bayesian Weibull analysis (W/P/W)

Historical data: Weibull survival distribution / New data: piecewise exponential distribution / Bayesian Weibull analysis (W/P/W)																	
Treatment effect / Parameters		S25: Null scenario HR = 1				S26: Disappointing effect scenario HR = 0.886				S27: Historical effect scenario HR = 0.786				S28: Anticipated effect scenario, HR = 0.55			
α_0	ω	Bias	SD	RMSE	Alpha	Bias	SD	RMSE	Power	Bias	SD	RMSE	Power	Bias	SD	RMSE	Power
0	0	-0.0015	0.277	0.287	0.113	-0.0088	0.284	0.294	0.220	-0.0142	0.292	0.298	0.358	-0.0344	0.326	0.327	0.776
	0.1	-0.0497	0.253	0.254	0.223	-0.0332	0.249	0.236	0.370	-0.0109	0.252	0.232	0.521	0.0315	0.309	0.313	0.880
	0.2	-0.0788	0.234	0.240	0.331	-0.0477	0.224	0.207	0.491	-0.0085	0.225	0.195	0.650	0.0745	0.292	0.308	0.932
	0.4	-0.1179	0.204	0.226	0.507	-0.0667	0.190	0.170	0.676	-0.0063	0.188	0.148	0.809	0.1355	0.262	0.306	0.978
	0.6	-0.1457	0.179	0.220	0.649	-0.0795	0.163	0.147	0.796	-0.0041	0.161	0.114	0.894	0.1834	0.233	0.307	0.991
	0.8	-0.1716	0.153	0.215	0.791	-0.0910	0.139	0.129	0.893	-0.0028	0.138	0.082	0.953	0.2304	0.199	0.310	0.997
	1	-0.2071	0.106	0.211	0.967	-0.1045	0.107	0.112	0.992	-0.0016	0.108	0.039	0.997	0.3125	0.112	0.315	1
0.3	0	-0.0387	0.243	0.238	0.120	-0.0472	0.250	0.248	0.262	-0.0541	0.258	0.255	0.447	-0.0784	0.292	0.294	0.893
	0.1	-0.0849	0.219	0.215	0.259	-0.0652	0.214	0.196	0.447	-0.0394	0.220	0.193	0.638	-0.0076	0.285	0.287	0.958
	0.2	-0.1108	0.201	0.208	0.377	-0.0747	0.191	0.170	0.577	-0.0311	0.195	0.159	0.760	0.0381	0.275	0.286	0.982
	0.4	-0.1429	0.175	0.204	0.544	-0.0863	0.162	0.144	0.750	-0.0214	0.164	0.119	0.888	0.1035	0.253	0.288	0.995
	0.6	-0.1648	0.153	0.204	0.694	-0.0936	0.140	0.130	0.856	-0.0153	0.142	0.092	0.942	0.1558	0.229	0.291	0.999
	0.8	-0.1830	0.133	0.205	0.816	-0.0993	0.123	0.120	0.929	-0.0106	0.124	0.066	0.977	0.2072	0.197	0.294	1
	1	-0.2042	0.103	0.208	0.966	-0.1054	0.104	0.113	0.992	-0.0061	0.104	0.040	0.998	0.2983	0.107	0.301	1
0.6	0	-0.0563	0.229	0.228	0.138	-0.0656	0.236	0.239	0.300	-0.0736	0.245	0.248	0.505	-0.1001	0.278	0.290	0.932
	0.1	-0.0994	0.205	0.209	0.286	-0.0790	0.201	0.189	0.495	-0.0530	0.208	0.187	0.700	-0.0310	0.276	0.285	0.976
	0.2	-0.1230	0.188	0.203	0.407	-0.0857	0.179	0.166	0.629	-0.0420	0.185	0.156	0.809	0.0144	0.269	0.284	0.990
	0.4	-0.1513	0.163	0.201	0.583	-0.0938	0.152	0.142	0.781	-0.0293	0.157	0.118	0.910	0.0797	0.252	0.284	0.998
	0.6	-0.1703	0.144	0.202	0.715	-0.0985	0.133	0.129	0.873	-0.0212	0.137	0.091	0.957	0.1331	0.230	0.287	1
	0.8	-0.1859	0.127	0.204	0.829	-0.1026	0.118	0.121	0.939	-0.0149	0.121	0.067	0.982	0.1878	0.200	0.289	1
	1	-0.2033	0.101	0.208	0.961	-0.1067	0.102	0.115	0.991	-0.0094	0.102	0.041	0.999	0.2898	0.104	0.292	1
1	0	-0.0695	0.219	0.226	0.153	-0.0796	0.227	0.238	0.340	-0.0883	0.235	0.247	0.554	-0.1168	0.268	0.291	0.953
	0.1	-0.1092	0.195	0.208	0.310	-0.0888	0.193	0.189	0.537	-0.0633	0.201	0.188	0.742	-0.0500	0.270	0.286	0.985
	0.2	-0.1306	0.179	0.203	0.433	-0.0935	0.172	0.166	0.661	-0.0503	0.180	0.157	0.839	-0.0058	0.265	0.285	0.994
	0.4	-0.1573	0.156	0.202	0.601	-0.0995	0.146	0.144	0.804	-0.0356	0.152	0.120	0.929	0.0585	0.251	0.285	0.998
	0.6	-0.1741	0.138	0.203	0.734	-0.1027	0.129	0.131	0.889	-0.0264	0.134	0.094	0.965	0.1125	0.231	0.286	0.999
	0.8	-0.1877	0.123	0.205	0.839	-0.1051	0.115	0.123	0.945	-0.0193	0.119	0.071	0.986	0.1688	0.203	0.286	1
	1	-0.2031	0.100	0.208	0.957	-0.1081	0.101	0.116	0.990	-0.0123	0.101	0.043	0.998	0.2826	0.102	0.285	1

Table A14. Impact of α_0 and ω on the operating characteristics for scenarios S25 to S28 (Weibull distribution for historical data / piecewise exponential distribution for new data) with a Bayesian piecewise exponential analysis (W/P/P)

Historical data: Weibull survival distribution / New data: piecewise exponential distribution / Bayesian piecewise exponential analysis (W/P/P)																	
Parameters \ Treatment effect		S25: Null scenario HR = 1				S26: Disappointing effect scenario HR = 0.886				S27: Historical effect scenario HR = 0.786				S28: Anticipated effect scenario HR = 0.55			
α_0	ω	Bias	SD	RMSE	Alpha	Bias	SD	RMSE	Power	Bias	SD	RMSE	Power	Bias	SD	RMSE	Power
0	0	-0.0014	0.274	0.280	0.104	-0.0056	0.280	0.287	0.212	-0.0087	0.287	0.292	0.345	-0.0230	0.313	0.321	0.766
	0.1	-0.0510	0.249	0.248	0.217	-0.0316	0.244	0.230	0.366	-0.0065	0.247	0.226	0.516	0.0429	0.298	0.306	0.875
	0.2	-0.0810	0.230	0.235	0.327	-0.0468	0.220	0.200	0.486	-0.0047	0.220	0.188	0.643	0.0859	0.283	0.302	0.932
	0.4	-0.1202	0.200	0.222	0.508	-0.0662	0.185	0.165	0.670	-0.0034	0.183	0.142	0.810	0.1462	0.254	0.302	0.978
	0.6	-0.1481	0.175	0.216	0.653	-0.0793	0.159	0.143	0.798	-0.0021	0.155	0.109	0.897	0.1932	0.225	0.305	0.991
	0.8	-0.1732	0.148	0.213	0.794	-0.0906	0.134	0.127	0.897	-0.0009	0.131	0.078	0.953	0.2388	0.191	0.309	0.998
	1	-0.2070	0.102	0.211	0.972	-0.1040	0.102	0.111	0.992	-0.0005	0.103	0.038	0.998	0.3139	0.104	0.316	1
0.3	0	-0.0255	0.241	0.231	0.100	-0.0317	0.248	0.241	0.240	-0.0366	0.256	0.248	0.416	-0.0556	0.285	0.285	0.874
	0.1	-0.0750	0.218	0.209	0.234	-0.0540	0.213	0.188	0.417	-0.0262	0.216	0.185	0.618	0.0158	0.278	0.279	0.956
	0.2	-0.1028	0.200	0.202	0.348	-0.0660	0.190	0.164	0.553	-0.0203	0.191	0.152	0.740	0.0613	0.268	0.280	0.979
	0.4	-0.1375	0.174	0.199	0.529	-0.0801	0.159	0.139	0.731	-0.0137	0.160	0.113	0.874	0.1246	0.245	0.284	0.995
	0.6	-0.1604	0.153	0.200	0.676	-0.0887	0.138	0.125	0.841	-0.0092	0.138	0.085	0.938	0.1742	0.221	0.288	0.999
	0.8	-0.1800	0.132	0.202	0.809	-0.0959	0.120	0.117	0.923	-0.0061	0.120	0.063	0.976	0.2223	0.189	0.294	1
	1	-0.2020	0.101	0.206	0.966	-0.1028	0.101	0.110	0.992	-0.0030	0.101	0.038	0.998	0.3018	0.102	0.304	1
0.6	0	-0.0410	0.228	0.221	0.114	-0.0483	0.235	0.232	0.268	-0.0544	0.243	0.240	0.464	-0.0766	0.273	0.281	0.919
	0.1	-0.0873	0.205	0.202	0.256	-0.0663	0.200	0.182	0.462	-0.0385	0.206	0.179	0.669	-0.0060	0.271	0.277	0.973
	0.2	-0.1133	0.188	0.198	0.376	-0.0760	0.179	0.160	0.597	-0.0304	0.183	0.148	0.785	0.0391	0.264	0.277	0.989
	0.4	-0.1442	0.164	0.197	0.554	-0.0865	0.151	0.137	0.759	-0.0204	0.153	0.111	0.902	0.1030	0.245	0.280	0.997
	0.6	-0.1649	0.145	0.198	0.696	-0.0934	0.132	0.125	0.863	-0.0147	0.134	0.085	0.951	0.1540	0.223	0.284	0.999
	0.8	-0.1818	0.126	0.201	0.816	-0.0983	0.116	0.117	0.932	-0.0097	0.117	0.063	0.979	0.2045	0.193	0.289	1
	1	-0.2006	0.100	0.205	0.960	-0.1036	0.100	0.111	0.990	-0.0058	0.100	0.040	0.998	0.2938	0.101	0.296	1
1	0	-0.0546	0.219	0.219	0.133	-0.0630	0.226	0.230	0.305	-0.0702	0.235	0.239	0.518	-0.0949	0.265	0.282	0.941
	0.1	-0.0979	0.196	0.202	0.282	-0.0769	0.192	0.182	0.499	-0.0497	0.199	0.180	0.715	-0.0265	0.266	0.279	0.982
	0.2	-0.1214	0.180	0.198	0.400	-0.0841	0.171	0.160	0.627	-0.0392	0.177	0.149	0.821	0.0177	0.261	0.279	0.993
	0.4	-0.1498	0.157	0.197	0.576	-0.0924	0.145	0.138	0.785	-0.0274	0.150	0.114	0.918	0.0813	0.245	0.281	0.998
	0.6	-0.1687	0.139	0.199	0.713	-0.0972	0.128	0.126	0.878	-0.0196	0.131	0.088	0.960	0.1333	0.225	0.283	1
	0.8	-0.1836	0.122	0.201	0.823	-0.1010	0.114	0.119	0.939	-0.0140	0.116	0.066	0.983	0.1862	0.197	0.286	1
	1	-0.2003	0.099	0.205	0.953	-0.1050	0.099	0.113	0.988	-0.0088	0.099	0.042	0.998	0.2867	0.100	0.289	1

Table A15. Impact of α_0 and ω on the operating characteristics for scenarios S29 to S32 (piecewise exponential distribution for historical data / Weibull survival distribution for new data) with a Bayesian piecewise exponential analysis (P/W/P)

Historical data: piecewise exponential distribution / New data: Weibull survival distribution / Bayesian piecewise exponential analysis (P/W/P)																	
Parameters \ Treatment effect		S29: Null scenario HR = 1				S30: Disappointing effect scenario HR = 0.886				S31: Historical effect scenario HR = 0.786				S32: Anticipated effect scenario HR = 0.55			
α_0	ω	Bias	SD	RMSE	Alpha	Bias	SD	RMSE	Power	Bias	SD	RMSE	Power	Bias	SD	RMSE	Power
0	0	0.0031	0.260	0.261	0.099	0	0.265	0.269	0.210	-0.0032	0.270	0.274	0.355	-0.0161	0.292	0.295	0.798
	0.1	-0.0493	0.237	0.234	0.203	-0.0282	0.231	0.215	0.373	-0.0021	0.232	0.209	0.550	0.0487	0.280	0.286	0.903
	0.2	-0.0796	0.220	0.224	0.315	-0.0440	0.208	0.188	0.503	-0.0016	0.207	0.174	0.667	0.0904	0.267	0.284	0.951
	0.4	-0.1189	0.192	0.215	0.501	-0.0639	0.176	0.157	0.680	-0.0012	0.173	0.131	0.805	0.1486	0.242	0.287	0.983
	0.6	-0.1465	0.168	0.211	0.653	-0.0776	0.152	0.138	0.798	-0.0010	0.148	0.101	0.887	0.1938	0.216	0.292	0.994
	0.8	-0.1707	0.145	0.209	0.786	-0.0889	0.130	0.123	0.887	-0.0010	0.127	0.074	0.955	0.2378	0.185	0.298	0.999
	1	-0.2033	0.101	0.207	0.966	-0.1020	0.102	0.110	0.990	0	0.102	0.040	0.998	0.3090	0.103	0.311	1
0.3	0	0.0040	0.230	0.210	0.070	0.0020	0.236	0.217	0.189	0.0005	0.242	0.224	0.378	-0.0051	0.267	0.250	0.873
	0.1	-0.0525	0.212	0.193	0.186	-0.0299	0.203	0.169	0.381	0.0012	0.203	0.163	0.591	0.0645	0.258	0.252	0.958
	0.2	-0.0838	0.197	0.189	0.292	-0.0466	0.182	0.148	0.516	0.0014	0.180	0.132	0.711	0.1072	0.246	0.257	0.982
	0.4	-0.1222	0.173	0.188	0.482	-0.0659	0.154	0.127	0.700	0.0016	0.150	0.097	0.852	0.1643	0.223	0.268	0.995
	0.6	-0.1478	0.153	0.190	0.638	-0.0780	0.135	0.115	0.823	0.0019	0.131	0.074	0.925	0.2068	0.199	0.278	0.999
	0.8	-0.1693	0.132	0.193	0.777	-0.0873	0.119	0.109	0.908	0.0022	0.116	0.056	0.971	0.2461	0.170	0.288	1
	1	-0.1940	0.100	0.198	0.951	-0.0961	0.100	0.104	0.988	0.0027	0.101	0.039	0.998	0.3030	0.101	0.305	1
0.6	0	0.0081	0.217	0.195	0.062	0.0075	0.224	0.201	0.180	0.0076	0.231	0.209	0.376	0.0056	0.256	0.236	0.894
	0.1	-0.0488	0.202	0.181	0.170	-0.0264	0.192	0.157	0.378	0.0066	0.192	0.150	0.599	0.0754	0.247	0.243	0.969
	0.2	-0.0801	0.189	0.179	0.280	-0.0437	0.173	0.138	0.518	0.0059	0.170	0.122	0.729	0.1173	0.236	0.251	0.987
	0.4	-0.1182	0.167	0.181	0.464	-0.0632	0.147	0.119	0.701	0.0052	0.143	0.089	0.862	0.1723	0.213	0.264	0.998
	0.6	-0.1437	0.148	0.184	0.618	-0.0750	0.130	0.110	0.820	0.0050	0.125	0.069	0.927	0.2126	0.190	0.275	0.999
	0.8	-0.1648	0.129	0.187	0.766	-0.0840	0.115	0.104	0.906	0.0048	0.112	0.053	0.969	0.2495	0.163	0.286	1
	1	-0.1885	0.099	0.193	0.940	-0.0922	0.099	0.100	0.983	0.0049	0.100	0.039	0.998	0.3009	0.100	0.303	1
1	0	0.0131	0.208	0.187	0.057	0.0138	0.215	0.192	0.174	0.0153	0.222	0.201	0.376	0.0158	0.249	0.230	0.907
	0.1	-0.0438	0.196	0.175	0.161	-0.0218	0.185	0.151	0.372	0.0123	0.184	0.145	0.607	0.0849	0.240	0.240	0.972
	0.2	-0.0748	0.184	0.174	0.266	-0.0397	0.167	0.133	0.513	0.0106	0.163	0.117	0.731	0.1259	0.228	0.249	0.989
	0.4	-0.1131	0.163	0.176	0.450	-0.0595	0.143	0.115	0.696	0.0090	0.138	0.086	0.861	0.1789	0.206	0.264	0.998
	0.6	-0.1387	0.146	0.179	0.595	-0.0717	0.127	0.107	0.815	0.0081	0.122	0.067	0.927	0.2174	0.184	0.275	0.999
	0.8	-0.1598	0.128	0.183	0.741	-0.0806	0.114	0.101	0.900	0.0075	0.110	0.053	0.970	0.2521	0.158	0.285	1
	1	-0.1840	0.099	0.188	0.922	-0.0889	0.099	0.098	0.979	0.0072	0.099	0.041	0.997	0.2999	0.100	0.302	1

Table A16. Impact of α_0 and ω on the operating characteristics for scenarios S29 to S32 (piecewise exponential distribution for historical data / Weibull survival distribution for new data) with a Bayesian Weibull analysis (P/W/W)

Historical data: piecewise exponential distribution / New data: Weibull survival distribution / Bayesian Weibull analysis (P/W/W)																	
Parameters \ Treatment effect		S29: Null scenario HR = 1				S30: Disappointing effect scenario HR = 0.886				S31: Historical effect scenario HR = 0.786				S32: Anticipated effect scenario HR = 0.55			
α_0	ω	Bias	SD	RMSE	Alpha	Bias	SD	RMSE	Power	Bias	SD	RMSE	Power	Bias	SD	RMSE	Power
0	0	0.0032	0.262	0.257	0.098	0.0009	0.268	0.266	0.208	0.0004	0.275	0.276	0.347	-0.0084	0.275	0.294	0.793
	0.1	-0.0498	0.239	0.231	0.204	-0.0273	0.233	0.212	0.376	-0.0010	0.235	0.211	0.529	0.0548	0.288	0.284	0.898
	0.2	-0.0807	0.222	0.221	0.313	-0.0436	0.210	0.185	0.500	-0.0007	0.210	0.176	0.658	0.0966	0.272	0.284	0.949
	0.4	-0.1199	0.194	0.212	0.499	-0.0637	0.178	0.154	0.684	-0.0007	0.176	0.133	0.806	0.1548	0.244	0.287	0.983
	0.6	-0.1476	0.171	0.209	0.652	-0.0776	0.155	0.135	0.798	-0.0006	0.151	0.102	0.898	0.1996	0.218	0.293	0.995
	0.8	-0.1714	0.147	0.208	0.788	-0.0890	0.133	0.122	0.889	-0.0003	0.131	0.074	0.959	0.2422	0.186	0.299	0.999
	1	-0.2033	0.105	0.207	0.968	-0.1018	0.106	0.109	0.989	0	0.107	0.039	0.998	0.3099	0.110	0.312	1
0.3	0	0.0065	0.232	0.208	0.067	0.0054	0.238	0.215	0.183	0.0068	0.245	0.227	0.354	0	0.273	0.249	0.870
	0.1	-0.0505	0.214	0.191	0.181	-0.0275	0.205	0.168	0.375	0.0047	0.205	0.166	0.572	0.0680	0.261	0.252	0.955
	0.2	-0.0823	0.199	0.188	0.287	-0.0446	0.184	0.147	0.509	0.0041	0.182	0.136	0.705	0.1109	0.248	0.258	0.980
	0.4	-0.1210	0.175	0.187	0.477	-0.0647	0.156	0.126	0.695	0.0037	0.153	0.100	0.851	0.1678	0.223	0.269	0.995
	0.6	-0.1469	0.155	0.189	0.637	-0.0770	0.137	0.115	0.818	0.0034	0.133	0.076	0.927	0.2100	0.198	0.279	0.999
	0.8	-0.1687	0.135	0.192	0.776	-0.0865	0.121	0.108	0.907	0.0034	0.118	0.057	0.968	0.2486	0.169	0.289	1
	1	-0.1934	0.102	0.197	0.951	-0.0954	0.103	0.103	0.987	0.0033	0.103	0.039	0.998	0.3036	0.105	0.306	1
0.6	0	0.0106	0.219	0.194	0.063	0.0102	0.225	0.201	0.176	0.0124	0.232	0.213	0.362	0.0074	0.260	0.237	0.894
	0.1	-0.0465	0.204	0.181	0.171	-0.0245	0.193	0.157	0.372	0.0093	0.193	0.155	0.589	0.0758	0.249	0.244	0.969
	0.2	-0.0783	0.190	0.179	0.275	-0.0421	0.174	0.138	0.514	0.0081	0.171	0.126	0.717	0.1176	0.237	0.252	0.987
	0.4	-0.1168	0.169	0.180	0.463	-0.0621	0.149	0.119	0.697	0.0067	0.145	0.093	0.861	0.1727	0.214	0.266	0.998
	0.6	-0.1425	0.150	0.183	0.612	-0.0742	0.132	0.109	0.821	0.0062	0.127	0.072	0.928	0.2131	0.190	0.276	0.999
	0.8	-0.1639	0.131	0.187	0.762	-0.0833	0.117	0.103	0.904	0.0058	0.114	0.055	0.971	0.2496	0.163	0.286	1
	1	-0.1880	0.101	0.192	0.939	-0.0917	0.101	0.100	0.983	0.0058	0.102	0.040	0.995	0.3008	0.103	0.303	1
1	0	0.0145	0.209	0.188	0.058	0.0148	0.216	0.193	0.176	0.0175	0.223	0.207	0.369	0.0138	0.251	0.231	0.909
	0.1	-0.0421	0.197	0.176	0.160	-0.0210	0.186	0.151	0.370	0.0133	0.186	0.149	0.594	0.0815	0.241	0.241	0.973
	0.2	-0.0734	0.185	0.174	0.267	-0.0389	0.168	0.133	0.514	0.0114	0.165	0.122	0.724	0.1224	0.230	0.250	0.989
	0.4	-0.1118	0.165	0.176	0.445	-0.0590	0.145	0.116	0.692	0.0095	0.140	0.090	0.862	0.1757	0.208	0.264	0.998
	0.6	-0.1375	0.148	0.179	0.593	-0.0712	0.129	0.107	0.813	0.0085	0.124	0.071	0.926	0.2145	0.185	0.275	0.999
	0.8	-0.1590	0.129	0.183	0.739	-0.0803	0.115	0.101	0.899	0.0078	0.112	0.055	0.968	0.2498	0.159	0.285	1
	1	-0.1836	0.100	0.188	0.920	-0.0887	0.100	0.097	0.978	0.0076	0.100	0.041	0.995	0.2988	0.101	0.301	1
